# Enolization rates control mono- *versus* di-fluorination of 1,3-dicarbonyl derivatives†
†Electronic supplementary information (ESI) available. See DOI: 10.1039/c9sc04185k


**DOI:** 10.1039/c9sc04185k

**Published:** 2019-09-16

**Authors:** Neshat Rozatian, Andrew Beeby, Ian W. Ashworth, Graham Sandford, David R. W. Hodgson

**Affiliations:** a Chemistry Department , Durham University , South Road , Durham , DH1 3LE , UK . Email: d.r.w.hodgson@durham.ac.uk; b AstraZeneca , Pharmaceutical Technology & Development , Macclesfield , SK10 2NA , UK

## Abstract

Fluorine-containing 1,3-dicarbonyl derivatives are essential building blocks for drug discovery and manufacture. To understand the factors that determine selectivity between mono- and di-fluorination of 1,3-dicarbonyl systems, we have performed kinetic studies of keto–enol tautomerism and fluorination processes. Photoketonization of 1,3-diaryl-1,3-dicarbonyl derivatives and their 2-fluoro analogues is coupled with relaxation kinetics to determine enolization rates. Reaction additives such as water accelerate enolization processes, especially of 2-fluoro-1,3-dicarbonyl systems. Kinetic studies of enol fluorination with Selectfluor™ and NFSI reveal the quantitative effects of 2-fluorination upon enol nucleophilicity towards reagents of markedly different electrophilicity. Our findings have important implications for the synthesis of α,α-difluoroketonic compounds, providing valuable quantitative information to aid in the design of fluorination and difluorination reactions.

## Abstract 2

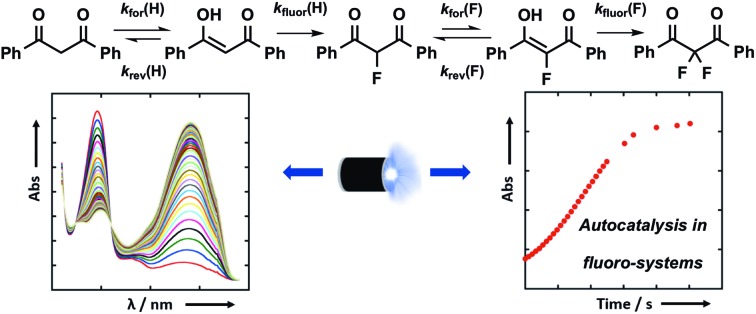
All rate constants for fluorination and enolization are determined.

## 1. Introduction

Fluorinated compounds have fundamental roles within the pharmaceutical, agrochemical and materials industries.^1^–^4^ The presence of a fluorine atom imparts profound effects upon the physical, chemical and biological properties of drugs and plant protection agents such as Prozac™, Lipitor®, ciprofloxacin and diclosulam.^5^ Such compounds are often synthesised from fluorine-containing building blocks;^6^ a key example is the antifungal agent voriconazole,^7^ which is synthesised from a 5-fluoropyrimidine intermediate that is prepared from a 2-fluoro-1,3-ketoester derivative. In this context, finding selective and efficient routes towards the fluorination of 1,3-dicarbonyl derivatives has been the subject of significant interest. Early work involved fluorinating reagents such as ClO_3_F,^8^ CF_3_OF,^9^ XeF_2_ (ref. 10,11) and CsSO_4_F;^12^ however, the low selectivities, difficulties regarding preparation, high reactivities and toxicities of these reagents halted their adoption in discovery and manufacturing processes. Elemental fluorine (F_2_) has been successfully used for the fluorination of 1,3-dicarbonyl systems, using both batch and flow techniques on laboratory and manufacturing scales, but this reagent requires specialist handling techniques that are not readily available in most laboratories.^13^–^18^

**Fig. 1:**
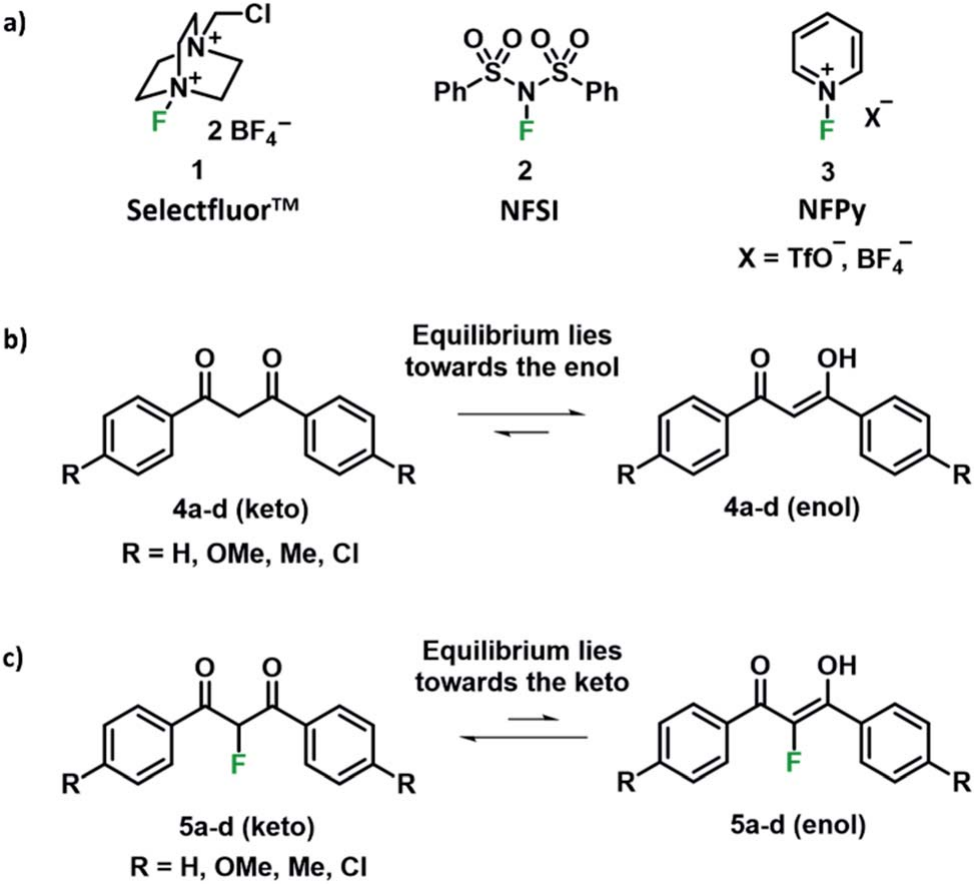
(a) Commonly used N–F reagents: Selectfluor™, *N*-fluorobenzenesulfonimide (NFSI) and *N*-fluoropyridinium salts (NFPy). (b) Tautomerism in compounds **4a–d**. (c) Tautomerism in the fluorine-containing compounds **5a–d**.

With the introduction of shelf-stable, crystalline electrophilic fluorinating reagents of the N–F class, such as Selectfluor™, NFSI and *N*-fluoropyridinium salts (Fig. 1a), that do not present any handling problems, numerous reports followed regarding the electrophilic fluorination of 1,3-dicarbonyl derivatives. Procedures include catalyst-free reactions,^19^ microwave-assisted methods,^20^ transition metal (Ti and Ru) catalysed methods,^21^–^23^ solvent-free reactions assisted by milling,^24^,^25^ fluorinations in ionic liquids,^26^ and reactions conducted in water.^27^,^28^ In many cases, difficulties in controlling mono- *versus* difluorination were reported, leading to challenging separations of the product mixtures. Therefore, finding synthetic routes that allow selective fluorination by such widely used reagents would be of great use.

Bioactive compounds bearing CF_2_ groups are found in both drugs and agrochemicals (*e.g.* gemcitabine, pantoprazole, sedaxane).^29^ In particular, carbonyl and dicarbonyl compounds containing α,α-difluoromethylene moieties are highly desirable bioactive compounds. When adjacent to a carbonyl group, the difluoromethylene moiety greatly increases the electrophilicity of the carbonyl group, leading to very facile nucleophilic additions. These include the additions of nucleophilic residues of enzyme active sites to α,α-difluoroketonic compounds,^30^,^31^ which have led to the application of α,α-difluoroketones as enzyme inhibitors.^32^–^34^ For example, difluorostatone compounds have been identified as potent inhibitors of HIV-1 protease^35^ and of a serine protease in the malaria parasite.^36^

Despite the importance of organofluorine compounds in the life sciences, very few kinetics studies on fluorination reactions are present in the literature and there have been no quantitative studies on the introduction of two fluorine atoms to form a difluoromethylene unit. Furthermore, although water and formic acid have been used as solvents or co-solvents in electrophilic fluorination reactions,^13^,^28^ their effects upon keto–enol tautomerism of 1,3-dicarbonyl derivatives and subsequent fluorinations are not fully understood. We have previously reported a quantitative reactivity scale for electrophilic N–F fluorinating reagents, where absolute and relative rate constants were determined for the monofluorination of a series of *para*-substituted 1,3-diaryl-1,3-dicarbonyl derivatives by 10 different N–F reagents, in acetonitrile.^37^ An independent study reported at the same time by Mayr *et al.*^38^ provided a scale of electrophilicity of the N–F reagents which was in very good agreement. A recent report by Nelson and co-workers^39^ gave further evidence supporting the S_N_2 mechanism for fluorinations by Selectfluor™.

We were intrigued by the keto–enol tautomerism phenomena displayed by our aromatic 1,3-dicarbonyls and their monofluorinated analogues (Fig. 1b and c) and how we could take advantage of their photochemical interconversion properties to study tautomerism and fluorination processes. The mechanism of fluorination proceeds *via* reaction of the enol tautomer with an electrophilic fluorinating reagent,^14^,^19^ so a full understanding of the factors that affect keto–enol tautomerism would be beneficial in improving selective mono- and difluorinations of 1,3-dicarbonyls. The photochemistry and photo-physics of 1,3-diaryl-1,3-dicarbonyl derivatives have been extensively studied.^40^,^41^ In the 1970s, the groups of Markov^42^,^43^ and Mazur^44^–^46^ reported photoisomerization of 1,3-dicarbonyl compounds, whereby the keto–enol equilibrium was perturbed towards the keto tautomer upon irradiation. This process reverses to attain the tautomeric equilibrium by a non-photochemical reaction in darkness. The effects of solvents and additives (ethanol, triethylamine) on the rate of photoketonization were studied by Mazur *et al.*;^44^ however, relaxation kinetics that provided insights into enolization rates were not performed. We therefore identified the photoketonization approach as a means of studying the kinetics of enolization within our nucleophile systems.

Our efforts towards the quantification of the factors which affect mono- *versus* difluorination are two-fold; in the first instance, we focus on the effects of different reaction conditions on the keto–enol tautomerism of the 1,3-diaryl-1,3-dicarbonyl derivatives **4a–d** and **5a–d**. Secondly, we explore and discuss the kinetics of fluorination of enols **4a–d** and fluoroenols **5a–d** and the effects of solvent composition upon these processes.

## 2. Results and discussion

### 2.1 Kinetics of keto–enol tautomerism in compounds **4a–d**

**Fig. 2:**
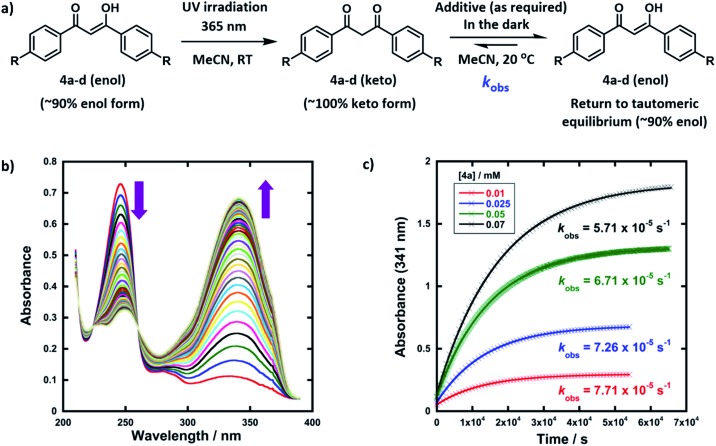
(a) Reaction scheme for photoketonization (step 1) and relaxation (step 2) of compounds **4a–d** in MeCN. (b) Time-arrayed multi-wavelength analysis for relaxation of **4a** (0.025 mM) in the dark, each spectrum acquired at 15 min intervals at 20 °C. (c) Relaxation of **4a-keto** at different concentrations (0.01 mM, 0.025 mM, 0.05 mM and 0.07 mM); *k*_obs_ values obtained at each concentration of **4a** are shown.

The enol forms of 1,3-diaryl-1,3-dicarbonyl derivatives **4a–d** show markedly different absorption spectra to their keto tautomers and are thus convenient systems for the study of tautomerization kinetics by UV-vis spectrophotometry. Compounds **4a–d** were synthesised using previously reported methods,^47^ and they exist predominantly in their enol forms (∼90% in MeCN). The enol tautomers were converted to their keto forms by irradiation of solutions of **4a–d** in quartz cuvettes using a 0.5 W UV LED lamp at 365 nm (Fig. 2a). Spectrophotometric monitoring of the photoketonization of each system showed that these processes took several hours (see ESI Section 3.3† for corresponding spectra). The re-equilibration (relaxation) kinetics of **4a-keto** in the dark were monitored using time-arrayed multi-wavelength analysis (Fig. 2b). As relaxation occurred, the enol absorbance band at *λ*_max_ = 341 nm increased while the keto absorbance band at *λ*_max_ = 250 nm decreased. The tautomeric equilibrium was regained after ∼14 hours, and clean isosbestic points were observed during both the photoketonization and the relaxation processes showing that there was no detectable build-up of additional intermediates during the tautomerization processes on the timescales that we monitored. The re-equilibration of **4a-keto** was studied at four different concentrations and observed first-order rate constants (*k*_obs_) were obtained from plots of absorbance at *λ*_max_ = 341 nm over time (Fig. 2c). When the concentration of **4a** was doubled, there was a small decrease in *k*_obs_, which could reflect interaction between substrate molecules at higher concentrations.^48^

In order to gain insight into the potential effects of species that are present in widely-employed electrophilic fluorination protocols upon enolization, we explored the effects of water, formic acid, DABCO and ClCH_2_-DABCO^+^BF_4_^–^ upon re-equilibration kinetics. In addition, because keto–enol equilibration is a reversible process with significant proportions of both keto- and enol-tautomers being present at equilibrium, we also considered the effects of additives upon equilibrium position (*K*_e_). The observed rate constants *k*_obs_ for re-equilibration of **4a-keto** and the equilibrium constants *K*_e_ in the presence of the additives are summarized in Table 1. Forward and reverse rate constants *k*_for_(H) and *k*_rev_(H), respectively, were estimated from *k*_obs_ values using measured *K*_e_ and eqn (1) and (2):

1 *k*_obs_ = *k*_for_(H) + *k*_rev_(H)

2 *K*_e_ = *k*_for_(H)/*k*_rev_(H)

**Table 1:** Summary of ketone relaxation of non-fluorinated 1,3-dicarbonyl systems. The *k*_obs_ values for relaxation of photo-ketonized forms of **4a–d** (0.025 mM) were determined in MeCN at 20 °C in the presence of additives. Percentages represent volumes of additive in MeCN. Equilibrium constants *K*_e_ were determined by NMR spectroscopy or by linear interpolation, extrapolation or averaging of the measured data. Forward and reverse rate constants, *k*_for_(H) and *k*_rev_(H), for enolization and ketonization processes, of the non-fluorinated 1,3-dicarbonyl systems, respectively were calculated using eqn (1) and (2)

Aryl substituent	Additive	Quantity of additive	*k*_obs_/s^–1^	Approx. *t*_1/2_	*K*_e_(H)	*k*_for_(H)/s^–1^	*k*_rev_(H)/s^–1^	*k*_for_(H) {with additive}/*k*_for_(H) {MeCN}
**4a** (R = H)	None	—	7.26 × 10^–5^	2.7 h	10.5^*a*^	6.63 × 10^–5^	6.31 × 10^–6^	1.0
	Water	15%	8.44 × 10^–5^	2.3 h	7.8^*c*^	7.48 × 10^–5^	9.59 × 10^–6^	1.1
		20%	1.79 × 10^–4^	1.1 h	6.9^*b*^	1.56 × 10^–4^	2.27 × 10^–5^	2.4
		25%	1.84 × 10^–4^	1 h	6.6^*c*^	1.60 × 10^–4^	2.42 × 10^–5^	2.4
		35%	3.39 × 10^–4^	34 min	6.3^*c*^	2.93 × 10^–4^	4.64 × 10^–5^	4.4
		50%	7.29 × 10^–4^	15 min	5.7^*b*^	6.20 × 10^–4^	1.09 × 10^–4^	9.4
	Formic acid	0.5%	1.92 × 10^–3^	6 min	10.3^*c*^	1.75 × 10^–3^	1.70 × 10^–4^	26
		1%	4.16 × 10^–3^	3 min	10.0^*b*^	3.78 × 10^–3^	3.78 × 10^–4^	57
		2%	4.89 × 10^–3^	2 min	10.5^*b*^	4.46 × 10^–3^	4.25 × 10^–4^	67
		3%	6.29 × 10^–3^	2 min	9.5^*b*^	5.69 × 10^–3^	5.99 × 10^–4^	86
	DABCO	2.5 μM	2.92 × 10^–3^	4 min	6.7^*c*^	2.54 × 10^–3^	3.80 × 10^–4^	39
		12.5 μM	1.34 × 10^–2^	1 min	6.7^*c*^	1.17 × 10^–2^	1.74 × 10^–3^	176
		25 μM (1 eq.)	2.49 × 10^–2^	30 s	6.7^*b*^	2.17 × 10^–2^	3.24 × 10^–3^	327
		37.5 μM	4.08 × 10^–2^	17 s	6.7^*c*^	3.55 × 10^–2^	5.30 × 10^–3^	536
		50 μM	5.22 × 10^–2^	13 s	6.7^*b*^	4.54 × 10^–2^	6.78 × 10^–3^	685
	ClCH_2_-DABCO^+^BF_4_^–^	12.5 μM	1.04 × 10^–4^	2 h	9.1^*c*^	9.37 × 10^–5^	1.03 × 10^–5^	1.4
		25 μM (1 eq.)	1.20 × 10^–4^	1.6 h	9.1^*b*^	1.08 × 10^–4^	1.19 × 10^–5^	1.6
		50 μM	1.32 × 10^–4^	1.5 h	9.1^*c*^	1.19 × 10^–4^	1.31 × 10^–5^	1.8
		625 μM	1.00 × 10^–4^	2 h	9.1^*c*^	9.01 × 10^–5^	9.90 × 10^–6^	1.4
		1.25 mM	5.12 × 10^–5^	4 h	9.1^*c*^	4.61 × 10^–5^	5.07 × 10^–6^	0.7
		2.5 mM	2.04 × 10^–5^	9 h	9.1^*c*^	1.84 × 10^–5^	2.02 × 10^–6^	0.3
	20% water and ClCH_2_-DABCO^+^BF_4_^–^	20%/12.5 μM	2.05 × 10^–4^	1 h	6.9^*c*^	1.79 × 10^–4^	2.59 × 10^–5^	2.7
	^*n*^Bu_4_N^+^BF_4_^–^	240 mM	1.44 × 10^–4^	1.3 h	9.1^*b*^	1.30 × 10^–4^	1.43 × 10^–5^	2.0

**4b** (R = OMe)	None	—	1.29 × 10^–5^	15 h	4.0^*a*^	1.03 × 10^–5^	2.58 × 10^–6^	1.0
	Water	50%	1.47 × 10^–4^	1.3 h	3.7^*b*^	1.16 × 10^–4^	3.13 × 10^–5^	11
	Formic acid	2%	8.27 × 10^–3^	1.4 min	5.3^*b*^	6.96 × 10^–3^	1.31 × 10^–3^	674
	DABCO	2.5 μM	8.24 × 10^–4^	14 min	5.1^*b*^	6.89 × 10^–4^	1.35 × 10^–4^	67
	ClCH_2_-DABCO^+^BF_4_^–^	50 μM	7.11 × 10^–6^	27 h	5.0^*b*^	5.93 × 10^–6^	1.19 × 10^–6^	0.6

**4c** (R = Me)	None	—	5.67 × 10^–5^	3.5 h	8.0^*a*^	5.04 × 10^–5^	6.30 × 10^–6^	1.0
	Water	50%	3.15 × 10^–4^	37 min	8.0^*d*^	2.80 × 10^–4^	3.50 × 10^–5^	5.6
	DABCO	2.5 μM	8.03 × 10^–4^	14 min	8.0^*d*^	7.14 × 10^–4^	8.92 × 10^–5^	14

**4d** (R = Cl)	None	—	1.07 × 10^–4^	2 h	12.5^*a*^	9.91 × 10^–5^	7.93 × 10^–6^	1.0
	Water	50%	2.13 × 10^–3^	5 min	12.5^*d*^	1.97 × 10^–3^	1.58 × 10^–4^	20
	DABCO	2.5 μM	7.69 × 10^–3^	1.5 min	12.5^*d*^	7.12 × 10^–3^	5.70 × 10^–4^	72
	ClCH_2_-DABCO^+^BF_4_^–^	25 μM	7.35 × 10^–5^	2.6 h	12.5^*d*^	6.81 × 10^–5^	5.44 × 10^–6^	0.7

^*a*^Measured by ^1^H NMR spectroscopy in MeCN-*d*_3_.

^*b*^Measured by ^1^H NMR spectroscopy in MeCN-*d*_3_ in the presence of additive (for details see ESI Section 3.2†).

^*c*^Value based on average of measured values or interpolation of measured values.

^*d*^ *K*_e_(H) value was assumed to be the same as *K*_e_(H) in MeCN-*d*_3_ alone.

**Fig. 3:**
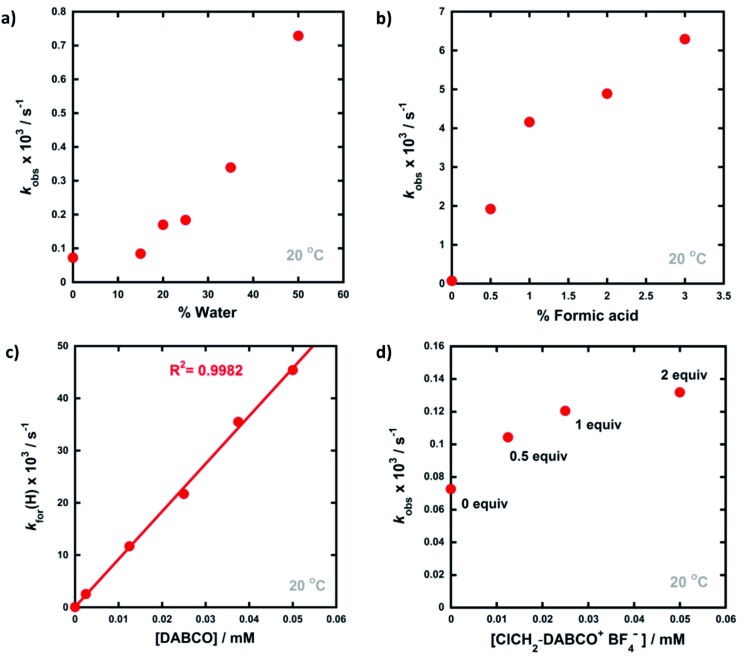
Trends observed in the rates of relaxation (*k*_obs_) of **4a** (0.025 mM) upon addition of different quantities of additives, in MeCN at 20 °C: (a) water, (b) formic acid, (c) DABCO, (d) 0.5–2 equivalents of ClCH_2_-DABCO^+^BF_4_^–^.

Given that the enol forms of **4a–d** are dominant at equilibrium, *k*_obs_ and *k*_for_(H) values are in the same order. With water as the additive (15–50% of the reaction mixture by volume), increased rates of re-enolization were observed (Fig. 3a), with a 1 : 1 MeCN/water solvent system giving a 10-fold increase in *k*_for_(H) compared to MeCN. The position of the keto–enol equilibrium changed marginally upon moving from MeCN to 1 : 1 MeCN/water, with *K*_e_ values of 10.5 and 5.7 respectively. This is consistent with previous studies on 1,3-dicarbonyl systems which show limited variations of *K*_e_ values upon changes from single- to mixed-polar solvent systems.^49^ Photophysical studies on di-substituted 1,3-diphenyl-1,3-propanedione compounds have shown that MeCN supports very slow exchange between tautomeric states, whereas protic solvents, including MeCN-water mixtures, enhance rates significantly.^50^,^51^ Water is often used as a solvent or co-solvent in fluorination reactions to aid solubility of Selectfluor™.^28^ Our studies show that the solubility limit of Selectfluor™ in water is ∼500 mM, compared to ∼50 mM in MeCN. Here, we have shown that the addition of water also increases the rate of enolization, facilitating the conversion of the small amounts of residual diketone to the nucleophilic enol tautomer, which reacts with the fluorinating reagent.

The addition of small amounts of formic acid had limited effects on the position of the keto–enol equilibria with all *K*_e_ values being ∼10, however, greatly enhanced rates of keto-to-enol relaxation were observed (Fig. 3b). Thus, the rate constant for enolization *k*_for_(H) increased 86-fold upon addition of 3% formic acid in comparison to MeCN alone.

The addition of DABCO also increased the relaxation rates significantly. For example, with one equivalent (25 μM), a 330-fold acceleration of the enolization process *k*_for_(H) was observed. Even with 0.1 equivalents (2.5 μM) of DABCO, the tautomeric equilibrium was regained rapidly. When *k*_for_(H) values for relaxation were plotted against DABCO concentration (Fig. 3c), a simple linear (*i.e.* first order) correlation was observed, giving the second-order rate constant, *k*_2_ = 9.13 × 10^2^ M^–1^ s^–1^. In terms of basicity, DABCO (p*K*_aH_(MeCN) = 18.29)^52^ is insufficiently basic to quantitatively deprotonate **4a-keto** (for **4a-keto** estimated p*K*_a_(MeCN) = p*K*_a_(DMSO) + 12.9 (ref. 53) = 13.4 (ref. 54) + 12.9 = 26.3). Thus our data suggest that DABCO may operate as a general base catalyst.

Upon delivery of electrophilic fluorine, N–F reagents give amines as by-products, which could promote keto–enol tautomerism, and hence the rate of fluorination, if they remain unprotonated. Fluorination reactions using Selectfluor™ result in the formation of ClCH_2_-DABCO^+^BF_4_^–^ and when 0.5 to 2 equivalents of ClCH_2_-DABCO^+^BF_4_^–^ were added to **4a-keto**, very small (1.4 to 2-fold) increases in *k*_obs_ and *k*_for_(H) were observed (Fig. 3d). However, the addition of greater quantities of ClCH_2_-DABCO^+^BF_4_^–^ (25–100 equivalents) resulted in reduced *k*_for_(H) values (see ESI Section 3.4.5†). For example, with 100 equivalents of ClCH_2_-DABCO^+^, *k*_for_(H) was reduced three-fold. While the ClCH_2_-DABCO^+^ cation is unlikely to remain unprotonated and thus will be unable to function as a base, this series of experiments suggested the possibility of salt effects upon the relaxation processes. Consequently, relaxation experiments were performed in the presence of 2–100 equivalents of LiBF_4_ and similar reductions in *k*_for_(H) were observed (for related spectra see ESI Section 3.4.7†). Li salts are known to form chelates with 1,3-diketones,^54^ thus we explored the effects of adding ^*n*^Bu_4_NBF_4_, a non-chelating salt. In order to mimic the salt concentrations in synthetic-scale processes, we studied the effect of adding 240 mM ^*n*^Bu_4_NBF_4_ to solutions of **4a-keto**. Under these conditions, *k*_for_(H) increased 2-fold in comparison to experiments in the absence of salts. In summary, the effects of ‘spent’ Selectfluor™ (*i.e.* ClCH_2_-DABCO^+^BF_4_^–^) and other ionic species upon enolization kinetics of **4a–d** are measurable, but marginal and potentially complex in nature.

We also explored the effects of the amine derivative of NFSI, dibenzenesulfonimide ((PhSO_2_)_2_NH, p*K*_a_(MeCN) ∼11.3)^55^ upon the rate of relaxation of **4a-keto**. Interestingly, the presence of 5 equivalents of (PhSO_2_)_2_NH resulted in a 40-fold decrease in the relaxation rate constant rate, however, 10 equivalents of (PhSO_2_)_2_NH gave only a 16-fold decrease (see ESI Section 3.4.8–3.4.9†). The addition of the conjugate base form, (PhSO_2_)_2_N^–^Na^+^, also showed a similar effect, with one equivalent causing a significant reduction in relaxation rate and larger concentrations showing less-pronounced reductions. In this case, the reduction in *k*_for_(H) is likely due to chelation of the Na^+^ ion to diketone **4a**, an interaction previously described by Bordwell.^54^

The relaxation kinetics of keto forms of **4b–d** were explored using the same photoketonization procedure, in the presence and absence of additives, and corresponding *k*_obs_, *K*_e_, *k*_for_(H) and *k*_rev_(H) values are reported in Table 1. There were little variations in measured *K*_e_ values for **4b** across the range of conditions that we employed. For **4c** and **4d**, we assumed that the *K*_e_(H) values in the presence of additives would be the same as *K*_e_(H) in MeCN-*d*_3_ alone under conditions where measured values were not obtained. The effects of the *para*-substituents within **4a–d** on *k*_for_(H) in MeCN were studied by Hammett correlation analysis. The use of *σ*_p_^+^ values in the construction of the Hammett plot gave better correlations than with *σ*_p_ values (see ESI Section 3.5†). A *ρ*^+^ value of +1.06 was obtained, where this positive value indicates small increases in electron density on the aryl rings of the substrates during the limiting C–H removal step of enolization. Compound **4d** (R = Cl) relaxed most rapidly, whereas compound **4b** (R = OMe) was the slowest, which suggests that rate limiting proton transfer from carbon proceeds towards an anionic intermediate rather than through pre-protonation of the ketone.

### 2.2 Kinetics of keto–enol tautomerism in compounds **5a–d**

**Fig. 4:**
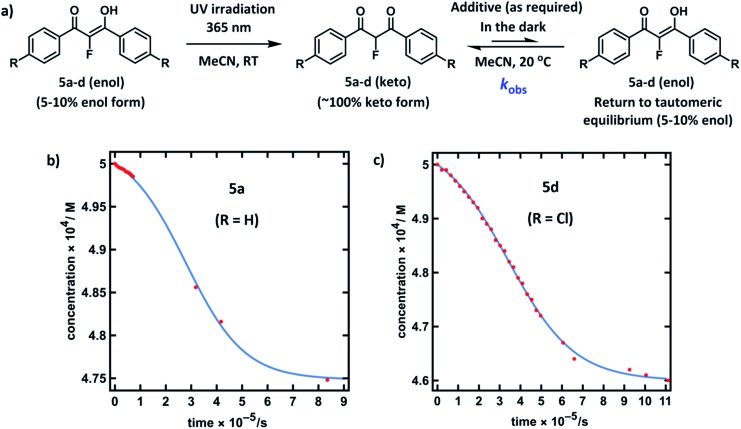
(a) Reaction scheme for photoketonization (step 1) by irradiation with a 0.5 W UV LED lamp at 365 nm for 4–5 hours, followed by relaxation (step 2) of **5a–d** in MeCN. (b) Plot of [**5a-keto**] *versus* time obtained from a time-arrayed single-wavelength kinetic analysis for relaxation of **5a-keto** showing the return to the tautomeric equilibrium (0.50 mM, 20 °C, spectra acquired over 11 days). (c) Plot of [**5d-keto**] *versus* time obtained from a time-arrayed single-wavelength kinetic analysis for relaxation of **5d-keto** showing the return to the tautomeric equilibrium (0.50 mM, 20 °C, spectra acquired over 13 days).

Compounds **5a–d** were synthesised using our previously reported methods,^37^ in good yields. Following purification by recrystallization, we obtained the fluorinated 1,3-diaryl-1,3-dicarbonyls as mixtures of both keto and enol tautomers, where the keto form comprised ∼95% of **5a** and **5b**, and ∼90% of **5c** and **5d** (as determined by ^19^F NMR spectroscopy in MeCN-*d*_3_, see ESI Section 3.2†). We determined *K*_e_ values for **5a** across a range of additives and, in a similar vein to the non-fluorinated systems, we found *K*_e_ to be broadly constant. The fluoroenol- and fluoroketo-tautomers of **5a–d** have distinct absorbance bands at ∼350 nm and ∼250 nm, respectively. Therefore, in much the same way as for compounds **4a–d**, we were able to monitor the tautomerism processes of the fluorinated derivatives *via* changes in absorbance of the fluoroenol tautomers. Photoketonization experiments were conducted on 0.50 mM solutions of **5a–d** in MeCN in the absence of additives (Fig. 4a). Following irradiation, spectrophotometric kinetic assays for relaxation were conducted, and they showed very slow restoration of the thermodynamic ratio between the two tautomeric forms. Plots of diketone concentration *versus* time for **5a–d** were constructed. In the case of **5a** and **5d** (Fig. 4b and c), sigmoidal behaviours were clearly discernible, which suggested autocatalysis of the processes, and fitting of the data to a model for reversible autocatalysis gave strong support for this hypothesis (for kinetic fittings performed using Wolfram Mathematica see ESI Sections 3.9.1 and 3.12.1†). In the case of **5b** and **5c**, reaction progress was extremely slow, and *k*_obs_ values were estimated using an initial rates approach (see ESI Sections 3.10.1 and 3.11.1†).

We then explored the effects of additives on the rates of relaxation of diketone tautomers of **5a–d**, and the corresponding *k*_for_(F) and *k*_rev_(F) values were obtained *via K*_e_ values (Table 2). In general, the effects of additives upon *k*_for_(F) were much greater than for the non-fluorinated series **4a–d**. With 20% water in MeCN, **5a-keto***k*_for_(F) was 160-fold larger than in the absence of water, whereas for system **4a-keto**, only a 2.4-fold enhancement in *k*_for_(H) was observed. When the quantity of water in MeCN was increased to 50%, *k*_for_(F) for **5a-keto** was further increased to 930-fold greater than in the absence of water. For compounds **5b** and **5d**, *k*_for_(F) increased 3000-fold in 50% water, while **5c** showed a 10 000-fold increase. Addition of formic acid (3% in MeCN) led to an increase in *k*_for_(F) of 220-fold, whereas DABCO proved to be an effective agent for de-fluorination of the substrate **5a** (see ESI Section 3.2.1† for related spectra, and previous reports^56^,^57^ of bromomalonitriles acting as brominating agents). ‘Spent’ Selectfluor™ (ClCH_2_-DABCO^+^BF_4_^–^, 0.025 mM) offered a 4-fold increase in *k*_for_(F), whereas the increase in *k*_for_(H) for **4a** with this additive was ∼2-fold, and only marginally discernible above salt-related medium effects (see ESI Section 3.9† for spectra).

**Table 2:** Summary of diketone relaxation of 2-fluorinated-1,3-dicarbonyl systems. The *k*_obs_ values for relaxation of photoketonized forms of **5a–d** (0.50 mM) were determined in MeCN at 20 °C in the presence of additives. Percentages represent volumes of additive in MeCN. Equilibrium constants *K*_e_ were determined by NMR spectroscopy or by linear interpolation, extrapolation or averaging of the measured data. Forward and reverse rate constants, *k*_for_(F) and *k*_rev_(F), for enolization and ketonization processes, of the 2-fluorinated-1,3-dicarbonyl systems, respectively were calculated using eqn (1) and (2)

Aryl substituent	Additive	Quantity of additive	*k*_obs_/s^–1^	Approx. *t*_1/2_	*K*_e_(F)	*k*_for_(F)/s^–1^	*k*_rev_(F)/s^–1^	*k*_for_(F){with additive}/*k*_for_(F){MeCN}
**5a** (R = H)	None	—	—^*a*^	—	0.053^*b*^	3.66 × 10^–8^ (1.58 × 10^–2^)^*c*^	6.91 × 10^–7^ (0.298)^*c*^	1.0
	Water	10%	4.98 × 10^–5^	4 h	0.053^*d*^	2.49 × 10^–6^	4.73 × 10^–5^	68
		20%	1.19 × 10^–4^	1.6 h	0.053^*d*^ ^,^^*e*^	5.95 × 10^–6^	1.13 × 10^–4^	163
		30%	2.23 × 10^–4^	0.9 h	0.053^*d*^	1.12 × 10^–5^	2.12 × 10^–4^	305
		40%	3.77 × 10^–4^	0.5 h	0.053^*d*^	1.89 × 10^–5^	3.58 × 10^–4^	515
		50%	6.78 × 10^–4^	0.3 h	0.053^*b*^	3.39 × 10^–5^	6.44 × 10^–4^	926
	Formic acid	3%	1.64 × 10^–4^	1.2 h	0.053^*b*^	8.20 × 10^–6^	1.56 × 10^–4^	224
	DABCO	2.5 μM	1.42 × 10^–3^	8 min	—^*f*^	—^*f*^	—^*f*^	—^*f*^
	ClCH_2_-DABCO^+^BF4^–^	12.5 μM	1.01 × 10^–6^	8 d	0.053^*d*^	5.05 × 10^–8^	9.60 × 10^–7^	1.4
		25 μM (1 eq.)	2.93 × 10^–6^	2.7 d	0.053^*d*^	1.47 × 10^–7^	2.78 × 10^–6^	4.0
	20% water and ClCH_2_-DABCO^+^BF_4_^–^	20%/12.5 μM	1.91 × 10^–4^	1 h	0.053^*d*^	9.55 × 10^–6^	1.81 × 10^–4^	261
	^*n*^Bu_4_N^+^BF_4_^–^	240 mM	8.38 × 10^–5^	2.3 h	0.043^*g*^	3.42 × 10^–6^	8.04 × 10^–5^	94

**5b** (R = OMe)	None	—	1.46 × 10^–7^^*h*^	60 d	0.020^*b*^	2.92 × 10^–9^ (n.d.)^*c*^	1.43 × 10^–7^ (n.d.)^*c*^	1.0
	Water	20%	3.22 × 10^–5^	6 h	0.033^*d*^	1.04 × 10^–6^	3.12 × 10^–5^	355
		30%	5.39 × 10^–5^	3.6 h	0.040^*d*^	2.06 × 10^–6^	5.18 × 10^–5^	706
		40%	9.23 × 10^–5^	2.1 h	0.046^*d*^	4.07 × 10^–6^	8.82 × 10^–5^	1396
		50%	1.71 × 10^–4^	1.1 h	0.053^*b*^	8.55 × 10^–6^	1.62 × 10^–4^	2928
	Formic acid	2%	1.73 × 10^–5^	11 h	0.031^*b*^	5.19 × 10^–7^	1.68 × 10^–5^	178
	DABCO	2.5 μM	1.32 × 10^–5^	15 h	0.020^*b*^	2.64 × 10^–7^	1.29 × 10^–5^	90

**5c** (R = Me)	None	—	8.64 × 10^–8^^*h*^	90 d	0.149^*b*^	1.12 × 10^–8^	7.52 × 10^–8^	1.0
	Water	50%	9.15 × 10^–4^	0.2 h	0.149^*i*^	1.19 × 10^–4^	7.96 × 10^–4^	10 594

**5d** (R = Cl)	None	—	—^*a*^	—	0.087^*b*^	5.37 × 10^–8^ (1.11 × 10^–2^)^*c*^	6.18 × 10^–7^ (0.128)^*c*^	1.0
	Water	50%	1.97 × 10^–3^	6 min	0.087^*i*^	1.58 × 10^–4^	1.81 × 10^–3^	2936

^*a*^System displayed non-first order autocatalytic behaviour.

^*b*^Measured by ^19^F NMR spectroscopy in MeCN-*d*_3_ or MeCN-*d*_3_/D_2_O.

^*c*^Second order rate constant for autocatalytic process in units of M^–1^ s^–1^.

^*d*^Value based on average of measured values or interpolation of measured values.

^*e*^A ^19^F NMR spectroscopy measurement in 20% H_2_O/MeCN-*d*_3_ gave *K*_e_(F) = 0.042.

^*f*^Defluorination was observed.

^*g*^Measured in the presence of 300 mM ^*n*^Bu_4_N^+^BF_4_^–^.

^*h*^Extremely slow process, where rate constant was determined by initial rates method.

^*i*^ *K*_e_(F) for 50% H_2_O was assumed to be the same as *K*_e_(F) in MeCN-*d*_3_.

Overall, these data suggest that the tautomerization reactions of the fluoro-systems **5a–d** are accelerated much more significantly in the presence of polar additives than those of the non-fluorinated systems.

### 2.3 Kinetics of enol and fluoroenol fluorination

**Fig. 5:**
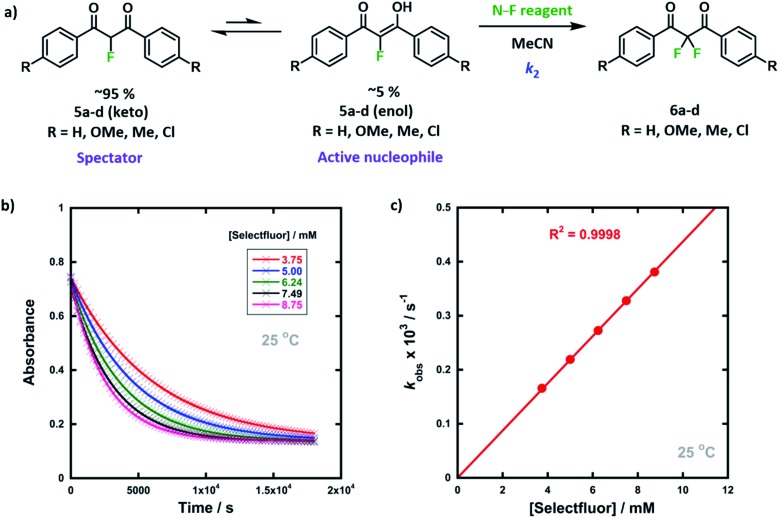
(a) Reaction scheme for fluorination reactions of 1,3-dicarbonyls **5a–d** with Selectfluor™ or NFSI in MeCN at controlled temperatures. (b) Exponential decays of absorbance of **5a-enol** at 350 nm with different concentrations of Selectfluor™, in MeCN at 25 °C. (c) Correlation of *k*_obs_ values for fluorination of **5a-enol** with [Selectfluor™], in MeCN at 25 °C.

We previously reported the kinetics of fluorination of enols **4a–d** in MeCN.^37^ We confirmed that fluorination of **5a** occurs only *via* the fluoroenol form with the aid of NMR experiments, and that the fluoroketo tautomer acts as a spectator during the addition of the second fluorine atom to **5a-enol**, owing to its slow enolization in MeCN. Here we explore the effects of additives upon the rates of fluorination of enols **4a–d** and make comparisons with their effects upon the rates of fluorination of fluoroenols **5a–d**. Together, these data allow us to explore the role of 2-fluorination upon enol nucleophilicity and potentially tune conditions, through the addition of *e.g.* water, towards favouring the formation of 2,2-difluoro-1,3-dicarbonyls **6a–d** (Fig. 5a). Furthermore, on account of the greatly increased rates of enolization in the presence of additives, we also take account of *in situ* enolization of **5a**.

By monitoring the decays in absorbance of the fluoroenol tautomers of **5a–d** at *λ* ∼350 nm, the kinetics of fluorination reactions were conveniently monitored by UV-vis spectrophotometry. To achieve pseudo-first order conditions, all kinetics experiments were carried out using excess electrophile. Clean exponential decays of absorbance of the nucleophile were observed in all runs in MeCN alone (representative examples are shown in Fig. 5). The first-order rate constants *k*_obs_ were obtained from the fitting of plots of absorbance *versus* time (Fig. 5b). When *k*_obs_ values were plotted against electrophilic fluorine concentration, linear (*i.e.* first order) correlations were observed (Fig. 5c), which projected cleanly through the origin in each case. The direct dependence upon electrophilic fluorine concentration demonstrates rate-limiting fluorination of the fluoroenol that is present in the mixture, and thus the slopes of these graphs gave the second-order rate constant *k*_2_ [M^–1^ s^–1^] according to the rate eqn (3). The rate constants for the reactions of **5a–d** with each fluorinating reagent are summarized in Table 3.3
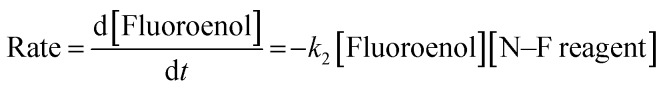



**Table 3:** Second-order rate constants (*k*_2_) for the reactions of Selectfluor™ **1** and NFSI **2** with nucleophiles **5a–d**, in MeCN at 25 °C, and relative rates compared to the reactions of Selectfluor™ and NFSI with **4a–d**

Nucleophile	Electrophile	*k*_2_ (25 °C)/M^–1^ s^–1^	*k*_rel_
**5a-enol** (R = H)	Selectfluor™ **1**	4.37 × 10^–2^	1.0 (1.1)^*a*^
	NFSI **2**	4.59 × 10^–4^	46
**5b-enol** (R = OMe)	Selectfluor™ **1**	6.77 × 10^–1^	1.1 (1.1)^*a*^
	NFSI **2**	6.11 × 10^–4^	4.4
**5c-enol** (R = Me)	Selectfluor™ **1**	1.32 × 10^–1^	1.1
**5d-enol** (R = Cl)	Selectfluor™ **1**	3.07 × 10^–2^	1.7
	NFSI **2**	2.47 × 10^–4^	43

^*a*^Using *k*_2_ values for reactions measured at 20 °C.

A Hammett plot was constructed for the reactions of fluoroenols **5a–d** with Selectfluor™ **1** (see ESI Section 3.13.5†). The use of *σ*_p_^+^ values led to a better correlation than with *σ*_p_ constants, and *ρ*^+^ = –1.5 was obtained, with *R*^2^ of > 0.99. This value is similar to the *ρ*^+^ values we obtained for fluorination of enols **4a–d** by several N–F reagents, including Selectfluor™ **1** and NFSI **2**.^37^ Activation parameters (Δ*G*^‡^, Δ*H*^‡^ and Δ*S*^‡^) were calculated from kinetic data obtained at different temperatures for the reactions of Selectfluor™ with **5a-enol** and **5b-enol** (see ESI Section 3.13.5†). As with our previous publication, the moderately negative values of Δ*S*^‡^, alongside the values for *ρ*^+^, support an S_N_2-type mechanism for the fluorination reactions.

The rates of fluorination of **5a–d** by Selectfluor™ **1** and NFSI **2** were compared with the rate constants that we previously obtained for the fluorinations of **4a–d**,^37^ using *k*_rel_ values, defined in eqn (4).4
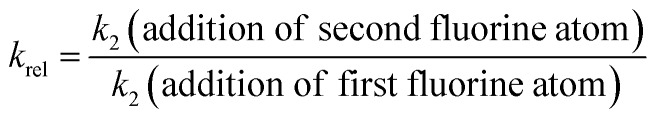



The *k*_2_ values obtained for fluorination of fluoroenols **5a–d** by Selectfluor™ are slightly higher than those for fluorination of enols **4a–d**, with *k*_rel_ values of 1.0–1.7 being observed. With NFSI, the rate enhancement is more pronounced, and the addition of the second fluorine atom to form the CF_2_ group is 46-fold faster for **5a-enol**, 4-fold greater for **5b-enol** and 43-fold faster for **5d-enol**.

One might expect that the presence of a highly electronegative fluorine atom would lead to a lowering of the nucleophilicity of the fluoroenol and much lower rates of fluorination. On the other hand, the strong pi-donor ability of the fluorine atom could lead to ground-state destabilization of fluorine atoms at sp^2^ centres and thus enhanced nucleophilicity of the fluoroenol. Our results suggest that a balance between these opposing effects is observed for fluorinations in MeCN with the more reactive Selectfluor™ system, however, with the less reactive NFSI reagent the fluoroenols **5a–d** are more reactive. The origins of this disparity could lie in the less early transition state structure that is to be expected from the less reactive NFSI system, coupled with the different charge state of the electrophile–nucleophile pair, and thus differing requirements for solvation.

Our findings of enhanced nucleophilicity for fluoroenols **5a–d** over enols **4a–d** align with studies conducted by Dolbier *et al.*^58^–^60^ on the kinetic impact of vinylic fluorine substituents upon cyclization reactions. They reported that the presence of a fluorine atom at an sp^2^ centre was disfavoured relative to the sp^3^ hybridised analogue, therefore, cyclization reactions occurred readily to form butadiene compounds. Chambers *et al.*^13^,^14^ reported that during electrophilic fluorination by elemental fluorine, the second fluorination step is much slower than the first. However, our results show that addition of the second fluorine atom proceeds at a rate that is similar to or even greater than the first fluorination step. The previously reported slow rate of difluorination is due to rate-limiting enolization of the mono-fluoro-diketone compound rather than the fluorination process itself. As we have shown in Section 2.2, the rate of enolization can be enhanced by the addition of catalytic amounts of water, salt, acid or base, which in turn contributes to an increase in the overall rate of the difluorination mechanism. Furthermore, in related carbanion systems, the reactivity was found to be enhanced by the presence of an α-fluorine atom compared to the non-fluorinated carbanion.^61^,^62^ Indeed, the effect of the α-fluorine was even greater in these studies, probably because of the increased repulsion between the oxyanionic charge and fluorine lone pairs in comparison to our systems.

We also studied the fluorinations of **4a-enol** and **5a-enol** by Selectfluor™ with 20% water in MeCN using an initial rates approach to overcome the complications associated with the presence of substantial keto–enol tautomerization and the formation of the hydrate of **6a** under these conditions (for spectra see ESI Sections 3.15 and 3.18†). The presence of water (20% in MeCN) during the fluorination of **5a-enol** gave a ∼50-fold larger second-order rate constant, *k*_2_, compared to without water, however, the *k*_2_ value for fluorination of **4a-enol** was little changed (Table 4). We also explored the effects of adding formic acid (3–20%) on the rates of fluorination of **5a-enol** and found that there was little effect on the *k*_2_ values (see ESI Section 3.16†). The presence of ^*n*^Bu_4_NBF_4_ (240 mM) during the fluorination of **5a-enol** by Selectfluor™ afforded a ∼2600-fold increase in rate, where this large increase is likely due to the combined effects of the salt itself and inadvertent addition of water owing to the hygroscopic nature of tetraalkylammonium systems.^63^ Taken together, the effects of additives upon *k*_2_ further support the idea of differential solvation and medium effects along the reaction co-ordinates of the fluorination and tautomerization processes of the enol and fluoroenol systems, however, their underlying origins are not clear at this stage.

**Table 4:** Second-order rate constants (*k*_2_) for the reactions of Selectfluor™ with nucleophiles **4a-enol** and **5a-enol** in 20% water in MeCN at 20 °C

Nucleophile	*k*_2_ (20 °C)/M^–1^ s^–1^
**4a-enol** (R = H)	2.49 × 10^–2^^*a*^
**5a-enol** (R = H)	1.43^*b*^

^*a*^In MeCN only, *k*_2_ = 2.68 × 10^–2^ M^–1^ s^–1^ at 20 °C for fluorination of **4a-enol**.^37^

^*b*^At 20 °C in MeCN only, *k*_2_ = 2.95 × 10^–2^ M^–1^ s^–1^ for fluorination of **5a-enol**.

### 2.4 Application of kinetic data to synthesis

**Fig. 6:**
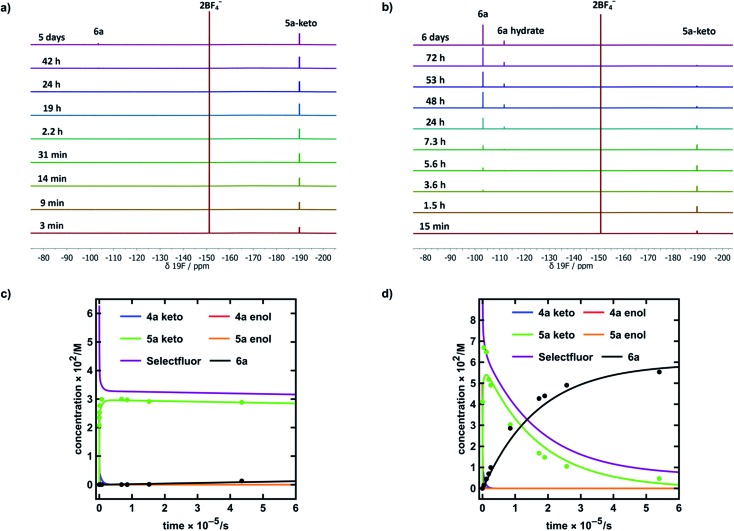
(a) ^19^F NMR time profile for the reaction between 1,3-dicarbonyl **4a** (30 mM) and Selectfluor™ (62.5 mM) in MeCN-*d*_3_. (b) ^19^F NMR time profile for the reaction between 1,3-dicarbonyl **4a** (59.5 mM) and Selectfluor™ (125 mM) in 20% water in MeCN-*d*_3_. (c) Integrated ^19^F NMR-time data for the reaction between **4a** (30 mM) and Selectfluor™ (62.5 mM) in MeCN-*d*_3_. (d) Integrated ^19^F NMR-time data for the reaction between **4a** (59.5 mM) and Selectfluor™ (125 mM) in 20% water in MeCN-*d*_3_. Some over-estimation of the concentration of **5a-keto** was evident in the NMR experiment and the origin of this is discussed further in the ESI Section 4.†

**Fig. 7:**
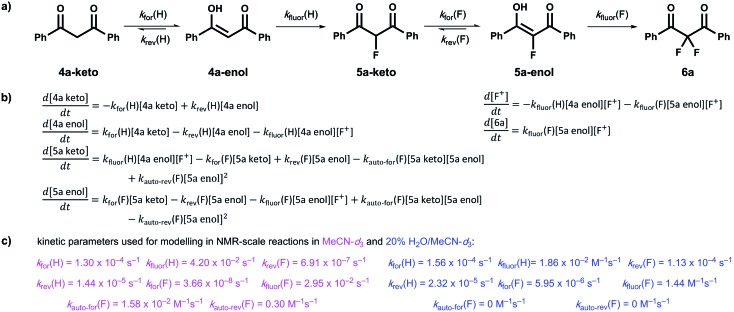
(a) Overall kinetic model for the difluorination of compound **4a** with Selectfluor™. (b) Differential representations for the rates of formation of each species within the kinetic model, where [F^+^] represents the concentration of Selectfluor™. (c) Rate constants used for kinetic fitting of fluorination processes in MeCN-*d*_3_ (pink) and in 20% water in MeCN-*d*_3_ (blue). Values for *k*_for_(H) and *k*_rev_(H) were based on those observed in the presence of 240 mM ^*n*^Bu_4_NBF_4_, where these values were chosen to mimic the effect of salt. When a similar approach was adopted for *k*_for_(F) and *k*_rev_(F), poor fitting was observed. We attribute this to the extreme sensitivity of the tautomerization processes of the fluoro-system **5a**, described by these parameters, to the presence of small amounts of water that arise from the highly hygroscopic nature of the tetrabutylammonium salt.

Our kinetic studies show that additives, such as water, facilitate enolization of 1,3-dicarbonyl species **4** and **5**, with especially dramatic effects upon 2-fluoro-1,3-dicarbonyls **5**. The presence of additives also has clear effects on the fluorination processes of **4a** and **5a** with Selectfluor™. In order to demonstrate the quantitative applicability of our data to synthetic scenarios, both in the presence and absence of water, we performed NMR experiments (Fig. 6a–d) to monitor the kinetics of fluorination of **4a** with Selectfluor™ and compared measured data with a numerically-solved differential model (Fig. 7a–c) of the overall processes based upon the microscopic rate constants we have determined.

In MeCN-*d*_3_ alone, we reacted **4a** (30 mM) with Selectfluor™ **1** (2.1 equivalents) and the evolution of species was monitored by ^19^F NMR spectroscopy over 6 days. Without water, **5a-keto** (*δ* = –190 ppm) was formed rapidly from the large reservoir of **4a-enol** (∼90% of total **4a**), with a second kinetic phase of **5a-keto** formation associated with enolization of residual **4a-keto** (Fig. 6a and green dots in Fig. 6c). On the basis of our UV-vis kinetic data, the formation of **5a-enol** was expected to be extremely slow, with the formation of **6a** (*δ* = –103 ppm) being similarly slow as a result. This was borne out by the very slow appearance of 2,2-difluoro-1,3-dicarbonyl **6a**, with its formation only being evident at a level of ∼4% after 5 days.

The reaction conducted with 20% H_2_O in MeCN-*d*_3_ allowed for the use of higher concentrations of Selectfluor™ owing to its enhanced solubility in this medium, thus concentrations of **4a** = 60 mM and Selectfluor™ = 125 mM were used. The reaction profile showed rapid build-up of **5a-keto** (*δ* = –190 ppm) as a result of the large starting concentration of **4a-enol**. Owing to the presence of water, enolization of **5a-keto** was expected to occur more readily, and this was evidenced by the more rapid reduction in the signal for **5a-keto** (Fig. 6b and green dots in Fig. 6d) and the formation of 7.5% **6a** (*δ* = –103 ppm) after ∼3.5 h (Fig. 6d black dots), with complete conversion to **6a** being achieved over 6 days (fluorination was expected to be rapid, and this was supported by very low levels of **5a-enol** being detected in the steady state). An additional peak was present at *δ* = –111.9 ppm (Fig. 6b) which is likely to correspond to a hydrate of **6a**. The formation of a hydrate is expected, as difluoroketones are known to form stable tetrahedral adducts.^32^ We also observed this peak in our ^19^F NMR-monitored synthetic reaction to obtain an authentic sample of **6a**, however, upon work-up we did not isolate any **6a**-hydrate.

In order to further validate our kinetic model (Fig. 7a and b), the microscopic rate constants (Fig. 7c) that we have measured by UV-vis spectrophotometry were inserted into the model and numerical methods were used to solve the differential equations (Fig. 7b). The resulting predicted concentration–time profiles of all species were plotted (lines in Fig. 6c and d) to allow comparison with experimental data (dots in Fig. 6c and d).

Pleasingly, for the experiment performed in the absence of added water, the rapid evolution of **5a-keto** was modelled well by using ketonization and enolization rate constants (Table 1) for **4a** in MeCN. The addition of kinetic terms related to the auto-catalytic keto–enol tautomerism of **5a** were critical to the quantitative agreement between model and experiment for the formation of **6a**, with the formation of **6a** being predicted to reach only 1.7% after ∼5 days in the absence of this contribution, but 2.9% (*versus* ∼4% by experiment) when these terms were taken into account.

In the presence of 20% water, the build-up and break-down of **5a-keto** was modelled well alongside the profile for the formation of **6a**. We did not detect the presence of autocatalysis of the keto–enol equilibration of **5a** by our UV-vis kinetic studies, and thus did not include them in the model. However, at the higher concentrations employed in this NMR study, any such terms could become more sizeable and could contribute to improving the model.

## 3. Conclusions

We have utilized a photo-switching method for the determination of the effects of additives on keto–enol tautomerism in the 1,3-diaryl-1,3-dicarbonyls **4a–d** and the corresponding fluorinated derivatives **5a–d**. We have shown through kinetics studies that the addition of water is a simple method for increasing the rate of enolization and thus increasing the rate of formation of 2,2-difluoro-1,3-dicarbonyl **6a**. We found that small quantities of formic acid and DABCO greatly increased the enolization rate of **4a** and formic acid also increased the enolization rate of **5a**. The presence of DABCO resulted in the de-fluorination of **5a**, as evidenced by NMR studies, whereas **5b** was not de-fluorinated by DABCO. The non-fluorinated product of Selectfluor™, ClCH_2_-DABCO^+^BF_4_^–^, which is rarely considered in synthetic application, had small but detectable effects on keto–enol equilibration kinetics, however, the nature of the effects is not clear.

We also obtained kinetic data on the fluorination of mono-fluoroenols **5a–d** with Selectfluor™ **1** and NFSI **2** under a variety of conditions. We have shown that the addition of a second fluorine atom occurs at a rate greater than or similar to that of the addition of the first fluorine atom. The rate-limiting step in the overall difluorination mechanism is therefore the enolization of the mono-fluoroketo tautomer, represented by *k*_for_(F) in Fig. 7a.

Our kinetics studies correlate very well with previous synthetic studies: Banks *et al.*^19^ first reported the selective monofluorination of **4a** using Selectfluor™ in MeCN, which gave 100% crude and 84% pure yields. We also observed complete conversion of **4a** to **5a** by both spectrophotometric and NMR methods, due to the high enol content of **4a**. Yi and co-workers^64^,^65^ reacted a series of aromatic 1,3-dicarbonyl compounds with 2.1 equivalents of Selectfluor™ in 10 : 1 MeCN/H_2_O at 25 °C for 1–2 days, to obtain a range of 2,2-difluoro-1,3-diketones in approx. 90% yield. This matches our conclusion that water must be present to facilitate the enolization of **5a-keto** and thus allow difluorination to occur within reasonable timescales. Pattison *et al.*^66^ attempted the difluorination of an aromatic β-ketoester with Selectfluor™ (2.5 equivalents) under reflux conditions in MeCN, which gave an 8 : 1 ratio of mono- and difluorinated products. This was attributed to the lower enol content of β-ketoesters compared to **4a**.^66^ Since water was not used in the reaction, the enolization of the β-ketoester was presumably slow, which explains the low conversion to the difluorinated product. Stavber *et al.*^28^ reported monofluorinations of cyclic 1,3-diketones and β-ketoesters in water using Selectfluor™ (1.1 equivalents), obtaining yields of 74–91%. The difluorinations of acyclic 1,3-diketones and β-ketoesters *via* Selectfluor™ (2.2 equivalents) in water gave yields of 78–89%. All reactions were conducted at 70 °C for 4–10 h. Fluorination of the acyclic 1,3-dicarbonyls could not be selectively stopped at the monofluorination stage, but by using 2.2 equivalents of Selectfluor™ the 2,2-difluoro-1,3-dicarbonyls were obtained without additional activation of the starting material. Syntheses of α,α-difluoro-β-ketoamides have been achieved using H_2_O:PEG-400 solvent mixtures in the presence of K_2_CO_3_,^67^ as well as very recently reported H_2_O:MeCN systems^68^ in green chemistry research programs, for which our experiments provide supporting mechanistic evidence of the crucial roles of water and base.

Our studies give direct evidence that water plays an essential role in accelerating the enolization of mono-fluorodiketone derivatives to allow the formation of difluorodiketones. Our findings have important implications for synthetic fluorination procedures: the addition of small quantities of water to partially enolic 1,3-dicarbonyl derivatives increases rates of keto to enol tautomerism, supporting the formation of the key enol intermediates required for both the first and second fluorination steps. Furthermore, water also enhances the rate of fluorination of fluoroenols, again supporting the expedited formation of pharmaceutically relevant α,α-difluoroketonic compounds.

## 4. Methods

The ESI contains details of methods, kinetics experiments and product analyses.

## Conflicts of interest

The authors declare no conflict of interest.

## Supplementary Material

Supplementary information
