# Supplementary material for: Enolization rates control mono- versus di-fluorination of 1,3-dicarbonyl derivatives
Source: Chem Sci. 2019 Sep 16;10(44):10318–30. doi: 10.1039/c9sc04185k (PMC6979503; doi:10.1039/c9sc04185k)
Supplement: Supplementary file 1 [file SC-010-C9SC04185K-s001.pdf]

Electronic Supplementary Material (ESI) for Chemical Science.
This journal is © The Royal Society of Chemistry 2019

Enolization rates control mono- *versus* di-fluorination of 1,3-dicarbonyl derivatives

Neshat Rozatian[a], Andrew Beeby[a], Ian W. Ashworth[b], Graham Sandford[a], David R. W. Hodgson[a]*

a) **Chemistry Department, Durham University, South Road, Durham, UK, DH1 3LE**

b) **AstraZeneca, Pharmaceutical Technology & Development, Macclesfield, UK, SK10 2NA**

SUPPORTING INFORMATION

Table of Contents

1. General Instrumentation and Materials 5

2. Experimental 5

2.1 Synthesis of bisphenylsulfonylimide sodium salt $(PhSO_2)_2N^- Na^+$ 5

2.2 Synthesis of 2,2-difluoro-1,3-diphenyl-1,3-propanedione (6a) 6

2.3 Confirmation of purities of compounds 5a-d 8

3. Kinetics Studies Conducted by UV-Vis Spectrophotometry 12

3.1 Methods 12

3.1.1 Photoketonization and relaxation experiments 12

3.1.2 Reference UV-vis spectra for compounds 4a-d and 5a-d 13

3.2 Keto:enol ratios in the presence of additives determined by NMR spectroscopy 14

3.2.1 De-fluorination of 5a 15

3.3 Photoketonization spectra 16

3.4 Kinetics of relaxation of 4a 17

3.4.1 In the absence of additives 17

3.4.2 With water as the additive 17

3.4.3 With formic acid as the additive 18

3.4.4 With DABCO as the additive 18

3.4.5 With $ClCH_2$–DABCO tetrafluoroborate as the additive 19

3.4.6 With water and 0.0125 mM $ClCH_2$–DABCO tetrafluoroborate as the additives 20

3.4.7 With $LiBF_4$ as the additive 21

3.4.8 With $(PhSO_2)_2NH$ as the additive 22

3.4.9 With $(PhSO_2)_2N^- Na^+$ as the additive 23

3.4.10 With $Bu_4N^+ BF_4^-$ as the additive 23

3.5 Kinetics of relaxation of 4b-d without additives and Hammett correlation 24

3.6 Kinetics of relaxation of 4b in the presence of additives 25

3.6.1 Additives: formic acid, DABCO 25

3.6.2 Additives: water, $ClCH_2$–DABCO tetrafluoroborate 25

3.7 Kinetics of relaxation of 4c in the presence of additives 26

3.7.1 Additives: water, DABCO 26

3.8 Kinetics of relaxation of 4d in the presence of additives 27

3.8.1 Additives: water, DABCO, $ClCH_2$-$DABCO^+ BF_4^-$ 27

3.9 Kinetics of relaxation of 5a 28

3.9.1 In the absence of additives 28

3.9.2 With water as the additive ... 29
3.9.3 With formic acid as the additive ... 30
3.9.4 With DABCO as the additive ... 30
3.9.5 With water and 0.0125 mM $ClCH_2$-$DABCO^+$ BF_4^- ... 31
3.9.6 With $ClCH_2$-$DABCO^+$ BF_4^- as the additive ... 32
3.9.7 With Bu_4N^+ BF_4^- as the additive ... 32
3.10 Kinetics of relaxation of 5b ... 33
3.10.1 In the absence of additives ... 33
3.10.2 With water as the additive ... 34
3.10.3 Other additives: formic acid, DABCO ... 34
3.11 Kinetics of relaxation of 5c ... 35
3.11.1 In the absence of additives ... 35
3.11.2 With water as the additive ... 35
3.12 Kinetics of relaxation of 5d ... 36
3.12.1 In the absence of additives ... 36
3.12.2 With water as the additive ... 37
3.13 Kinetics of fluorination of 5a-d by Selectfluor™ ... 38
3.13.1 Fluorination of 5a ... 38
3.13.2 Fluorination of 5b ... 42
3.13.3 Fluorination of 5c ... 47
3.13.4 Fluorination of 5d ... 48
3.13.5 Hammett and Eyring correlations ... 49
3.14 Kinetics of fluorination of 5a-d by NFSI ... 50
3.14.1 Fluorination of 5a ... 50
3.14.2 Fluorination of 5b ... 51
3.14.3 Fluorination of 5d ... 52
3.14.4 Hammett correlations ... 53
3.15 Kinetics of fluorination of 5a-enol by Selectfluor™ with water ... 54
3.15.1 With 20% water in MeCN ... 54
3.15.2 With 20% water in MeCN: linear analysis ... 55
3.16 Kinetics of fluorination of 5a-enol by Selectfluor™ with formic acid ... 56
3.16.1 With 3% formic acid in MeCN ... 56
3.16.2 With 5% formic acid in MeCN ... 57
3.16.3 With 20% formic acid in MeCN ... 58

3.17 Kinetics of fluorination of 5a-enol by Selectfluor™ with $Bu_4N^+ BF_4^-$ 59
3.18 Kinetics of fluorination of 4a-enol by Selectfluor™ in H_2O/MeCN mixtures 60
3.18.1 With 20% water in MeCN.. 60
3.18.2 With 20% water in MeCN: linear analysis 61
4. Difluorination of 4a-enol *via* Selectfluor™, 20% water in MeCN-d_3.. 62
5. References 64

1. General Instrumentation and Materials

^1H NMR (400 MHz), ^{13}C NMR (101 MHz) and ^{19}F NMR (376 MHz) were measured on a Bruker-Avance 400 MHz spectrometer. LC-MS data were obtained using a triple quadrupole (TQD) mass spectrometer equipped with an Acquity UPLC (Waters Ltd, UK), EH C18 column (1.7 µm, 2.1 mm × 50 mm) and a photodiode array detector. Conditions for LC resolution were as follows: buffer A = water, 0.1% formic acid; buffer B = MeCN. Elution conditions: flow rate = 0.6 mL/min; 0-0.2 min isocratic 95% A, 5% B; 0.2-4 min linear gradient to 5% A, 95% B; 4-4.5 min isocratic 5% A, 95% B; 4.5-5 min linear gradient to 95% A, 5% B. Chemicals were purchased from Fluorochem or Sigma Aldrich and, unless otherwise stated, used without purification. NMR solvents were purchased from Cambridge Isotopes Inc., supplied by Goss Scientific and Sigma-Aldrich. These chemicals were used without further purification and stored under appropriate conditions, as detailed in the manufacturer's instructions. Organic solvents were used without further purification. Selectfluor™ and NFSI were purchased from Fluorochem. HPLC grade acetonitrile and formic acid (Romil SpR Super Purity Reagent) were used.

2. Experimental

Compound **4a** was purchased from Sigma Aldrich and was recrystallized (hexane) and dried under vacuum before use in kinetic measurements. The 1,3-diaryl-1,3-propanediones **4b-d** were synthesised according to literature procedures[1] and recrystallized from hexane/ethyl acetate before use in kinetics experiments. The 2-fluoro-1,3-diaryl-1,3-propanediones **5a-d** were synthesised according to literature procedures[2] and recrystallized from chloroform/hexane before use in kinetics experiments. $ClCH_2$-DABCO tetrafluoroborate was synthesised using a modified version of the literature procedure.[3]

2.1 Synthesis of bisphenylsulfonylimide sodium salt $(PhSO_2)_2N^- Na^+$

$(PhSO_2)_2NH$ (0.50 g, 1.7 mmol) was dissolved in MeOH (5 mL). NaOMe (0.09 g, 1.7 mmol) was added and the mixture was stirred at RT for 2 h. Evaporation of the solvent gave the product as a white solid (0.52g, 97%). **^{1}H NMR** (400 MHz, DMSO-d_6) δ = 7.70-7.64 (m, 4H), 7.46-7.34 (m, 6H). **^{13}C NMR** (101 MHz, DMSO-d_6) δ = 145.8, 130.4, 128.0, 126.2. These assignments are in agreement with the literature.[4]

2.2 Synthesis of 2,2-difluoro-1,3-diphenyl-1,3-propanedione (6a)

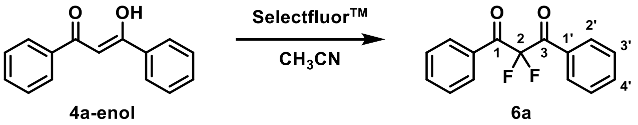

1,3-diphenyl-1,3-propanedione (300 mg, 1.34 mmol) was dissolved in MeCN (8 mL) and deionised H_2O (2 mL), and Selectfluor™ (995 mg, 2.81 mmol) was added. The reaction mixture was stirred at room temperature for 3 days and aliquots from the mixture (0.7 mL) were directly monitored by ^{19}F NMR spectroscopy using a D_2O lock tube (aliquots were returned to the reaction mixture following analysis). The solvent was evaporated *in vacuo*, and the white residue was dissolved in ethyl acetate (20 mL) and washed with water (3 × 20 mL) and brine (20 mL). The organic phase was separated, dried ($MgSO_4$), solvent evaporated *in vacuo* and 2,2-difluoro-1,3-diphenyl-1,3-propanedione was obtained as colourless crystals (329 mg, 94%). Further purification was not required. **IR** ν_{max}/ cm^{-1} 3072, 1695, 1594, 1449, 1251, 1136, 940, 887, 771, 720, 679, 664, 570, 523. **^{1}H NMR** (400 MHz, CD_3CN) δ = 8.05 (4H, ddt, J_{HH} = 7.8, 2.3 Hz, $^5J_{HF}$ = 1.1 Hz, 2'-*H*), 7.79-7.71 (2H, m, 4'-*H*), 7.63-7.53 (4H, m, 3'-*H*). **^{13}C NMR** (101 MHz, CD_3CN) δ = 187.5 (t, $^2J_{CF}$ = 26.8 Hz, C1, C3), 135.5 (s, C_{arom}), 131.4 (d, J_{CF} = 1.6 Hz, C_{arom}), 129.9 (t, J_{CF} = 2.6 Hz, C_{arom}), 129.2 (s, C_{arom}), 112.5 (t, $^1J_{CF}$ = 265.4 Hz, C2). **^{19}F NMR** (376 MHz, CD_3CN) δ = −103.4 (p, $^5J_{FH}$ = 1.1 Hz). **ESI-MS** (ES^+, R_t 2.965) m/z 261.211 $[M+H]^+$. These assignments are in agreement with the literature.[5]

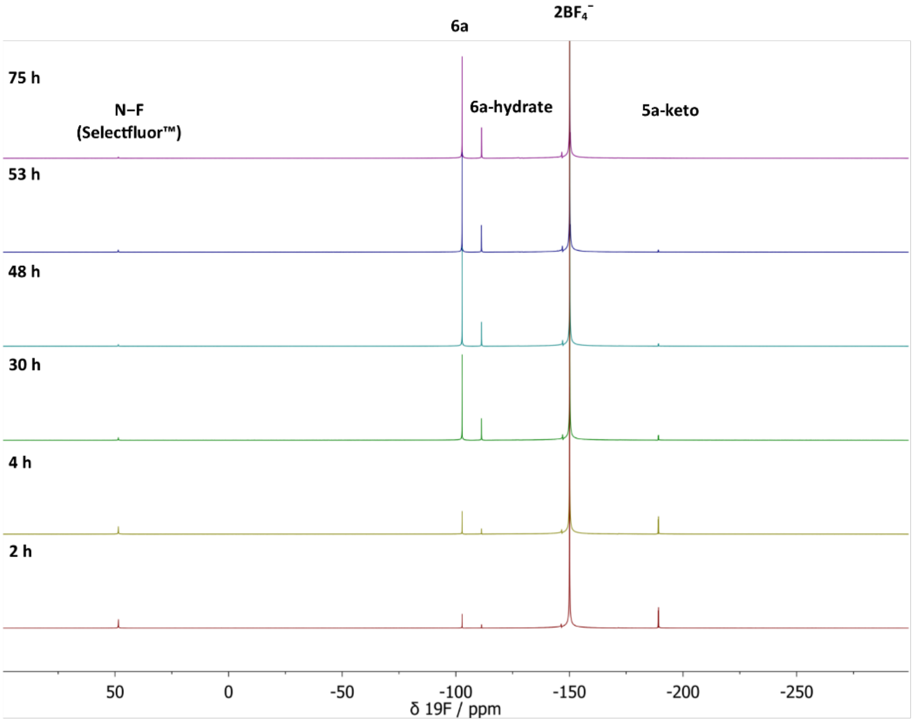

Figure 1: The reaction of **4a** to form **6a** was monitored by ^{19}F NMR over 3 days using a non-quantitative wide-sweep method to allow the disappearance of Selectfluor™ to be monitored alongside the evolution of product species.

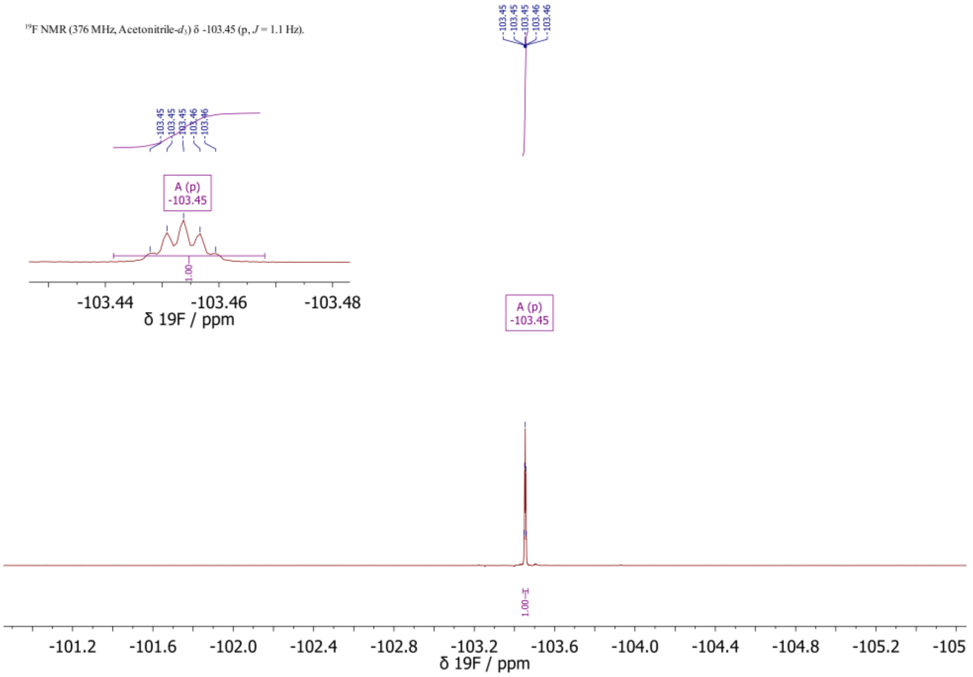

Figure 2: ^{19}F NMR spectrum of 2,2-difluoro-1,3-diphenyl-1,3-propanedione **6a** in CD_3CN.

To confirm that the additional peak at δ = −111.9 ppm observed in the ^{19}F NMR spectra for reactions conducted in water/CD_3CN mixtures was a hydrate of **6a**, we obtained an NMR spectrum of an authentic sample of **6a** in 20% water in CD_3CN (v/v). The peak at δ = −111.9 ppm was present (Figure 3). This hydrate was not isolated after work-up in synthetic reactions.

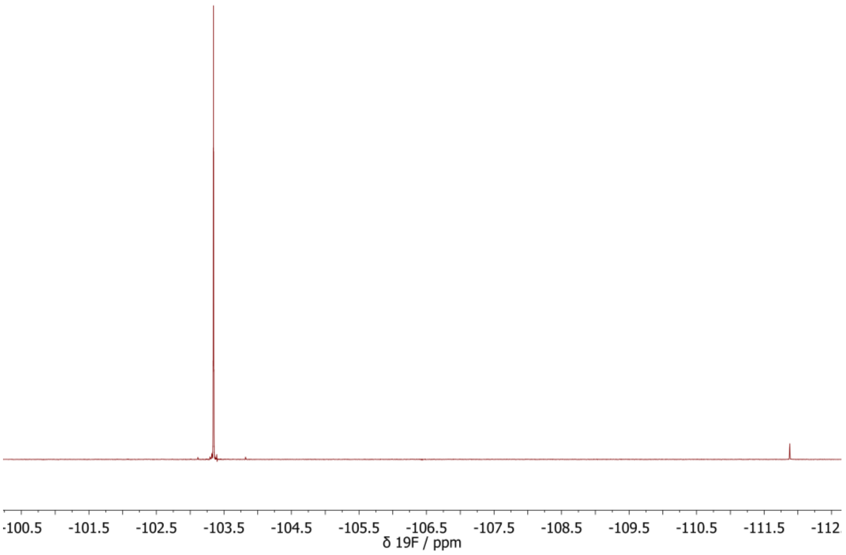

Figure 3: ^{19}F NMR spectrum of 2,2-difluoro-1,3-diphenyl-1,3-propanedione in 20% H_2O in CD_3CN. Peaks at δ = −103.4 ppm and δ = −111.9 ppm correspond to **6a** and **6a**-hydrate, respectively.

2.3 Confirmation of purities of compounds 5a-d

All kinetic studies of keto-enol relaxation and fluorination processes were performed by monitoring the appearance or disappearance of keto and enol species by UV-vis spectrophotometry. Given that compounds **5a-d** were prepared from **4a-d**, we were concerned that small amounts of residual **4a-d** in our preparations of **5a-d** could interfere with our kinetic studies. In order to confirm the spectrophotometric purities of **5a-d**, NMR analyses were supplemented by LC-MS with diode array detection. The chromatograms were viewed at or near the λ_{max} values of the enol forms of **4a-d** and **5a-d**. All chromatograms show only the keto and enol forms of the mono-fluorinated systems **5a-d** and no evidence of un-fluorinated systems **4a-d**. NMR spectra for **5a-d** may be found in the ESI of our previous publication.[2]

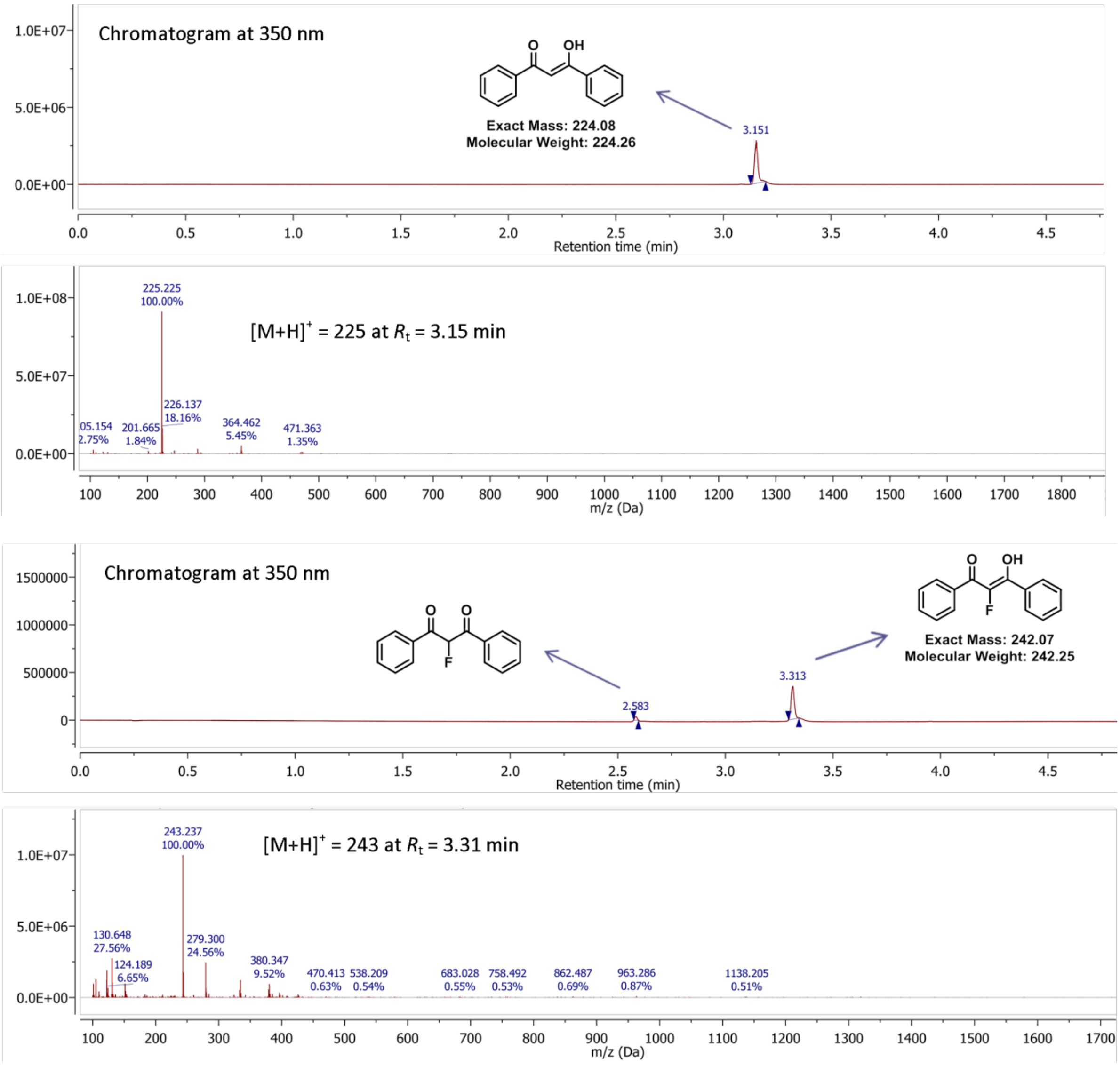

Figure 4: LC-MS spectra and single wavelength diode array chromatograms corresponding to authentic samples of **4a** (top) and **5a** (bottom).

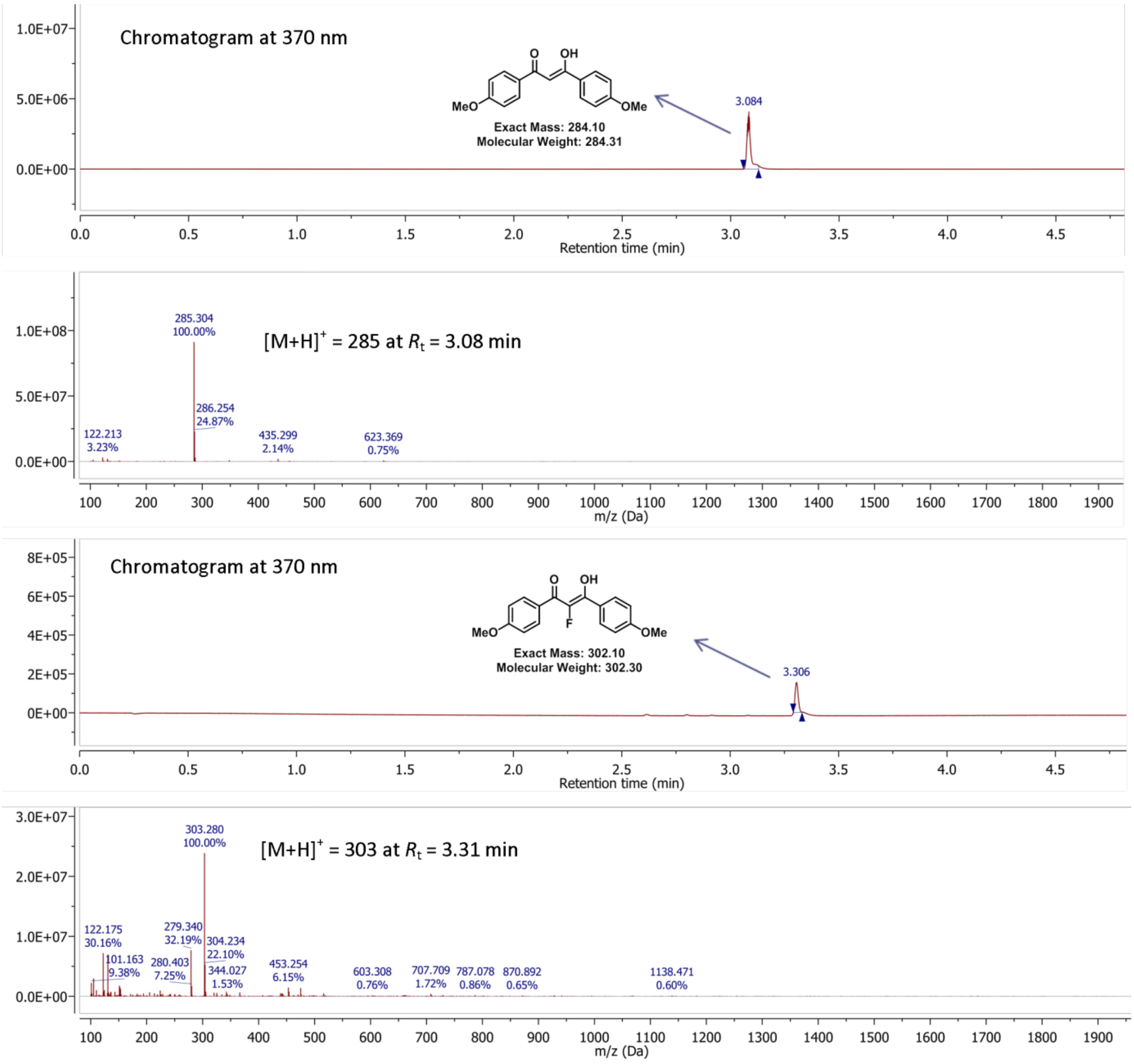

Figure 5: LC-MS spectra and single wavelength diode array chromatograms corresponding to authentic samples of **4b** (top) and **5b** (bottom).

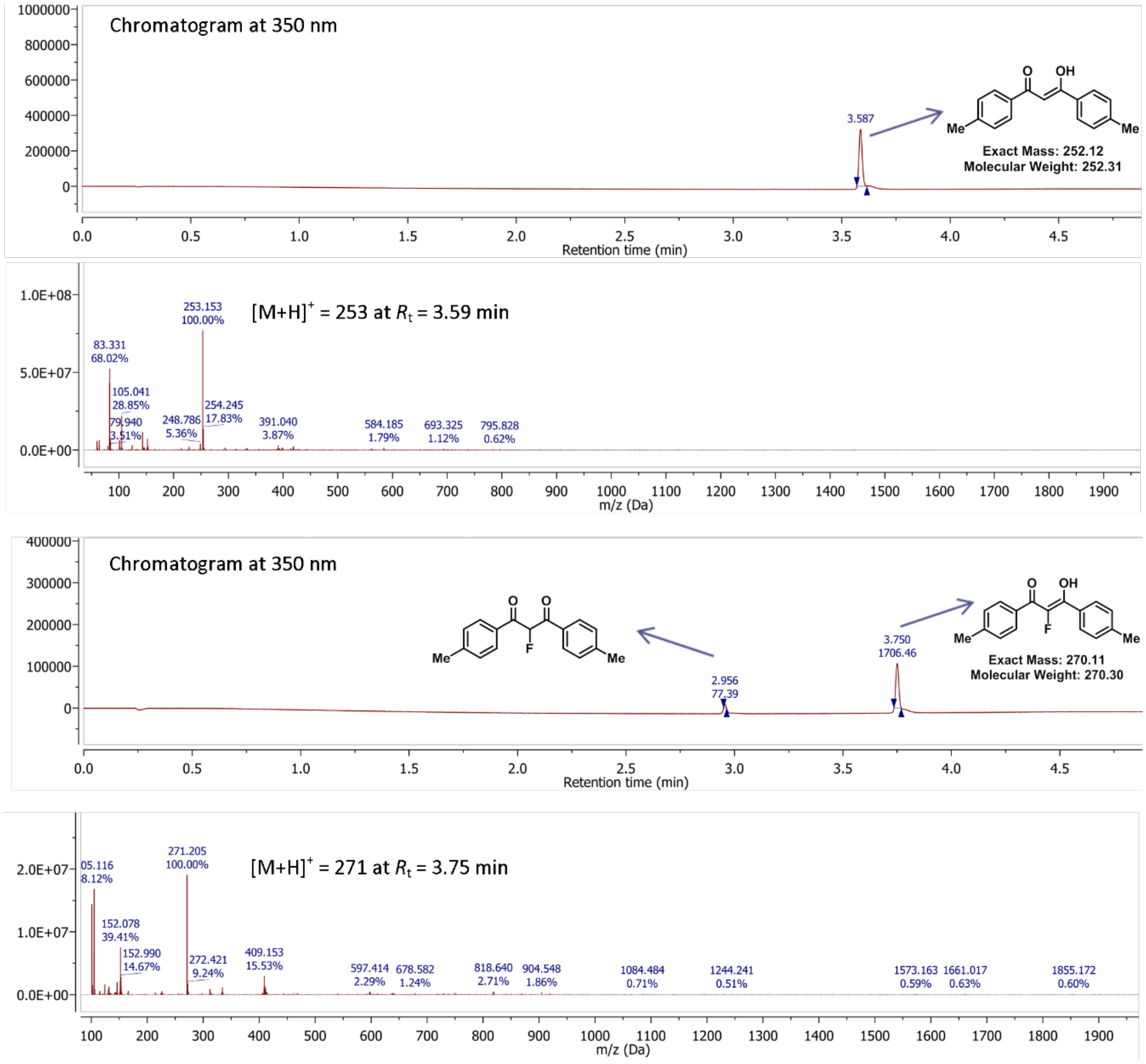

Figure 6: LC-MS spectra and single wavelength diode array chromatograms corresponding to authentic samples of **4c** (top) and **5c** (bottom).

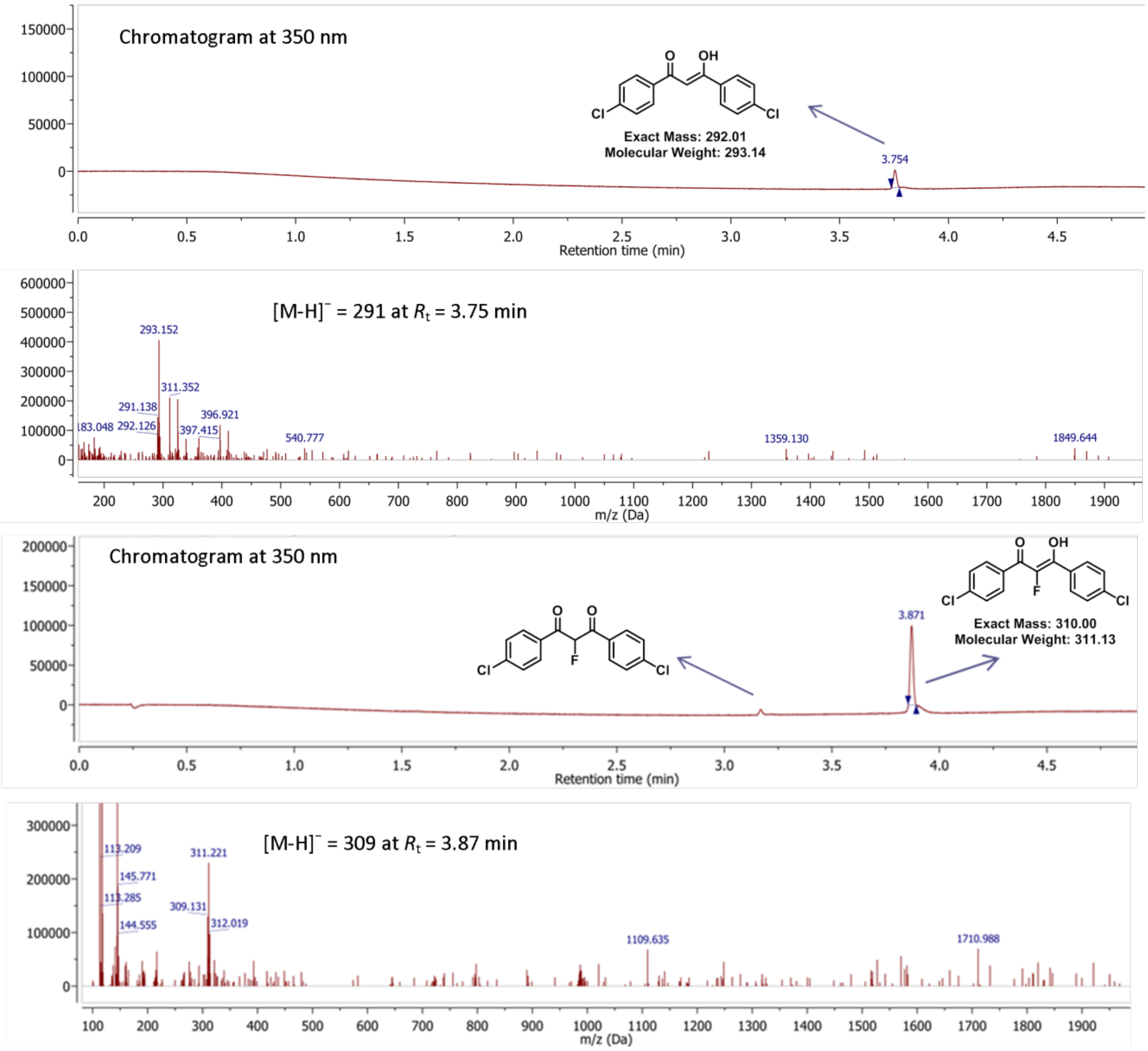

Figure 7: LC-MS spectra and single wavelength diode array chromatograms corresponding to authentic samples of **4d** (top) and **5d** (bottom).

3. Kinetics Studies Conducted by UV-Vis Spectrophotometry

3.1 Methods

Kinetics studies were carried out using a Varian Cary-100 Bio UV-vis Spectrophotometer equipped with a Cary Temperature Controller unit, or a Varian Cary-50 Bio UV-vis Spectrophotometer connected to a Varian Cary PCB-150 Water Peltier system. Samples were contained in quartz absorption cuvettes with a path length of 1 cm. All spectra were zeroed against air. Reactions were followed by monitoring the disappearance of the enol at a fixed wavelength corresponding to the maximum absorbance (λ_{max}) of the relevant enol (Table 1). All fluorination reactions were carried out in the presence of excess N−F reagent in order to attempt to maintain pseudo-first-order behaviours. Error values quoted in Section 3.13 and 3.14 are standard error values obtained from data fitting in KaleidaGraph software. Stock solutions of **4a-d** (5-10 mM), **5a-d** (2-20 mM), Selectfluor™ (40 mM), NFSI (200 mM), DABCO (10 mM) and $ClCH_2$-DABCO$^+$ BF_4^- (5 mM) were prepared in volumetric flasks in MeCN (HPLC grade), except where stated otherwise. Aliquots of the required stock solutions were removed and diluted to the desired concentrations in cuvettes, which were placed in the spectrophotometer for 10 min to equilibrate to the required temperature. Kinetics studies were carried out using the “Scanning Kinetics” or “Single Wavelength Kinetics” programs.

3.1.1 Photoketonization and relaxation experiments

Solutions of **4a-d** and **5a-d** were prepared at the required concentration in quartz cuvettes, equipped with stirrer bars. The stirred solutions were irradiated with a 365 nm UV lamp for 3 h, at room temperature. The UV lamp was then removed, and if required, the additive was transferred to the cuvette. Time arrayed multi-wavelength scans were acquired every 15 min, unless stated otherwise, to avoid continuous irradiation of the cuvette at smaller time intervals, which would slow down the rate of relaxation. Time-arrayed single-wavelength scans were conducted, as required.

3.1.2 Reference UV-vis spectra for compounds 4a-d and 5a-d

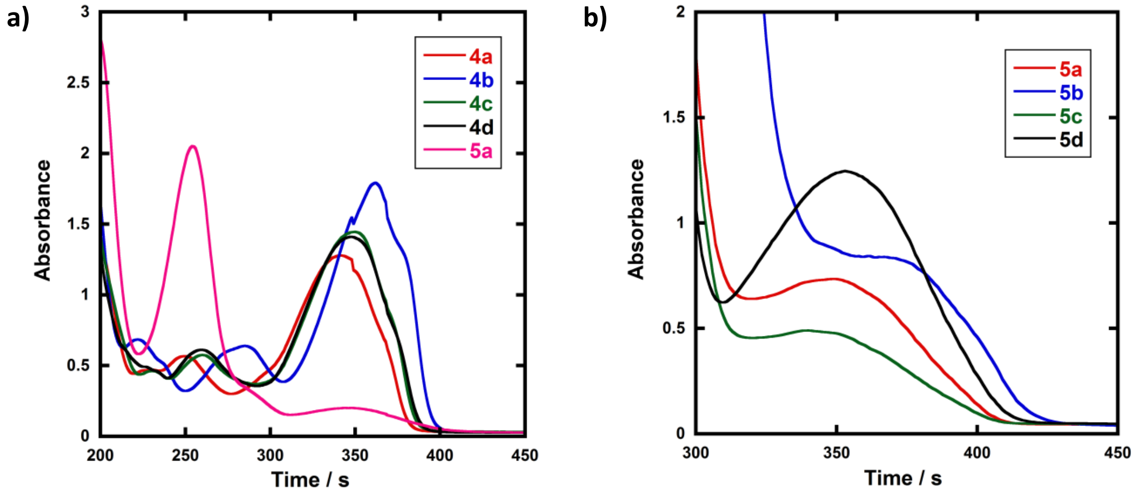

Figure 8: UV-vis spectra of authentic samples of compounds **4a-d** and **5a-d**. Concentrations of solutions: **4a-d** are 0.05 mM; **5a**, **5b** and **5d** are 0.5 mM; **5c** is 0.4 mM.

Table 1: λ_{max} values for the enol tautomers of compounds **4a-d** and **5a-d**, in MeCN.

Compound	λ_{max} (enol) / nm
**4a**	341
**4b**	362
**4c**	350
**4d**	347
**5a**	350
**5b**	370
**5c**	340
**5d**	353

3.2 Keto:enol ratios in the presence of additives determined by NMR spectroscopy

The keto:enol ratios in CD_3CN were obtained using ^1H NMR spectroscopy in the case of compounds **4a-d** and ^{19}F NMR spectroscopy for compounds **5a-d**. In order to obtain quantitative integral values, relaxation delays of 20 s and 8 s was employed for ^1H NMR and ^{19}F NMR experiments, respectively. The concentrations of **4a-d** and **5a-d** were 25 mM for each NMR experiment. Unless otherwise stated, the solutions were allowed to equilibrate for 10 half-lives before NMR spectra were acquired. Ratios were determined using keto and enol peak integrals. For example, with **4a**, peaks corresponding to the enol form (δ = 7.08 ppm) and the keto form (δ = 4.72 ppm) were integrated across a 0.05 ppm range.

Table 2: Keto:enol ratios of **4a-d** and **5a-d** in CD_3CN.

Compound	Additive	Quantity of additive in CD_3CN	Keto:enol ratio
**4a**	None	-	9:91
	H_2O	20%	13:87
	H_2O	50%	15:85
	Formic acid	1%	9:91
	Formic acid	2%	9:91
	Formic acid	3%	10:90
	DABCO	25 mM (1 eq)	13:87
	DABCO	50 mM (2 eq)	13:87
	$ClCH_2$-$DABCO^+$ BF_4^-	25 mM (1 eq)	10:90
	Bu_4N^+ BF_4^-	240 mM	10:90
**4b**	None	-	20:80
	H_2O	10%	21:79
	Formic acid	2%	16:84
	DABCO	2.5 mM (0.1 eq)	16:84
	$ClCH_2$-$DABCO^+$ BF_4^-	25 mM (1 eq)	17:83
**4c**	None	-	11:89
**4d**	None	-	7:93
**5a**	None	-	95:5
	D_2O	50%	95:5
	Formic acid	2%	95:5
	DABCO	25 mM (1 eq)	Loss of F
	H_2O	20%	96:4
	Bu_4N^+ BF_4^-	300 mM	96:4
	H_2O, Bu_4N^+ BF_4^-	20%, 250 mM	96:4
**5b**	None	-	98:2
	D_2O	50%	95:5
	Formic acid	2%	97:3
	DABCO	25 mM (1 eq)	98:2
**5c**	None	-	87:13
**5d**	None	-	92:8

3.2.1 De-fluorination of 5a

The NMR spectra below (Figure 9) correspond to the mixture of **5a** (25 mM) and DABCO (25 mM) after an incubation time of ~30 min. In the ^{1}H NMR spectrum, the peak at δ = 6.93 ppm which corresponds to the fluoroketo tautomer has almost disappeared. In the ^{19}F NMR spectrum, peaks at δ = −189.8 ppm (fluoroketo) and δ = −169.5 ppm (fluoroenol) have also disappeared and a new peak at δ = +16.5 ppm is present, which may indicate the formation of an N−F species. Other smaller peaks have also appeared between −90 ppm and −150 ppm.

a)

b)

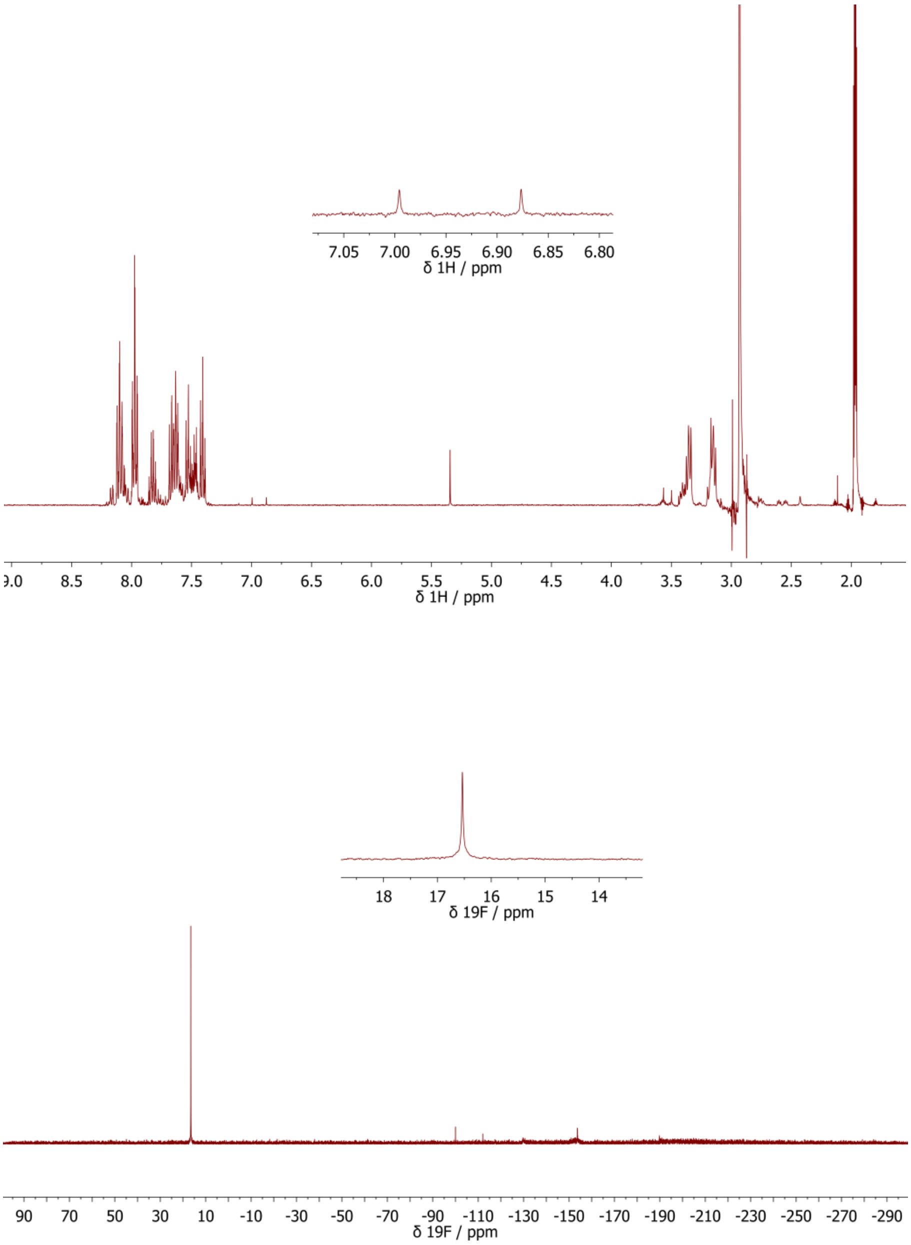

Figure 9: (a) ^{1}H NMR spectrum of the mixture of **5a** and DABCO. (b) ^{19}F NMR spectrum of the same sample.

3.3 Photoketonization spectra

The photoketonizations of solutions of **4a-d** and **5a-d** were carried out using the method discussed in Section 3.1.1. We discontinuously monitored the progress of the photoketonizations by acquiring UV-vis spectra at various time intervals, shown below.

Spectra for photoketonization of **4a**:

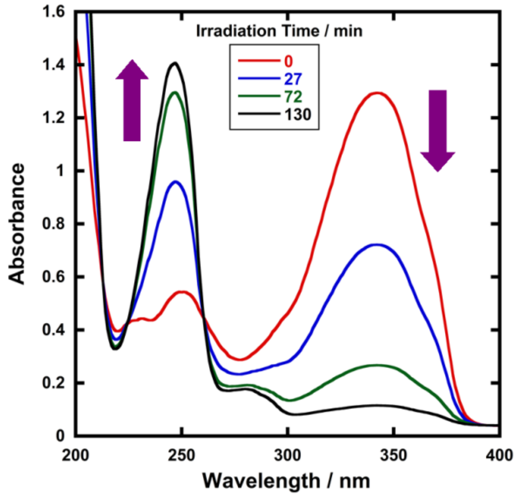

Figure 10: Spectra corresponding to photoketonization of **4a** (0.05 mM) over time, with decrease at λ_{max} (enol) = 341 nm and increase in absorbance at λ_{max} (keto) = 250 nm.

Spectra for photoketonization of **5a**:

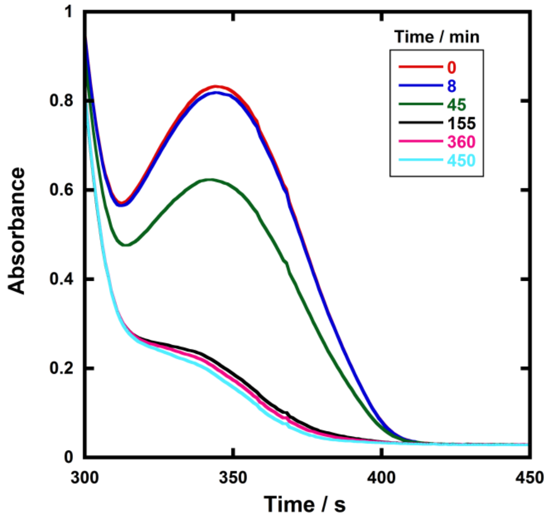

Figure 11: Spectra corresponding to photoketonization of **5a** (0.5 mM) over time, with decrease at λ_{max} (enol) = 350 nm. Absorbances below 300 nm were saturated due to the high concentration of **5a-keto** and are therefore not shown.

3.4 Kinetics of relaxation of 4a

3.4.1 In the absence of additives

a)

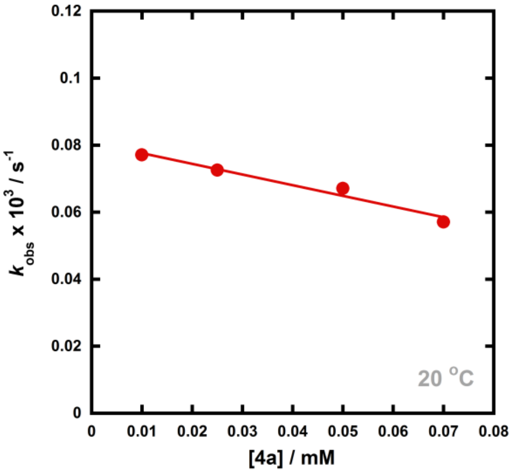

b)

[4a] / mM	k_{obs} (relaxation) / s^{-1}
0.01	7.71×10^{-5}
0.025	7.26×10^{-5}
0.05	6.71×10^{-5}
0.07	5.71×10^{-5}

Figure 12: (a) k_{obs} for relaxation correlated against the concentration of **4a**. (b) k_{obs} values obtained with different concentrations of **4a**, at 20 °C.

3.4.2 With water as the additive

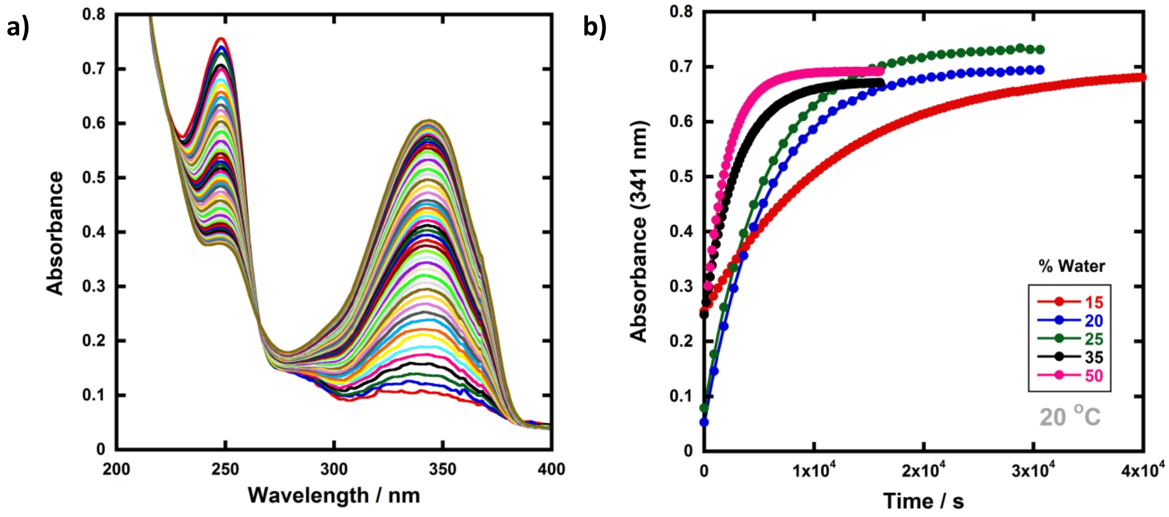

Figure 13: (a) The relaxation of **4a-keto** (0.025 mM) with 50% v/v deionised water in MeCN. The scans were acquired every 40 s for 1 h. (b) The relaxation of **4a-keto** (0.025 mM) in the presence of deionised water (v/v 15%, 20%, 25%, 35%, 50%), monitored at λ_{max} = 341 nm at 20 °C. The k_{obs} values obtained in each experiment are reported in Table 1 of the main text.

3.4.3 With formic acid as the additive

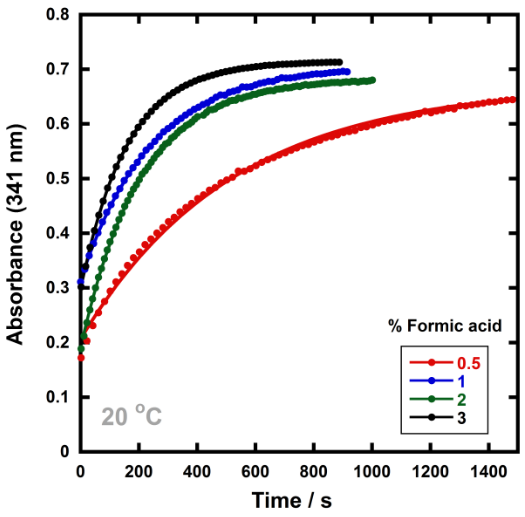

Figure 14: The relaxation of **4a-keto** (0.025 mM) in the presence of formic acid (v/v 0.5%, 1%, 2%, 3%), monitored at λ_{max} = 341 nm in MeCN at 20 °C. The k_{obs} values obtained in each experiment are reported in Table 1 of the main text.

3.4.4 With DABCO as the additive

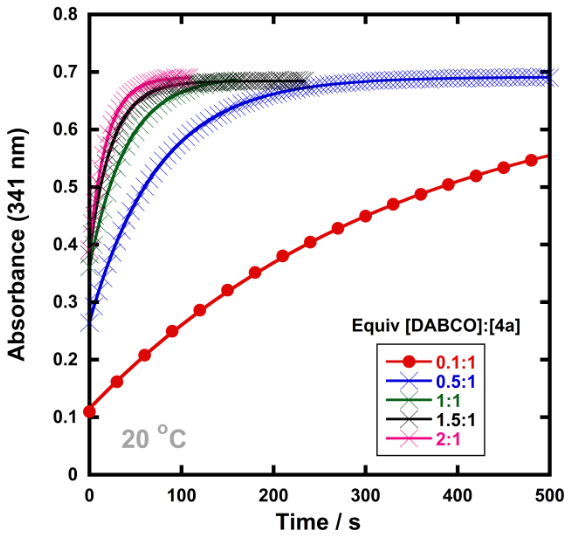

Figure 15: The relaxation of **4a-keto** (0.025 mM) in the presence of different concentrations of DABCO, monitored at λ_{max} = 341 nm in MeCN at 20 °C. The k_{obs} values obtained in each experiment are reported in Table 1 of the main text.

3.4.5 With $ClCH_2$–DABCO tetrafluoroborate as the additive

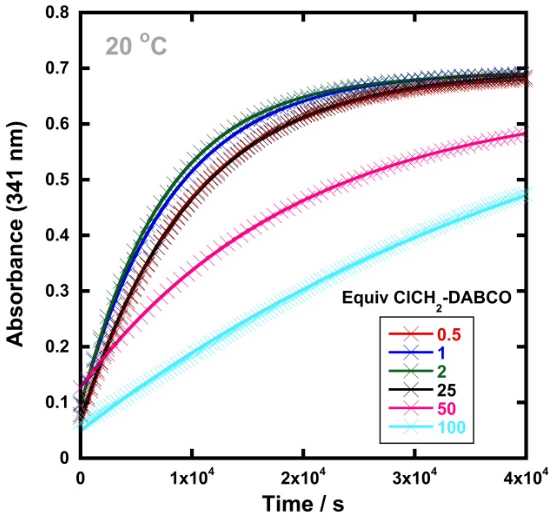

Figure 16: The relaxation of **4a-keto** (0.025 mM) in the presence of different concentrations of $ClCH_2$–DABCO$^+$ BF_4^-, monitored at λ_{max} = 341 nm in MeCN at 20 °C. The k_{obs} values obtained in each experiment are reported in Table 1 of the main text. The trends in k_{obs} values are shown in Figure 17.

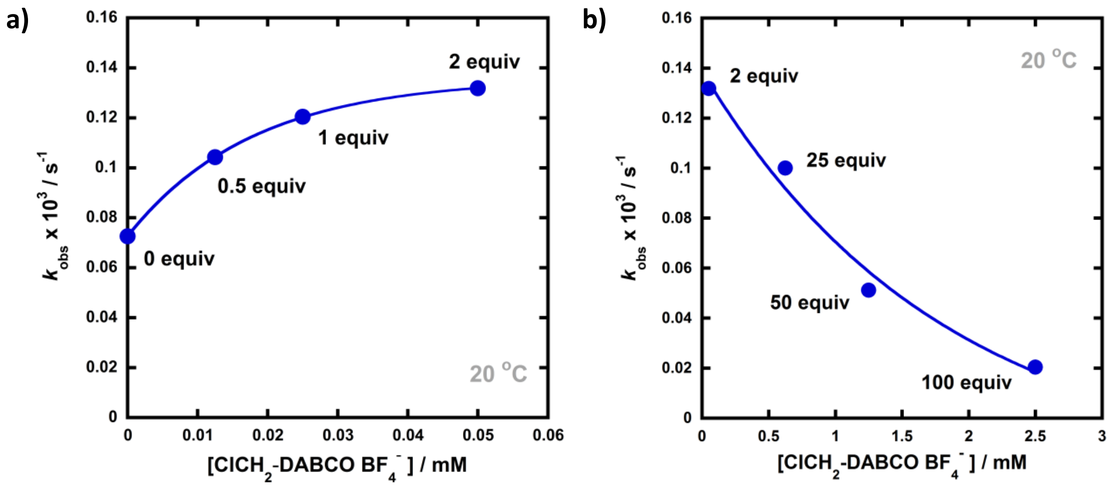

Figure 17: Trends observed in the rates of relaxation (k_{obs}) of **4a** (0.025 mM) upon addition of different quantities of $ClCH_2$–DABCO$^+$ BF_4^-, in MeCN at 20 °C: (a) 0.5–2 equivalents (b) 25–100 equivalents.

3.4.6 With water and 0.0125 mM $ClCH_2$–DABCO tetrafluoroborate as the additives

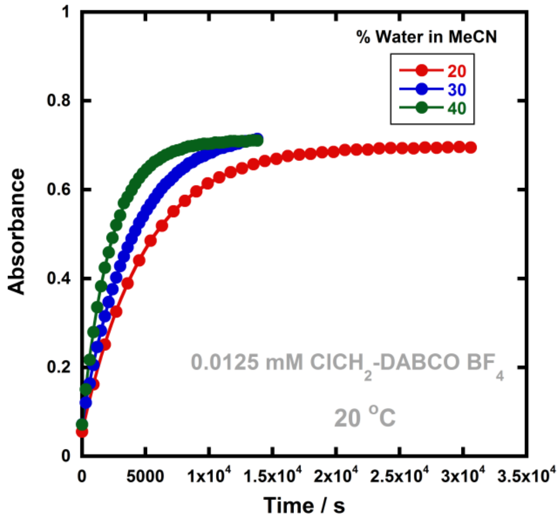

Figure 18: The relaxation of **4a-keto** (0.025 mM) in the presence of different quantities of water with 0.0125 mM $ClCH_2$–DABCO$^+$ BF_4^- monitored at λ_{max} = 341 nm in MeCN at 20 °C.

Table 3: k_{obs} values for the relaxation of **4a-keto** (0.025 mM) in the presence of 0.0125 mM $ClCH_2$–DABCO$^+$ BF_4^-, with different percentages of water in MeCN.

Water / % in MeCN	k_{obs} (relaxation) / s^{-1}	$t_{1/2}$ / min
20	2.05×10^{-4}	56
30	2.58×10^{-4}	48
40	4.47×10^{-4}	26

3.4.7 With $LiBF_4$ as the additive

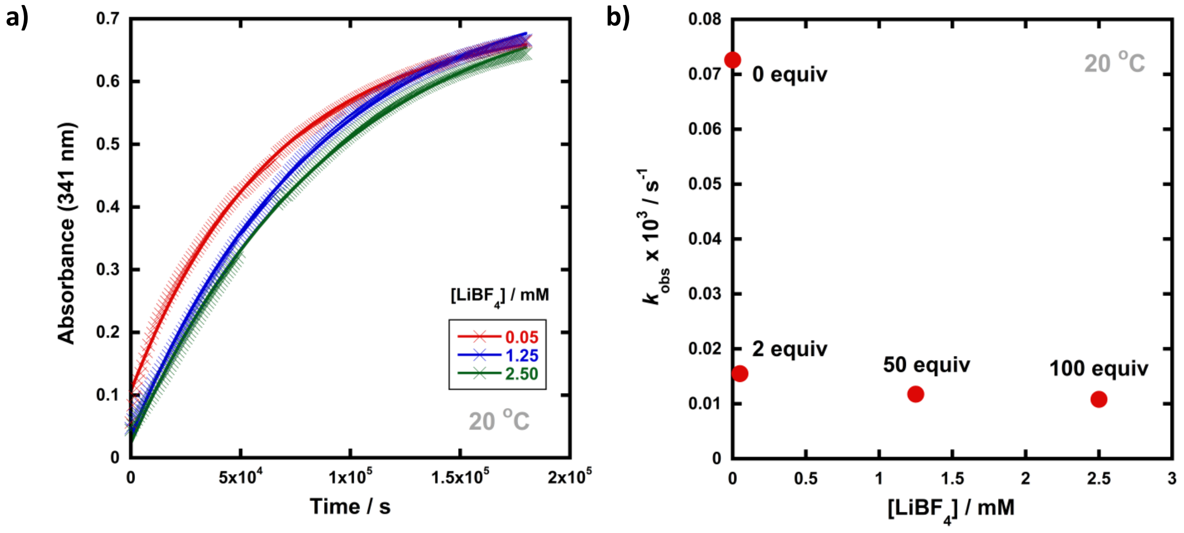

Figure 19: (a) The relaxation of **4a-keto** (0.025 mM) in the presence of different concentrations of $LiBF_4$, monitored at λ_{max} = 341 nm in MeCN at 20 °C. (b) Trend observed in the rates of relaxation (k_{obs}) of **4a-keto** (0.025 mM) upon addition of different quantities of $LiBF_4$, in MeCN at 20 °C.

Table 4: k_{obs} values for the relaxation of **4a-keto** (0.025 mM) in the presence of different concentrations of $LiBF_4$ in MeCN.

**[$LiBF_4$] / mM**	**k_{obs} (relaxation) / s^{-1}**	**$t_{1/2}$ / min**
0.05	1.55×10^{-5}	745
1.25	1.18×10^{-5}	979
2.50	1.08×10^{-5}	1070

3.4.8 With $(PhSO_2)_2NH$ as the additive

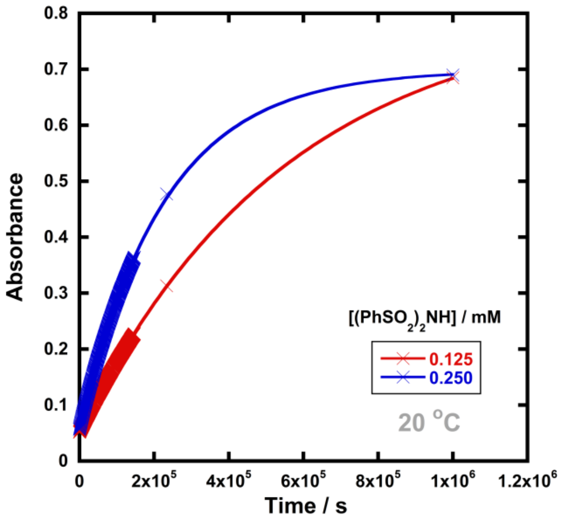

Figure 20: The relaxation of **4a-keto** (0.025 mM) in the presence of different concentrations of $(PhSO_2)_2NH$, monitored at λ_{max} = 341 nm in MeCN at 20 °C.

Table 5: k_{obs} values for the relaxation of **4a-keto** (0.025 mM) in the presence of different concentrations of $(PhSO_2)_2NH$.

$[(PhSO_2)_2NH]$ / mM	k_{obs} (relaxation) / s^{-1}	$t_{1/2}$ / min
0.125	1.79×10^{-6}	6454
0.250	4.41×10^{-6}	2620

3.4.9 With $(PhSO_2)_2N^- Na^+$ as the additive

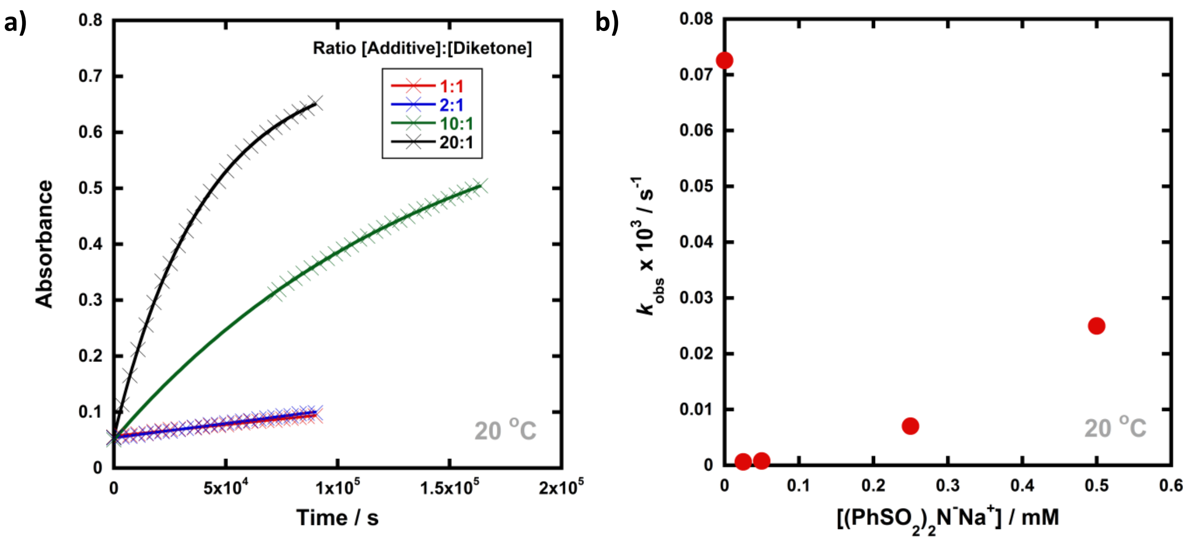

Figure 21: The relaxation of **4a-keto** (0.025 mM) in the presence of different concentrations of $(PhSO_2)_2N^- Na^+$, monitored at λ_{max} = 341 nm in MeCN at 20 °C. k_{obs} values with 1 and 2 equiv. were determined by zero-order fitting.

Table 6: k_{obs} values for the relaxation of **4a-keto** (0.025 mM) in the presence of different concentrations of $(PhSO_2)_2N^- Na^+$.

$[(PhSO_2)_2N^- Na^+]$ / mM	k_{obs} (relaxation) / s^{-1}	$t_{1/2}$ / min
0.025	6.39×10^{-7}	18086
0.050	7.96×10^{-7}	14519
0.25	7.04×10^{-6}	1641
0.50	2.50×10^{-5}	462

3.4.10 With $Bu_4N^+ BF_4^-$ as the additive

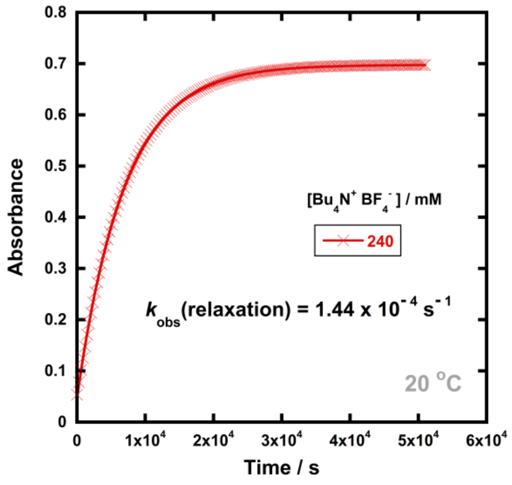

Figure 22: The relaxation of **4a-keto** (0.025 mM) monitored at λ_{max} = 341 nm in MeCN at 20 °C in the presence of $Bu_4N^+ BF_4^-$ (240 mM).

3.5 Kinetics of relaxation of 4b-d without additives and Hammett correlation

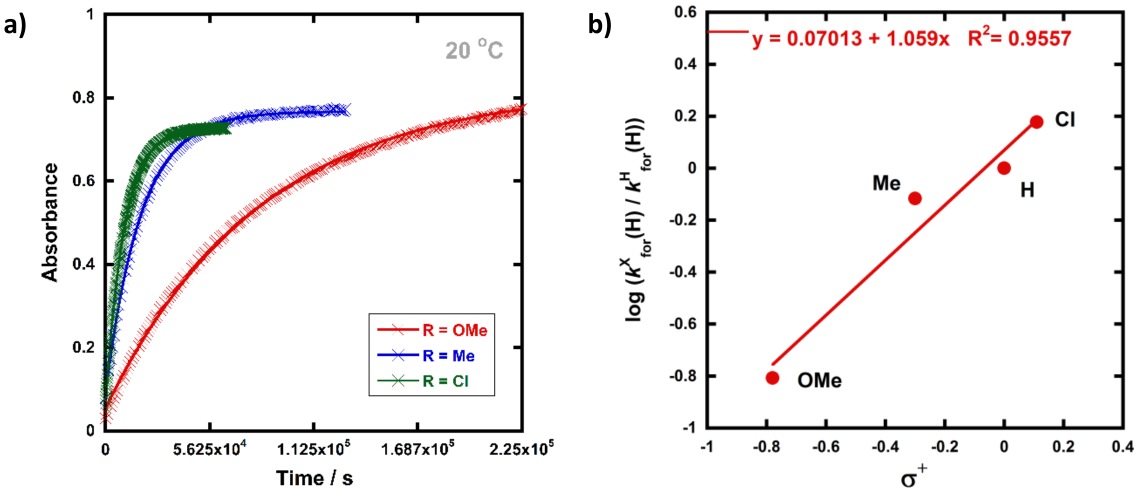

Figure 23: (a) Relaxation of keto forms of compounds **4b-d** to the equilibrium keto-enol ratios in MeCN only, monitored by UV-vis spectrophotometry at 20 °C, with scans acquired every 15 min for **4b** and **4c**, and every 2 min for **4d**. (b) Hammett correlation for conversion of keto forms of **4a-d** to the equilibrium keto-enol ratios.

Table 7: k_{obs} values obtained for the relaxation of **4a-d** (0.025 mM) at 20 °C in MeCN.

Nucleophile	Concentration / mM	k_{obs} / s^{-1}	k_{for}(H) / s^{-1}	log (k^X_{for}(H)/ k^H_{for}(H)/)
**4a**	0.025	7.26×10^{-5}	6.63×10^{-5}	0
**4b**	0.025	1.29×10^{-5}	1.03×10^{-5}	−0.807
**4c**	0.025	5.67×10^{-5}	5.04×10^{-5}	−0.117
**4d**	0.025	1.07×10^{-4}	9.91×10^{-5}	0.178

3.6 Kinetics of relaxation of 4b in the presence of additives

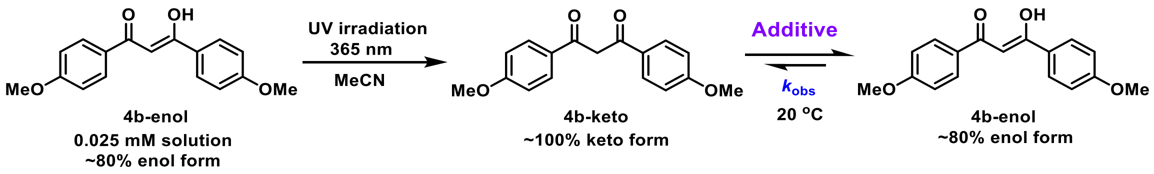

3.6.1 Additives: formic acid, DABCO

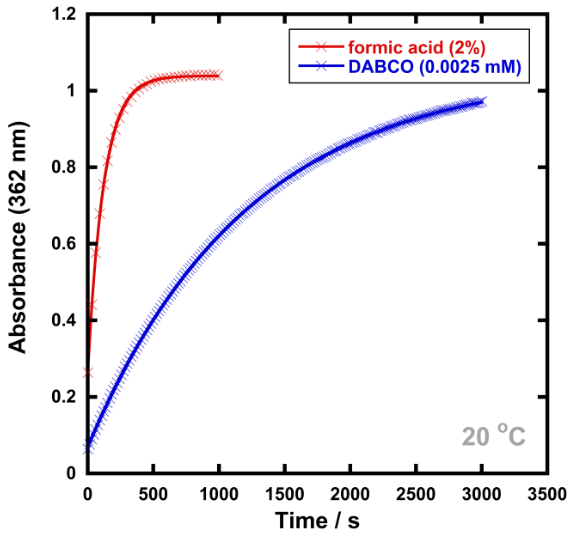

Figure 24: Relaxation of **4b-keto** (0.025 mM) in the presence of formic acid (2% in MeCN, red) and DABCO (0.0025 mM in MeCN, blue) at 20 °C. The k_{obs} values obtained are reported in Table 1 in the main text.

3.6.2 Additives: water, ClCH$_2$–DABCO tetrafluoroborate

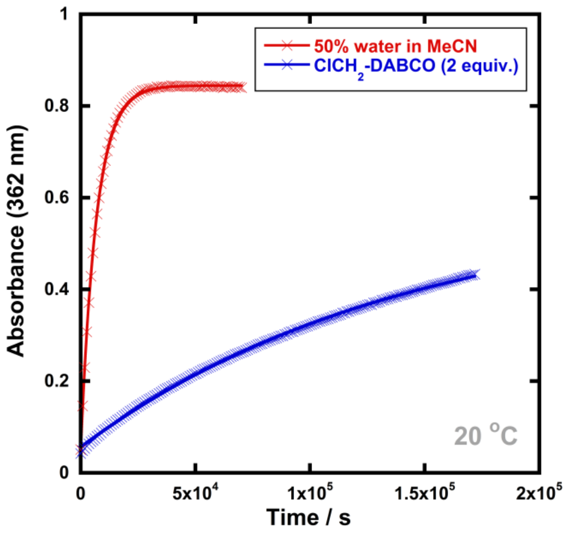

Figure 25: Relaxation of **4b-keto** (0.025 mM) in the presence of water (50% in MeCN, red) and ClCH$_2$-DABCO$^+$ BF_4^- (0.05 mM in MeCN, blue) at 20 °C. The k_{obs} values obtained are reported in Table 1 in the main text.

3.7 Kinetics of relaxation of 4c in the presence of additives

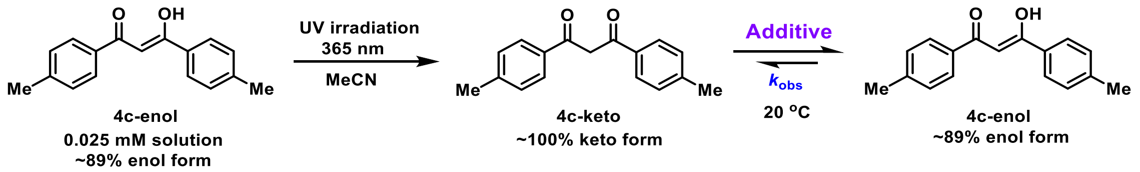

3.7.1 Additives: water, DABCO

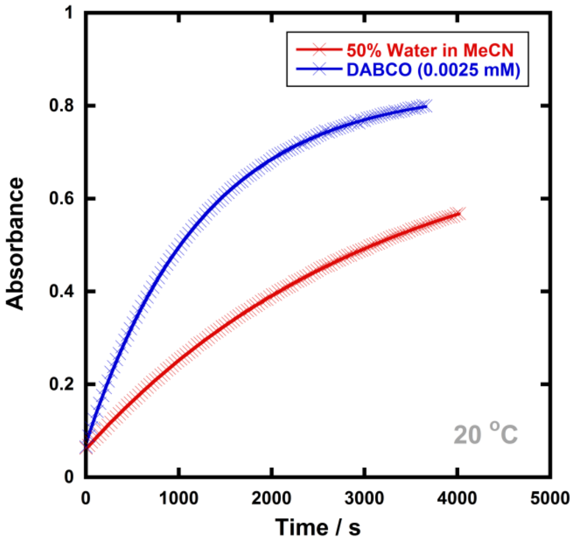

Figure 26: Relaxation of **4c-keto** (0.025 mM) in the presence of water (50% in MeCN, red) and DABCO (0.0025 mM in MeCN, blue) at 20 °C. The k_{obs} values obtained are reported in Table 1 in the main text.

3.8 Kinetics of relaxation of 4d in the presence of additives

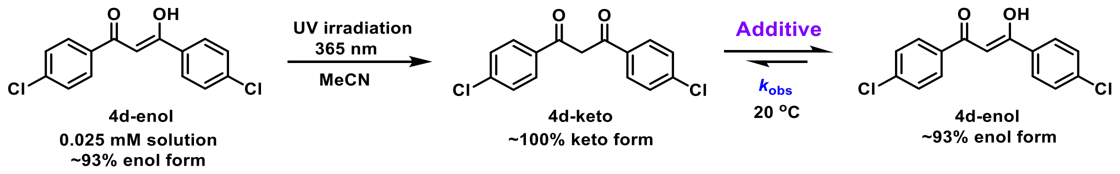

3.8.1 Additives: water, DABCO, $ClCH_2$-DABCO$^+$ BF_4^-

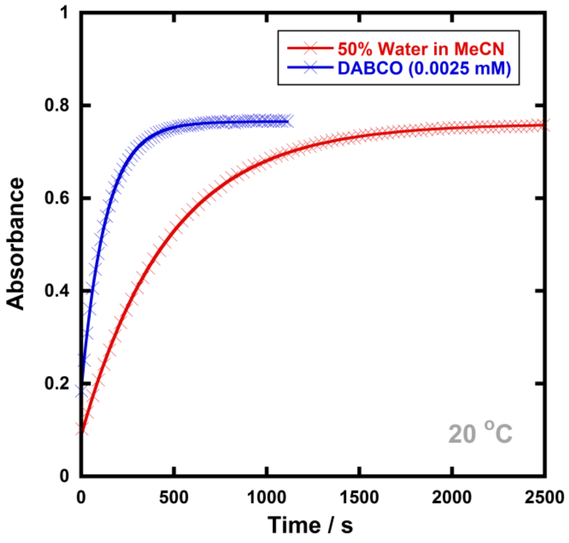

Figure 27: Relaxation of **4d-keto** (0.025 mM) in the presence of water (50% in MeCN, red) and DABCO (0.0025 mM in MeCN, blue) at 20 °C. The k_{obs} values obtained are reported in Table 1 in the main text.

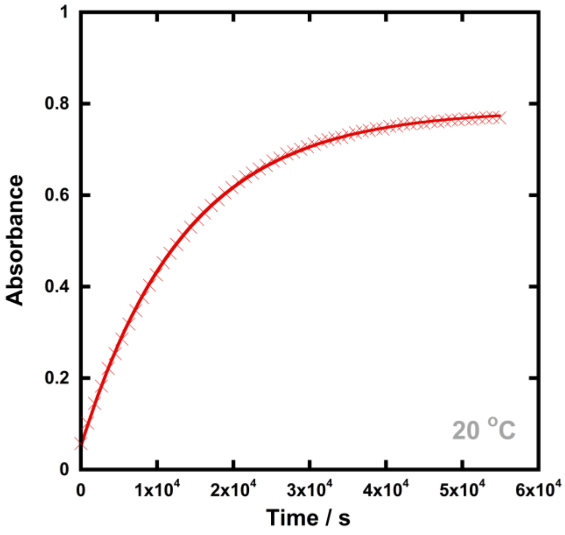

Figure 28: Relaxation of **4d-keto** (0.025 mM) in the presence of $ClCH_2$-DABCO$^+$ BF_4^- at 20 °C. The k_{obs} value obtained is reported in Table 1 in the main text.

3.9 Kinetics of relaxation of 5a

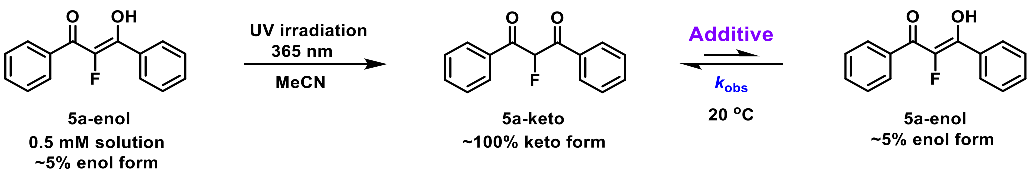

3.9.1 In the absence of additives

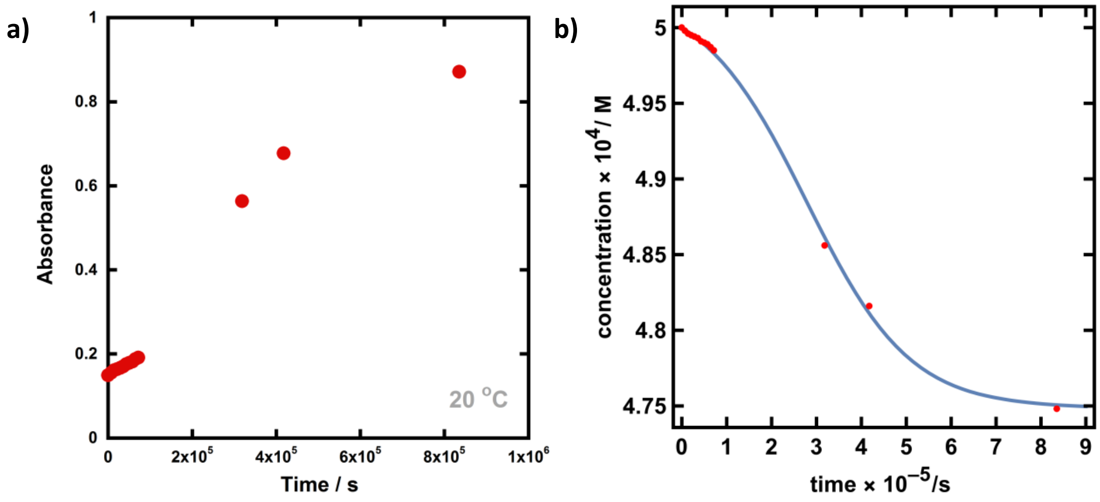

Figure 29: a) Relaxation of **5a-keto** ([**5a**$_{tot}$] = 0.5 mM) without additives (in MeCN at 20 °C, absorbance monitored at 350 nm), following conversion of the fluoroenol tautomer to the fluoroketo form by irradiation with UV light at 365 nm. b) Fitted using Wolfram Mathematica.

Fitting of Autocatalytic Model with Wolfram Mathematica 11.0:

Absorbance-time data were transformed to concentration-time data, based on the assumptions that [ketone]$_{time=0}$=0.0005 M and [ketone]$_{time=infinity}$=0.0004748 M, where this latter value was determined from the value of K_e measured by NMR spectroscopy in MeCN-d_3. The resulting data (dataset1, below) were then minimised to a model for relaxation including autocatalysis:

```
Clear[k1, k2]; totaltime = 900000;

dataset1 = {{2.35, 0.0005}, {7201.8, 0.0004998},{14402, 0.0004996},{21602,
0.0004995},{28802, 0.0004994},{36002, 0.0004993},{43202, 0.0004991},{50402,
0.0004990},{57602, 0.0004989},{64801, 0.0004987},{72002, 0.0004985},{318600,
0.0004856},{417600, 0.0004816},{835200, 0.0004748}};

model = ParametricNDSolveValue[{a'[t] == -k1*a[t] - k2*a[t]*b[t] + (k1/0.053) b[t]
+ (k2/0.053) b[t]*b[t], b'[t] == k1*a[t] + k2*a[t]*b[t] - (k1/0.053) b[t] -
(k2/0.053) b[t]*b[t], a[0] == 0.0005, b[0] == 0}, a, {t, 0, totaltime}, {k1, k2}];
```

```
fit = FindFit[dataset1, model[k1, k2][t], {{k1, 0.0000005}, {k2, 0.01}}, t]
```

The fitting delivered k_1 -> 3.6591 × 10^{-8}, k_2 -> 0.0158053, where k_1 represents the first order rate constant for uncatalysed enolization (s^{-1}) and k_2 represents the second order rate constant for autocatalysed enolization ($M^{-1}\ s^{-1}$). Reverse rate constants for the processes described by k_1 and k_2 were obtained via K_e.

3.9.2 With water as the additive

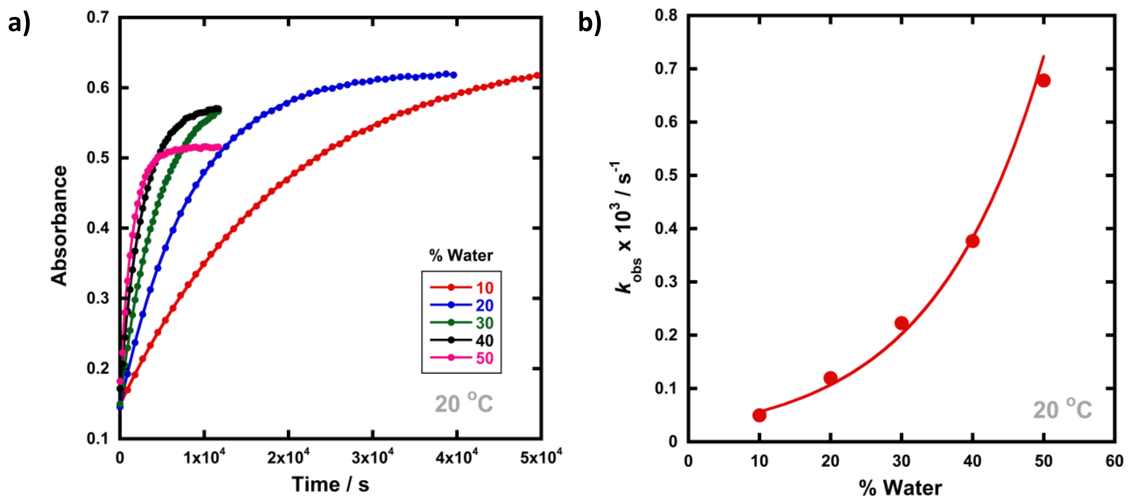

Figure 30: a) The relaxation of **5a-keto** ([**5a**$_{tot}$] = 0.5 mM) in the presence of different percentages of deionised water in MeCN, monitored at λ_{max} = 350 nm at 20 °C. The k_{obs} values obtained are reported in Table 2 of the main text. b) The trend observed in the rates of relaxation (k_{obs}) of **5a-keto**.

3.9.3 With formic acid as the additive

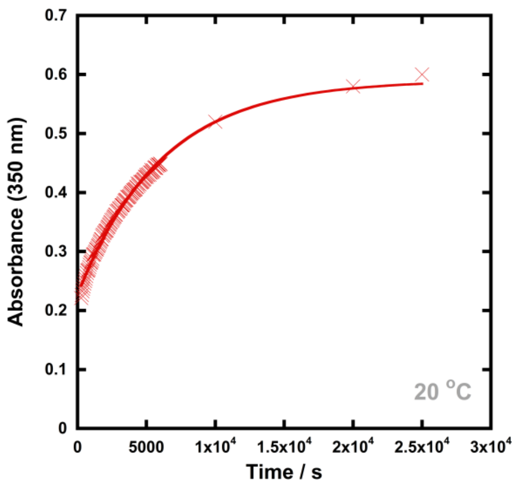

Figure 31: The relaxation of **5a-keto** ([**5a**$_{tot}$] = 0.5 mM) in the presence of formic acid (3% in MeCN), monitored at λ_{max} = 350 nm at 20 °C. The k_{obs} value obtained is reported in Table 2 of the main text.

3.9.4 With DABCO as the additive

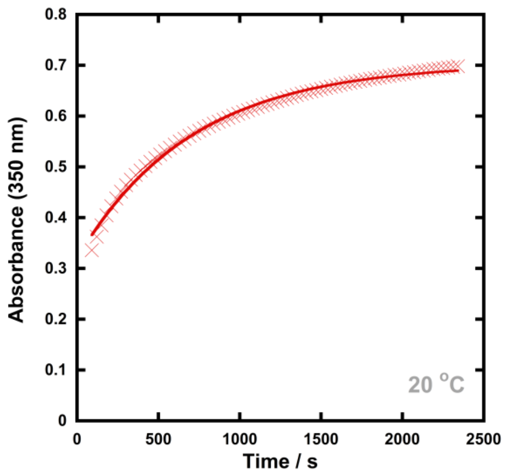

Figure 32: The de-fluorination of **5a-keto** ([**5a**$_{tot}$] = 0.5 mM) in the presence of DABCO (0.0025 mM), monitored at λ_{max} = 350 nm in MeCN at 20 °C. NMR data confirmed the loss of the fluorine atom.

3.9.5 With water and 0.0125 mM $ClCH_2$-DABCO$^+$ BF_4^-

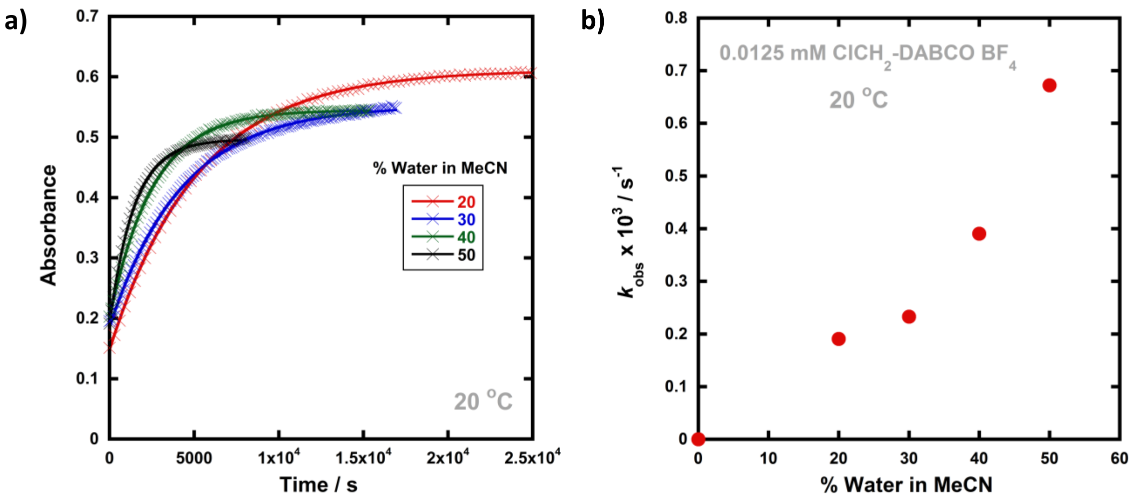

Figure 33: The relaxation of **5a-keto** ([**5a**$_{tot}$] = 0.5 mM) in the presence of 20% water and $ClCH_2$-DABCO$^+$ BF_4^- (0.0125 mM), monitored at λ_{max} = 350 nm in MeCN at 20 °C.

Table 8: k_{obs} values for the relaxation of **5a-keto**.

Experiment	Quantity of water in MeCN / %	$ClCH_2$-DABCO$^+$ BF_4^- / mM	$k_{obs} \times 10^3$ / s^{-1}
**1**	0	0.0125	0.0010 ± 0.0001
**2**	20	0.0125	0.1907 ± 0.0005
**3**	30	0.0125	0.233 ± 0.001
**4**	40	0.0125	0.390 ± 0.001
**5**	50	0.0125	0.694 ± 0.004

3.9.6 With $ClCH_2$-DABCO$^+$ BF_4^- as the additive

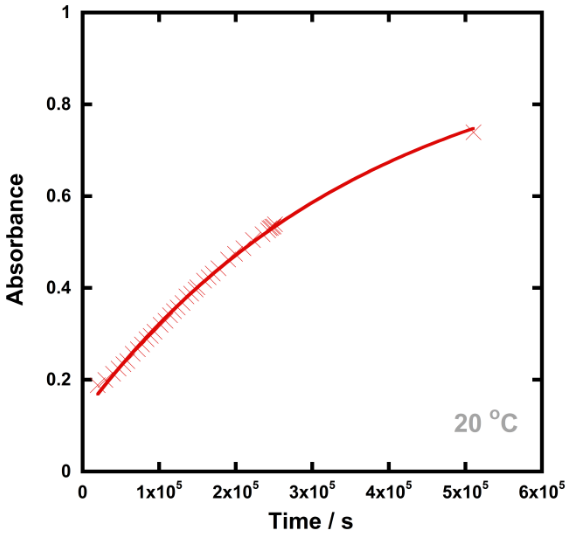

Figure 34: Relaxation of **5a-keto** ([**5a**$_{tot}$] = 0.5 mM) in the presence of $ClCH_2$-DABCO$^+$ BF_4^- (0.025 mM), monitored at λ_{max} = 350 nm in MeCN at 20 °C. The k_{obs} value obtained is reported in Table 2 of the main text.

3.9.7 With Bu_4N^+ BF_4^- as the additive

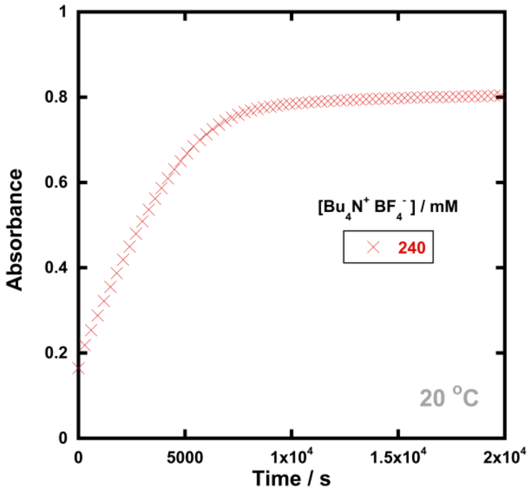

Figure 35: The relaxation of **5a-keto** ([**5a**$_{tot}$] = 0.5 mM) monitored at λ_{max} = 350 nm in MeCN at 20 °C in the presence of Bu_4N^+ BF_4^- (240 mM).

3.10 Kinetics of relaxation of 5b

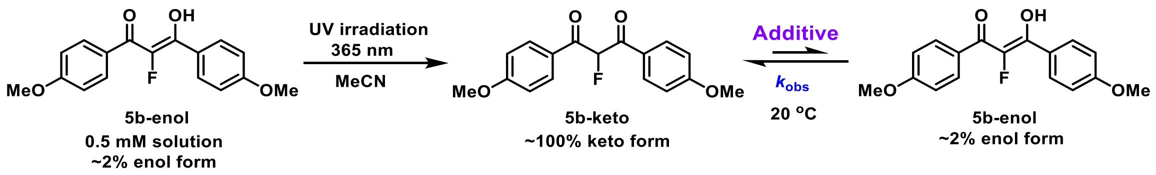

3.10.1 In the absence of additives

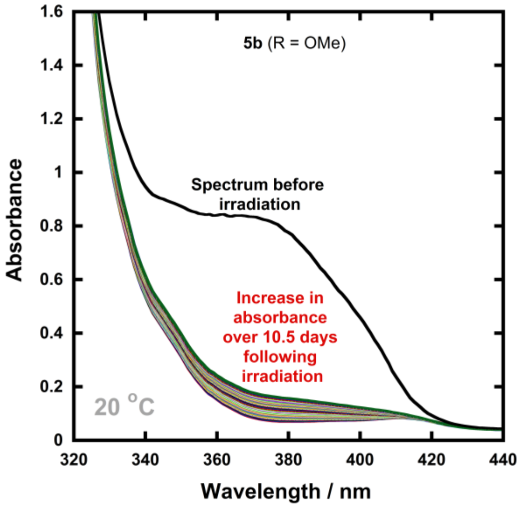

Figure 36: Absorbance spectra for re-enolization of **5b-keto** (0.5 mM, 20 °C, spectra acquired every 6 h over 10.5 days) following irradiation to the diketone tautomer. The relaxation was very slow and did not reach the endpoint. The black line corresponds to the spectrum before irradiation, from which the assumed endpoint was obtained.

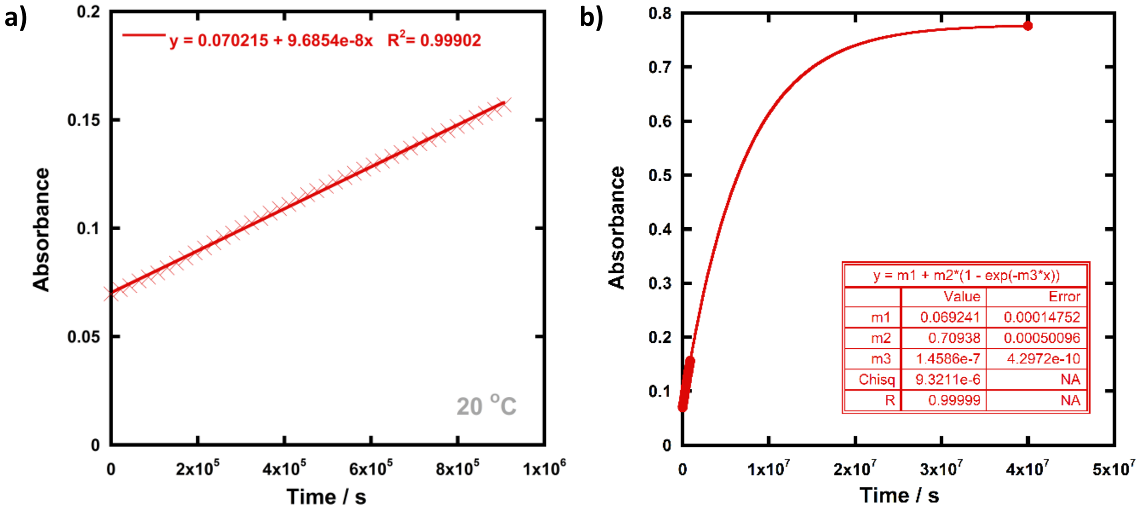

Figure 37: Relaxation of **5b-keto** ([**5b**$_{total}$] = 0.5 mM) without additives (in MeCN at 20 °C, absorbance monitored at 380 nm), following conversion of the fluoroenol tautomer to the fluoroketo form by irradiation with UV light at 365 nm. Two different types of fitting are presented: (a) Linear fitting of data points to obtain the rate constant using a zero-order approach. (b) First order fitting with a fixed endpoint.

In Figure 37a, the relaxation rate was measured using a zero-order fitting:

k_{obs} = slope / total absorbance change = $9.6854 \times 10^{-8} / (0.77656 - 0.069684) = 1.37 \times 10^{-7}\ s^{-1}$

As shown in Figure 37b, the rate constant obtained from extrapolation to the endpoint is $1.46 \times 10^{-7}\ s^{-1}$. The two rate constants are within 6% of each other.

3.10.2 With water as the additive

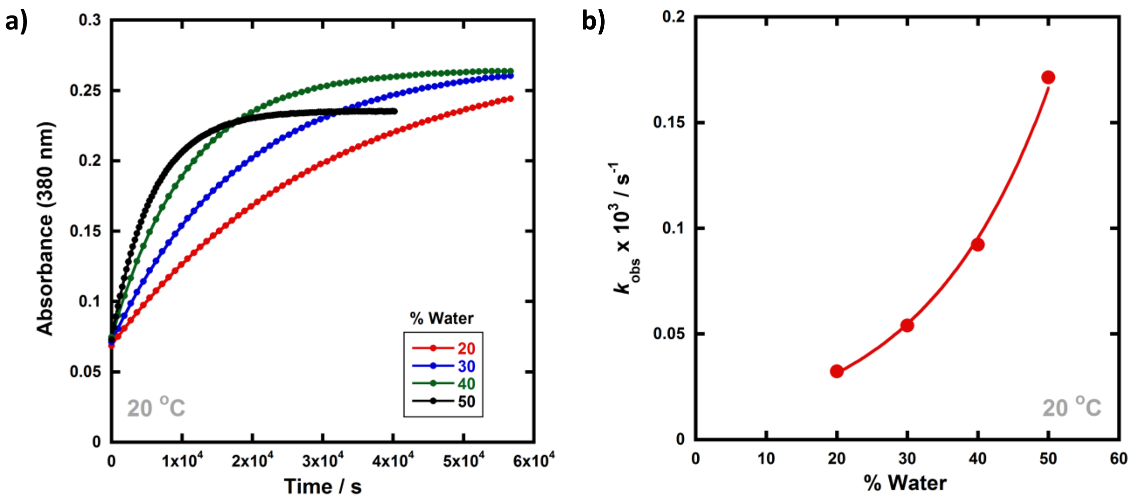

Figure 38: (a) Relaxation of **5b-keto** ([**5b**$_{tot}$] = 0.5 mM) following conversion of the fluoroenol tautomer to the fluoroketo form by irradiation with UV light at 365 nm. (b) Correlation of k_{obs} values for relaxation, obtained with different quantities of water at 20 °C. The k_{obs} values obtained are reported in Table 2 of the main text.

3.10.3 Other additives: formic acid, DABCO

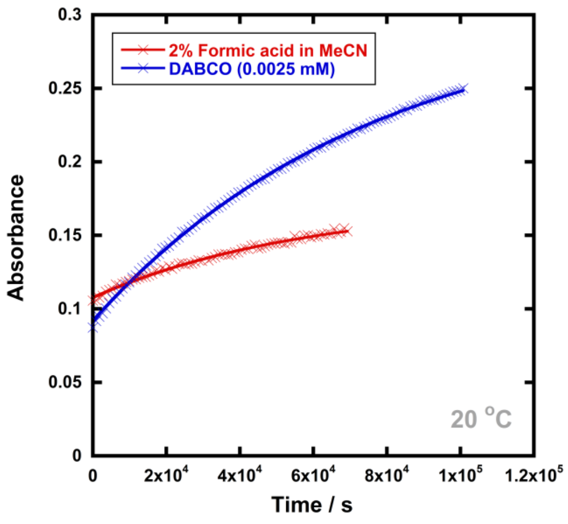

Figure 39: Relaxation of **5b-keto** ([**5b**$_{tot}$] = 0.5 mM) in the presence of additives: formic acid (2% in MeCN, red), DABCO (0.0025 mM in MeCN, blue) at 20 °C. The k_{obs} values obtained are reported in Table 2 of the main text.

3.11 Kinetics of relaxation of 5c

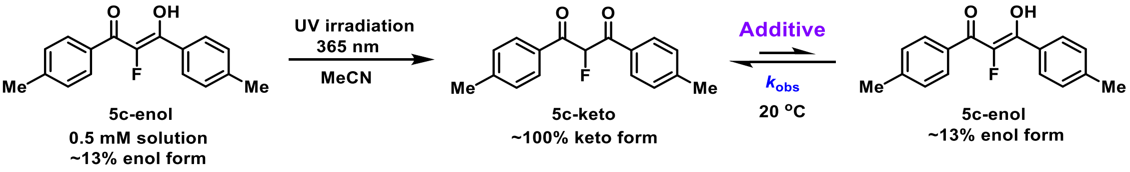

3.11.1 In the absence of additives

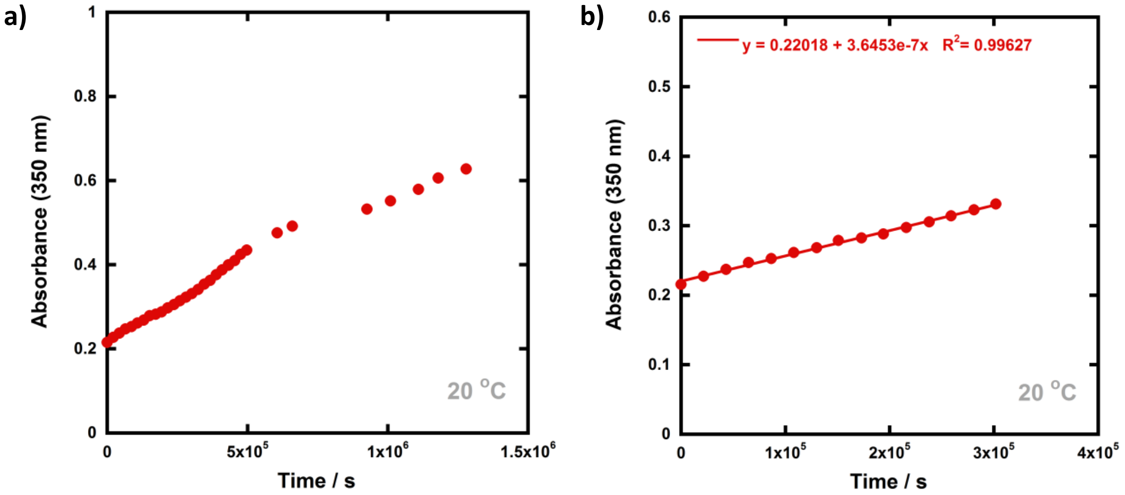

Figure 40: a) Relaxation of **5c-keto** ([**5c**$_{tot}$] = 0.5 mM) at 20 °C, following conversion of the fluoroenol tautomer to the fluoroketo form by irradiation with UV light at 365 nm. b) Linear fitting to first 20% of relaxation is shown.

The rate constant for relaxation was obtained using a zero-order approach:

k_{obs} = slope / total absorbance change = 3.6453×10^{-7} / (0.6277 - 0.2156) = 8.8457×10^{-7} s^{-1}

3.11.2 With water as the additive

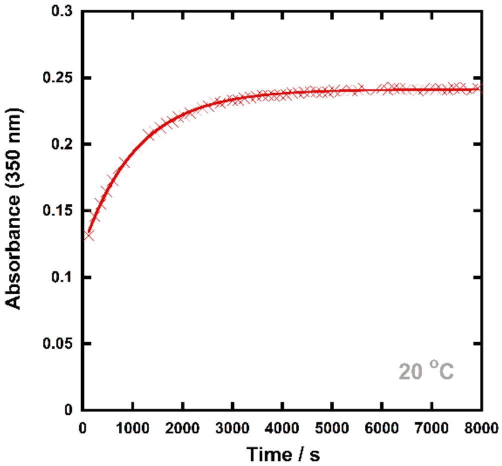

Figure 41: Relaxation of **5c-keto** ([**5c**$_{tot}$] = 0.25 mM) with 50% water in MeCN at 20 °C, following conversion of the fluoroenol tautomer to the fluoroketo form by irradiation with UV light at 365 nm.

3.12 Kinetics of relaxation of 5d

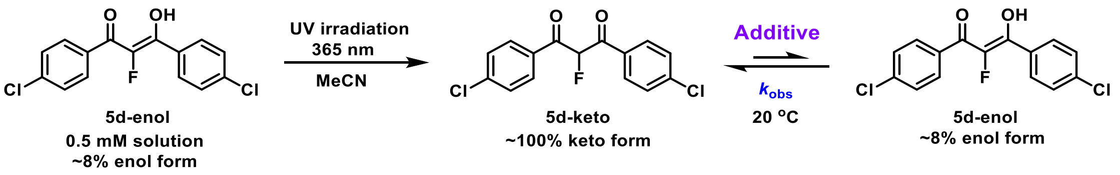

3.12.1 In the absence of additives

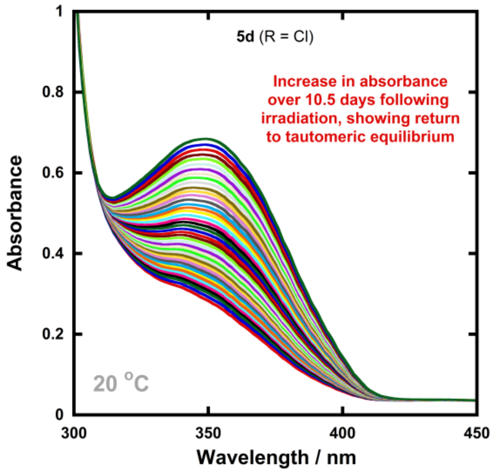

Figure 42: Absorbance spectra for relaxation of **5d-keto** (0.5 mM, 20 °C, spectra acquired every 6 h over 10.5 days), returning to the tautomeric equilibrium.

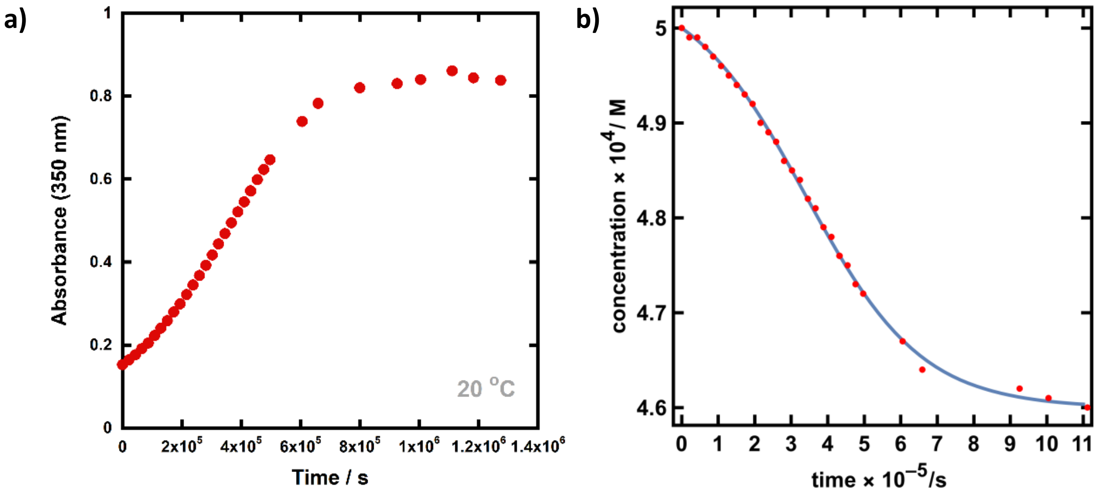

Figure 43: a) Relaxation of **5d-keto** ([**5d**$_{tot}$] = 0.5 mM) at 20 °C, following conversion of the fluoroenol tautomer to the fluoroketo form by irradiation with UV light at 365 nm. b) Fitted using Wolfram Mathematica.

Fitting of Autocatalytic Model with Wolfram Mathematica:

Absorbance-time data were transformed to concentration-time data, based on the assumptions that $[\text{ketone}]_{\text{time=0}}$=0.0005 M and $[\text{ketone}]_{\text{time=infinity}}$=0.000460 M, where this latter value was determined

from the value of K_e measure by NMR spectroscopy in MeCN-d_3. The resulting data (dataset1, below) were then minimised to a model for relaxation including autocatalysis:

```
Clear[k1, k2]; totaltime = 1200000;

dataset1 = {{4.65,0.000500},{21604,0.000499},{43204,0.000499},{64803,
0.000498},{86404,0.000497},{108000,0.000496},{129600,0.000495},{151200,0.000494},{1
72800,0.000493},{194400,0.000492},{216000,0.000490},{237600,0.000489},{259200,0.000
488},{280800,0.000486},{302400,0.000485},{324000,0.000484},{345600,0.000482},{36720
0,0.000481},{388800,0.000479},{410400,0.000478},{432000,0.000476},{432900,0.000476}
,{454500,0.000475},{476100,0.000473},{497700,0.000472},{605700,0.000467},{659700,0.
000464},{926100,0.000462},{1005300,0.000461},{1111500,0.000460}};

model = ParametricNDSolveValue[{a'[t]==-k1*a[t]-
k2*a[t]*b[t]+(k1/0.087)b[t]+(k2/0.087) b[t]*b[t], b'[t]==k1*a[t]+k2*a[t]*b[t]-
(k1/0.087)b[t]-(k2/0.087)b[t]*b[t], a[0]==0.0005, b[0] == 0}, a, {t, 0, totaltime},
{k1, k2}];

fit = FindFit[dataset1,model[k1, k2][t], {{k1, 0.0000005}, {k2, 0.03}}, t]
```

The fitting delivered k_1 -> 5.37818 × 10^{-8}, k_2 -> 0.0111529, where k_1 represents the first order rate constant for uncatalysed enolization (s^{-1}) and k_2 represents the second order rate constant for autocatalysed enolization (M^{-1} s^{-1}). Reverse rate constants for the processes described by k_1 and k_2 were obtained via K_e.

3.12.2 With water as the additive

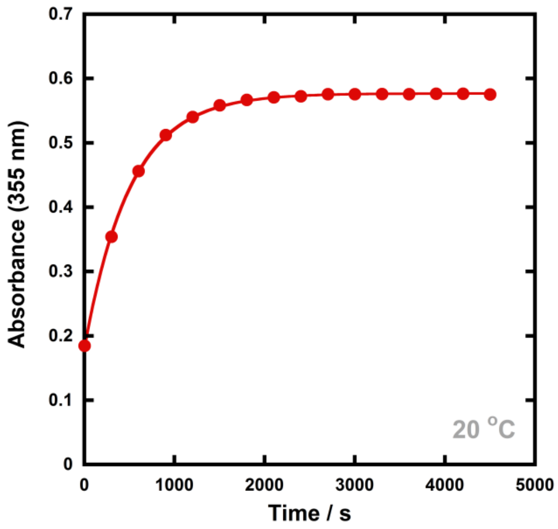

Figure 44: Relaxation of **5d-keto** ([**5d$_{tot}$**] = 0.5 mM) with 50% water in MeCN at 20 °C, following conversion of the fluoroenol tautomer to the fluoroketo form by irradiation with UV light at 365 nm. The k_{obs} value obtained is reported in Table 2 of the main text.

3.13 Kinetics of fluorination of 5a-d by Selectfluor™

3.13.1 Fluorination of 5a

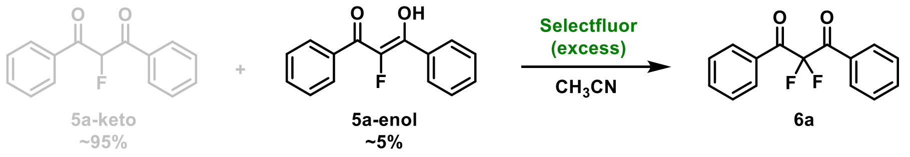

0.5 mM solution, but since ~5% of this is enol tautomer, [5a-enol] = 0.025 mM

At 20 °C:

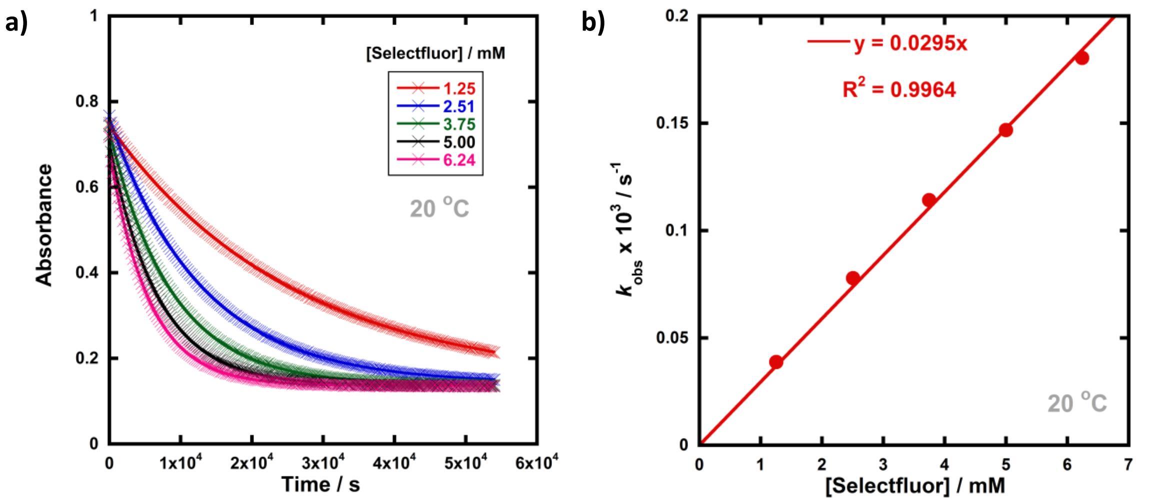

Figure 45: (a) Exponential decays of absorbance with different concentrations of Selectfluor™ in MeCN at 20 °C, monitored at λ_{max} = 350 nm. (b) Correlation of k_{obs} with [Selectfluor™].

Table 9: k_{obs} values at different concentrations of Selectfluor™ at 20 °C. Errors are standard error values. The concentration of **5a-enol** is 0.025 mM.

Experiment	Ratio of [Selectfluor] to [5a-enol]	[Selectfluor] / mM	[5a$_{total}$] / mM	$k_{obs} \times 10^3$ / s^{-1}
**1**	50:1	1.25	0.5	0.0388 ± 0.0008
**2**	100:1	2.50	0.5	0.0779 ± 0.0009
**3**	150:1	3.75	0.5	0.114 ± 0.002
**4**	200:1	5.00	0.5	0.147 ± 0.003
**5**	250:1	6.24	0.5	0.181 ± 0.006

At 25 °C:

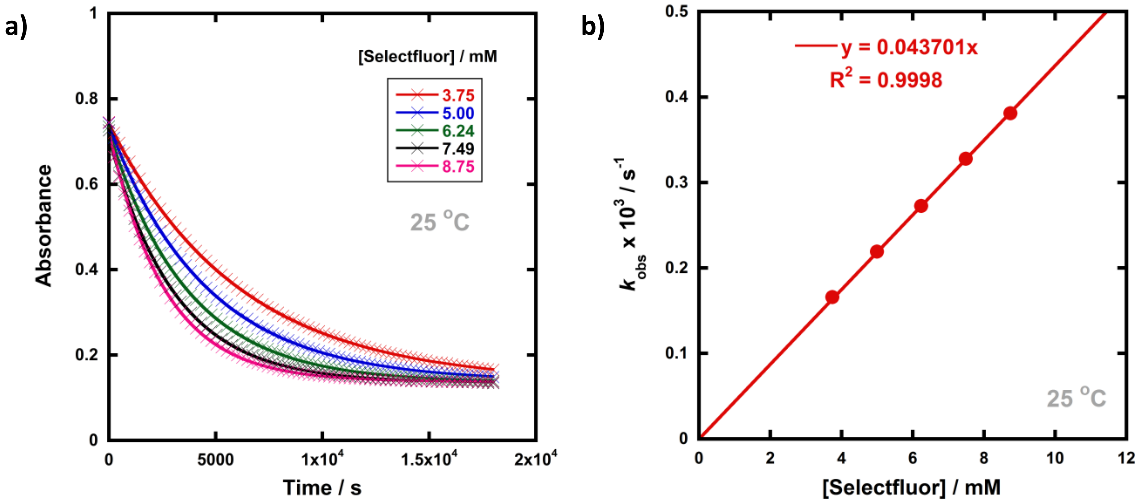

Figure 46: (a) Exponential decays of absorbance with different concentrations of Selectfluor™ in MeCN at 25 °C, monitored at λ_{max} = 350 nm. (b) Correlation of k_{obs} with [Selectfluor™].

Table 10: k_{obs} values at different concentrations of Selectfluor™ at 25 °C. Errors are standard error values.

Experiment	Ratio of [Selectfluor] to [5a-enol]	[Selectfluor] / mM	[5a$_{total}$] / mM	$k_{obs} \times 10^3$ / s^{-1}
**1**	150:1	3.75	0.5	0.1658 ± 0.0004
**2**	200:1	5.00	0.5	0.2191 ± 0.0006
**3**	250:1	6.24	0.5	0.273 ± 0.001
**4**	300:1	7.49	0.5	0.328 ± 0.002
**5**	350:1	8.75	0.5	0.381 ± 0.002

At 30 °C:

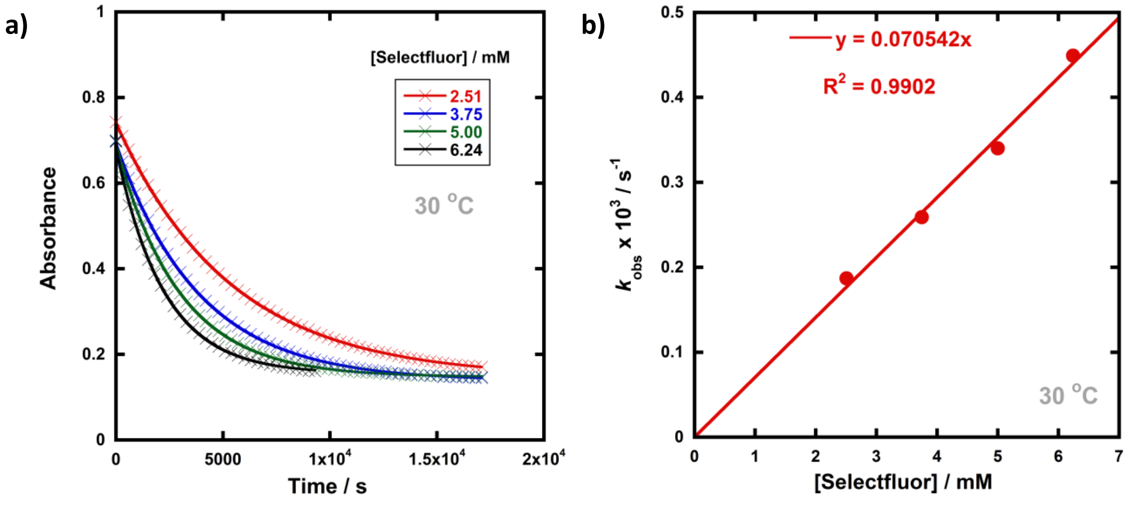

Figure 47: (a) Exponential decays of absorbance with different concentrations of Selectfluor™ in MeCN at 30 °C, monitored at λ_{max} = 350 nm. (b) Correlation of k_{obs} with [Selectfluor™].

Table 11: k_{obs} values at different concentrations of Selectfluor™ at 30 °C. Errors are standard error values.

Experiment	Ratio of [Selectfluor] to [5a-enol]	[Selectfluor] / mM	[$5a_{total}$] / mM	$k_{obs} \times 10^3$ / s^{-1}
**1**	100:1	2.51	0.5	0.1871 ± 0.0004
**2**	150:1	3.75	0.5	0.2592 ± 0.0007
**3**	200:1	5.00	0.5	0.340 ± 0.001
**4**	250:1	6.24	0.5	0.449 ± 0.006

At 35 °C

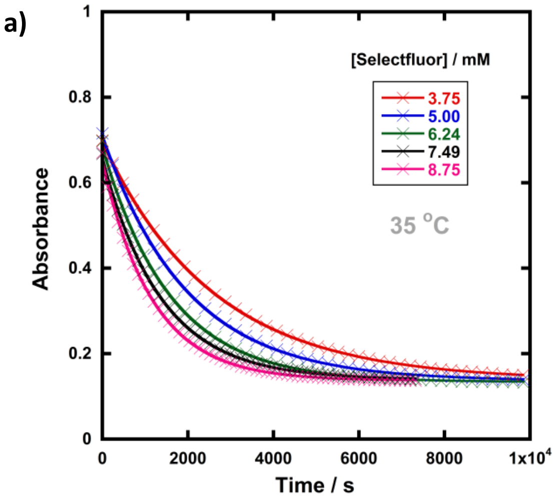

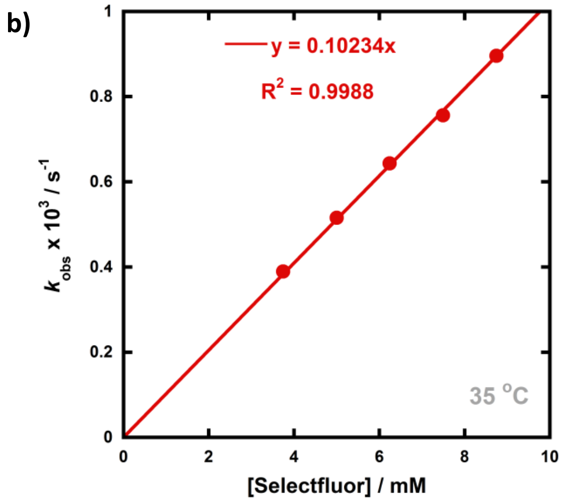

Figure 48: (a) Exponential decays of absorbance with different concentrations of Selectfluor™ in MeCN at 35 °C, monitored at λ_{max} = 350 nm. (b) Correlation of k_{obs} with [Selectfluor™].

Table 12: k_{obs} values at different concentrations of Selectfluor™ at 35 °C. Errors are standard error values.

**Experiment**	**Ratio of [Selectfluor] to [5a-enol]**	**[Selectfluor] / mM**	**[5a$_{total}$] / mM**	$k_{obs} \times 10^3$ / s^{-1}
**1**	150:1	3.75	0.5	0.3864 ± 0.0009
**2**	200:1	5.00	0.5	0.508 ± 0.002
**3**	250:1	6.24	0.5	0.631 ± 0.005
**4**	300:1	7.49	0.5	0.728 ± 0.003
**5**	350:1	8.75	0.5	0.850 ± 0.007

3.13.2 Fluorination of 5b

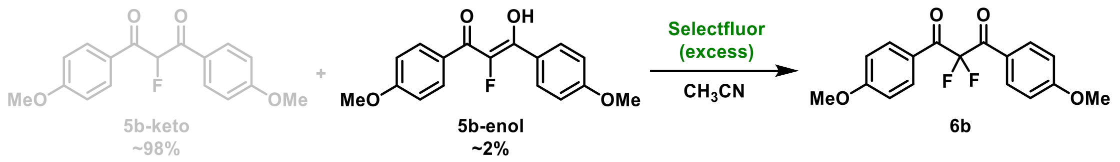

At 20 °C:

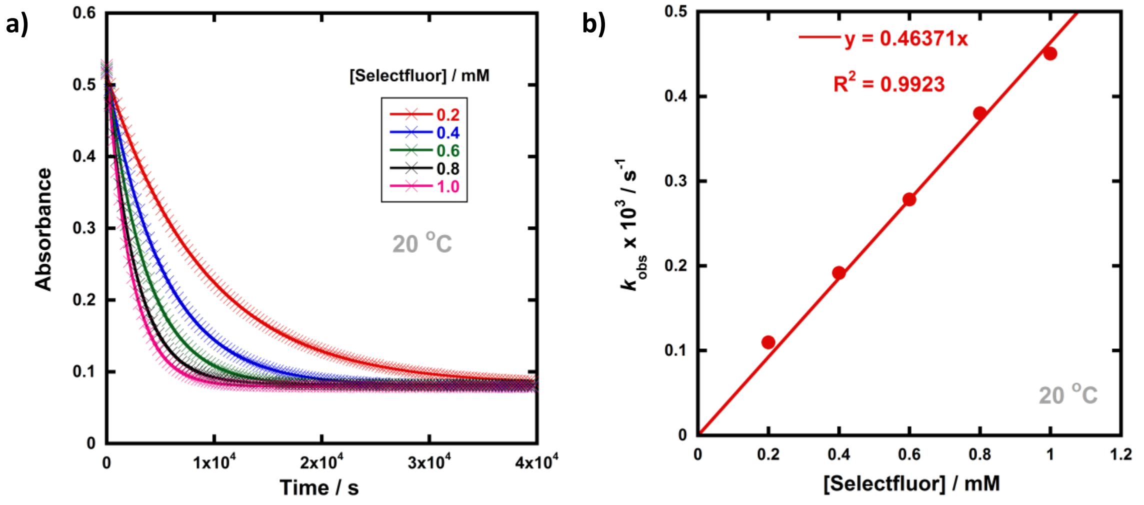

Figure 49: (a) Exponential decays of absorbance with different concentrations of Selectfluor™ at 20 °C in MeCN, monitored at 380 nm. (b) Correlation of k_{obs} with [Selectfluor™].

Table 13: k_{obs} values at different concentrations of Selectfluor™ at 20 °C. Errors are standard error values. The concentration of **5b-enol** is 0.01 mM.

Experiment	Ratio of [Selectfluor] to [5b-enol]	[Selectfluor] / mM	[$5b_{total}$] / mM	$k_{obs} \times 10^3$ / s^{-1}
**1**	20:1	0.2	0.5	0.1109 ± 0.0001
**2**	40:1	0.4	0.5	0.1918 ± 0.0001
**3**	60:1	0.6	0.5	0.2782 ± 0.0002
**4**	80:1	0.8	0.5	0.3801 ± 0.0003
**5**	100:1	1.0	0.5	0.4506 ± 0.0004

At 25 °C:

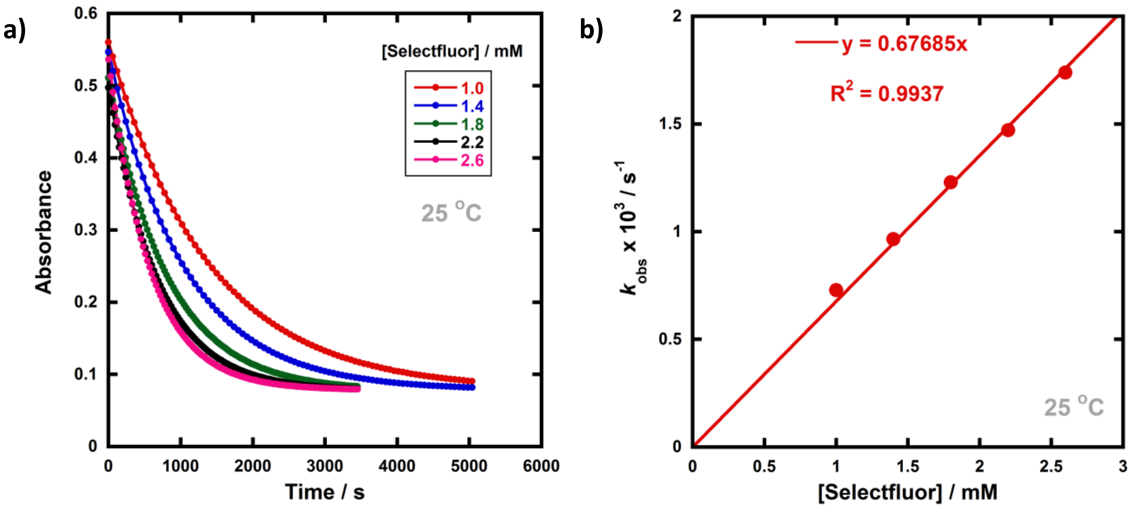

Figure 50: (a) Exponential decays of absorbance with different concentrations of Selectfluor™ at 25 °C in MeCN. (b) Correlation of k_{obs} with [Selectfluor™].

Table 14: k_{obs} values at different concentrations of Selectfluor™ at 25 °C. Errors are standard error values.

**Experiment**	**Ratio of [Selectfluor] to [5b-enol]**	**[Selectfluor] / mM**	**[5b$_{total}$] / mM**	$k_{obs} \times 10^3$ / s^{-1}
**1**	100:1	1.0	0.5	0.7280 ± 0.0003
**2**	140:1	1.4	0.5	0.9648 ± 0.0003
**3**	180:1	1.8	0.5	1.2288 ± 0.0003
**4**	220:1	2.2	0.5	1.4714 ± 0.0003
**5**	260:1	2.6	0.5	1.7386 ± 0.0005

At 30 °C:

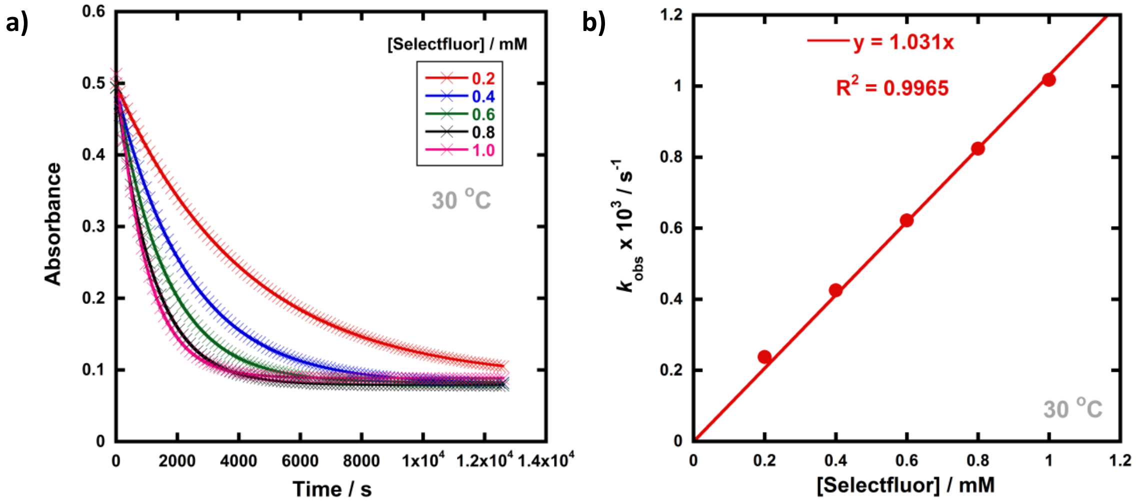

Figure 51: (a) Exponential decays of absorbance with different concentrations of Selectfluor™ at 30 °C in MeCN. (b) Correlation of k_{obs} with [Selectfluor™].

Table 15: k_{obs} values at different concentrations of Selectfluor™ at 30 °C. Errors are standard error values.

Experiment	Ratio of [Selectfluor] to [5b-enol]	[Selectfluor] / mM	[5b$_{total}$] / mM	$k_{obs} \times 10^3$ / s^{-1}
**1**	20:1	0.2	0.5	0.2378 ± 0.0003
**2**	40:1	0.4	0.5	0.4253 ± 0.0004
**3**	60:1	0.6	0.5	0.6215 ± 0.0004
**4**	80:1	0.8	0.5	0.8239 ± 0.0004
**5**	100:1	1.0	0.5	1.018 ± 0.001

At 35 °C:

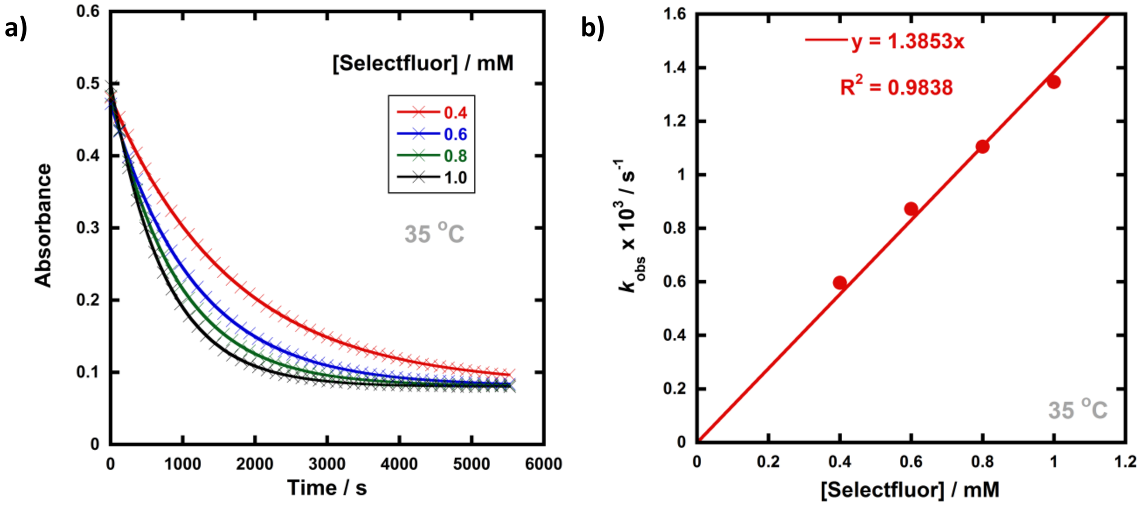

Figure 52: (a) Exponential decays of absorbance with different concentrations of Selectfluor™ at 35 °C in MeCN. (b) Correlation of k_{obs} with [Selectfluor™].

Table 16: k_{obs} values at different concentrations of Selectfluor™ at 35 °C. Errors are standard error values.

Experiment	Ratio of [Selectfluor] to [5b-enol]	[Selectfluor] / mM	[$5b_{total}$] / mM	$k_{obs} \times 10^3$ / s^{-1}
**1**	40:1	0.4	0.5	0.5965 ± 0.0008
**2**	60:1	0.6	0.5	0.8721 ± 0.0009
**3**	80:1	0.8	0.5	1.105 ± 0.002
**4**	100:1	1.0	0.5	1.346 ± 0.002

In order to confirm that the product of this reaction is indeed **6b**, LC-MS analysis was carried out on the reaction mixture in the cuvette at the end of the reaction (shown on page S44, 30 °C). The peak at R_t = 2.63 min (Figure 53) corresponds to **5b-keto**. As expected, this remains unreacted as it comprises ~98% of the keto-enol equilibrium, and as relaxation is slow it does not occur on the timescale of our fluorination reactions. The peak at R_t = 2.99 min corresponds to **6b** (m/z = 321.29).

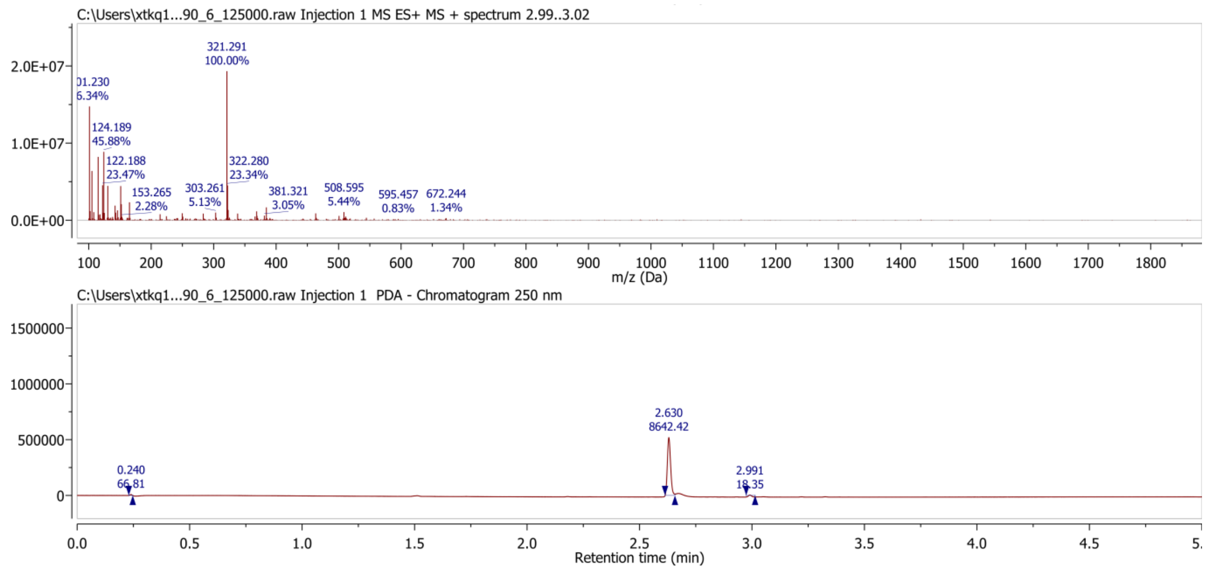

Figure 53: LC-MS spectrum of the reaction mixture inside the cuvette for fluorination of **5b-enol** by Selectfluor™.

3.13.3 Fluorination of 5c

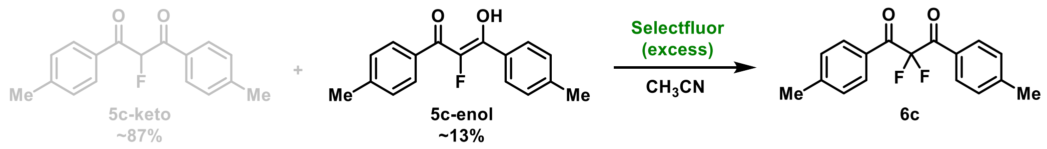

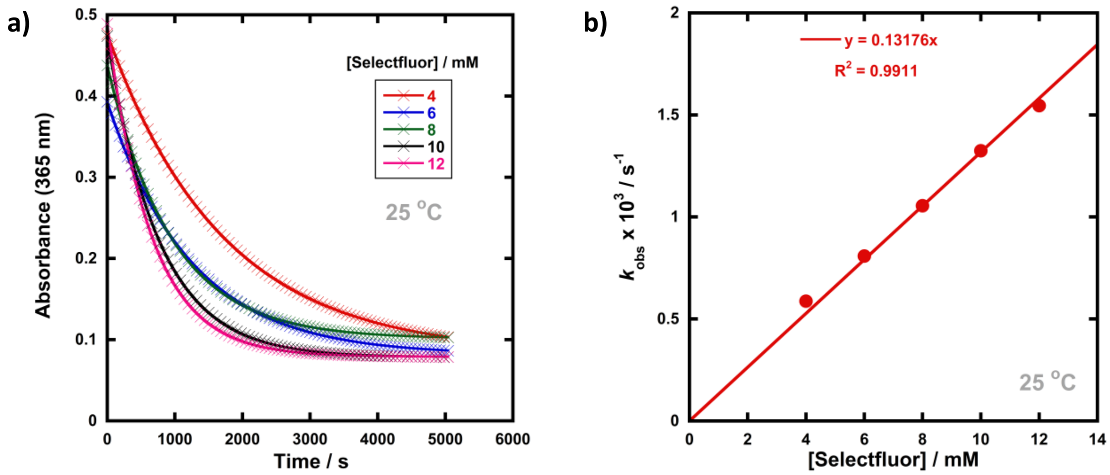

Figure 54: (a) Exponential decays of absorbance with different concentrations of Selectfluor™. (b) Correlation of k_{obs} with [Selectfluor™].

Table 17: k_{obs} values at different concentrations of Selectfluor™ at 25 °C. Errors are standard error values. The concentration of **5c-enol** is 0.052 mM.

Experiment	Ratio of [Selectfluor] to [5c-enol]	[Selectfluor] / mM	[$5c_{total}$] / mM	$k_{obs} \times 10^3$ / s^{-1}
**1**	77:1	4.0	0.4	0.5887 ± 0.0003
**2**	115:1	6.0	0.4	0.8075 ± 0.0005
**3**	154:1	8.0	0.4	1.051 ± 0.001
**4**	192:1	10.0	0.4	1.318 ± 0.001
**5**	231:1	12.0	0.4	1.540 ± 0.002

3.13.4 Fluorination of 5d

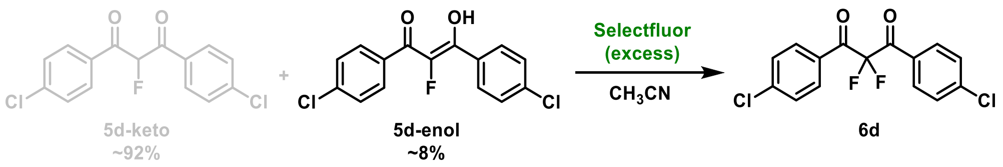

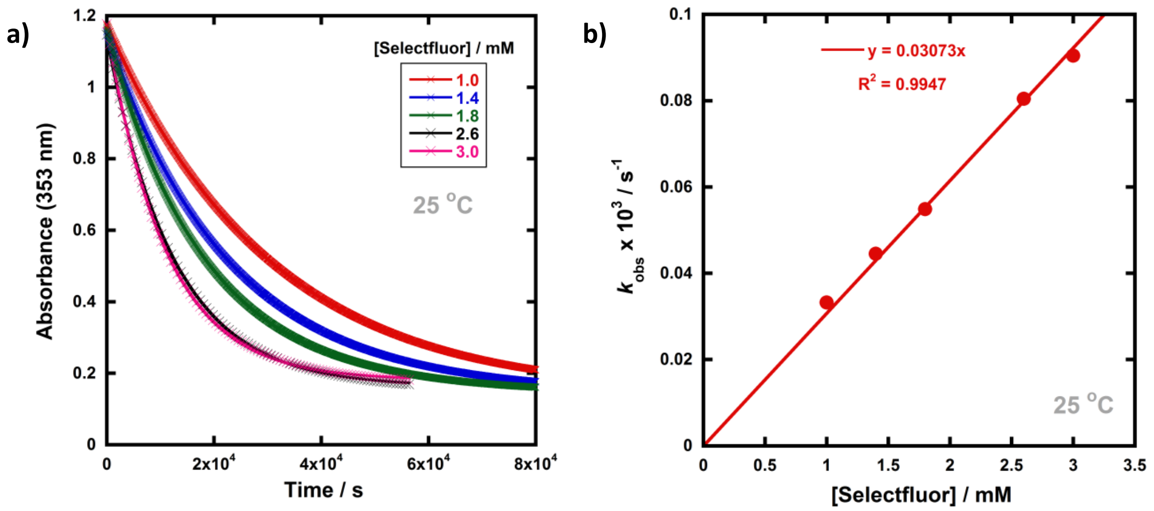

Figure 55: (a) Exponential decays of absorbance with different concentrations of Selectfluor™. (b) Correlation of k_{obs} with [Selectfluor™].

Table 18: k_{obs} values at different concentrations of Selectfluor™ at 25 °C. Errors are standard error values. The concentration of **5d-enol** is 0.04 mM.

Experiment	Ratio of [Selectfluor] to [5d-enol]	[Selectfluor] / mM	[$5d_{total}$] / mM	$k_{obs} \times 10^4$ / s^{-1}
**1**	25:1	1.0	0.5	0.33252 ± 0.00008
**2**	35:1	1.4	0.5	0.4451 ± 0.0001
**3**	45:1	1.8	0.5	0.5489 ± 0.0003
**4**	65:1	2.6	0.5	0.805 ± 0.002
**5**	75:1	3.0	0.5	0.904 ± 0.003

3.13.5 Hammett and Eyring correlations

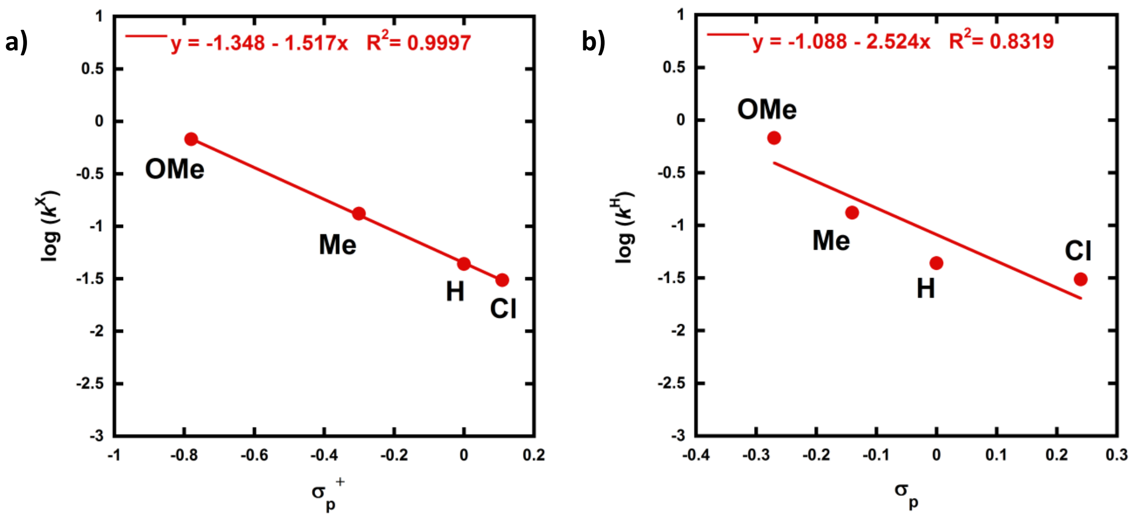

Figure 56: Hammett correlations corresponding to fluorination of **5a-d** by Selectfluor™. All rate constants were obtained in MeCN at 25 °C, and are plotted against a) σ_p^+ values, and b) σ_p values.

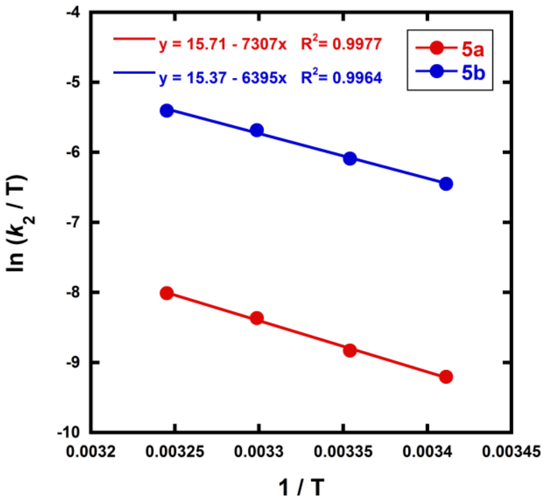

Figure 57: Eyring plots for fluorination of 2-fluoro-1,3-dicarbonyls **5a** and **5b** by Selectfluor™ in MeCN at 20 °C, 25 °C, 30 °C and 35 °C.

Table 19: Activation parameters calculated using the Eyring plots in Figure 57.

Compound	$\Delta H^‡$ / kJ mol^{-1}	$\Delta S^‡$ / J K^{-1} mol^{-1}	$\Delta G^‡$ / kJ mol^{-1}
**5a**	60.7	−66.9	80.6
**5b**	53.2	−69.7	74.0

3.14 Kinetics of fluorination of 5a-d by NFSI

3.14.1 Fluorination of 5a

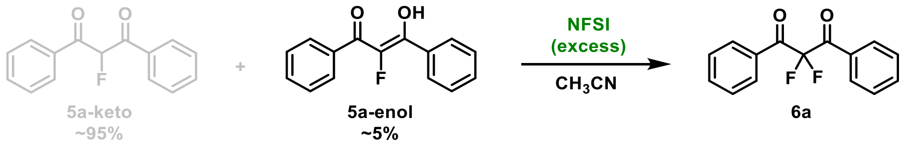

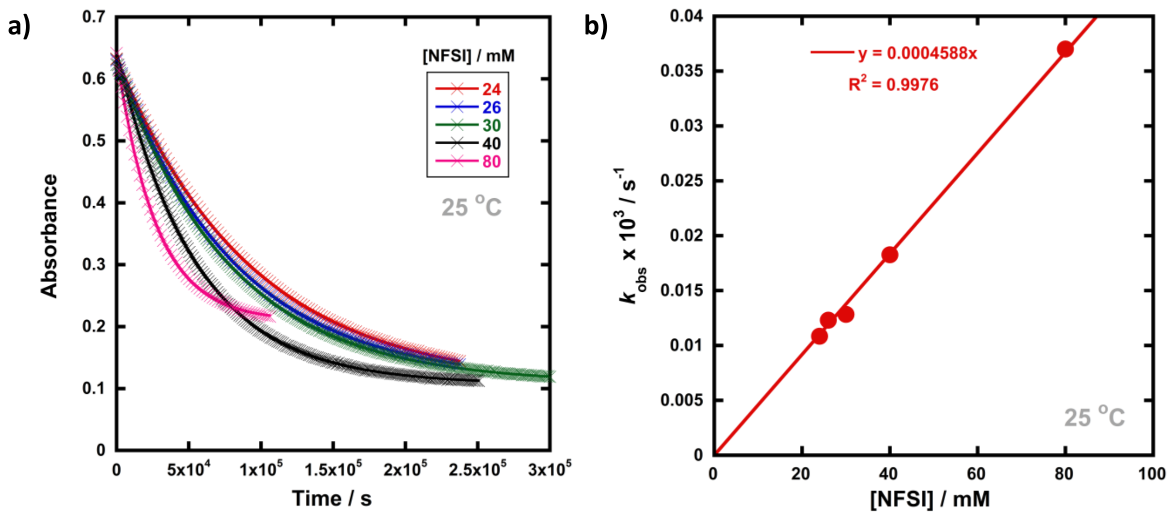

Figure 58: (a) Exponential decays of absorbance of **5a-enol** with different concentrations of NFSI, monitored at λ_{max} = 350 nm. (b) Correlation of k_{obs} with [NFSI].

Table 20: k_{obs} values at different concentrations of NFSI at 25 °C. Errors are standard error values. The concentration of **5a-enol** is 0.025 mM.

Experiment	Ratio of [NFSI] to [5a-enol]	[NFSI] / mM	[$5a_{total}$] / mM	$k_{obs} \times 10^3$ / s^{-1}
**1**	960:1	24.0	0.5	0.01085 ± 0.00003
**2**	1040:1	26.0	0.5	0.01231 ± 0.00003
**3**	1200:1	30.0	0.5	0.01285 ± 0.00002
**4**	1600:1	40.0	0.5	0.01827 ± 0.00004
**5**	3200:1	80.0	0.5	0.0370 ± 0.0001

3.14.2 Fluorination of 5b

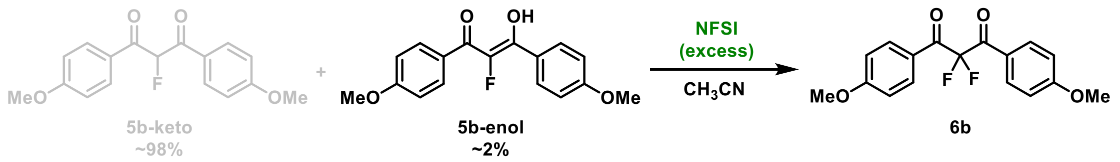

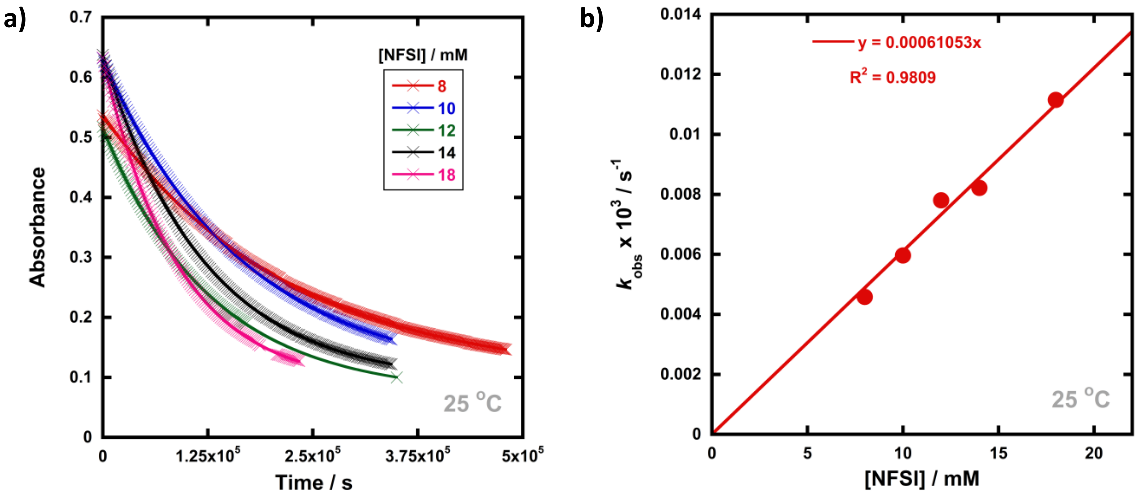

Figure 59: (a) Exponential decays of absorbance of **5b-enol** with different concentrations of NFSI, monitored at 380 nm. (b) Correlation of k_{obs} with [NFSI].

Table 21: k_{obs} values at different concentrations of NFSI at 25 °C. Errors are standard error values. The concentration of **5b-enol** is 0.01 mM.

**Experiment**	**Ratio of [NFSI] to [5b-enol]**	**[NFSI] / mM**	**[5b$_{total}$] / mM**	$k_{obs} \times 10^3$ / s^{-1}
**1**	800:1	8.0	0.5	0.00458 ± 0.00001
**2**	1000:1	10.0	0.5	0.00596 ± 0.00001
**3**	1200:1	12.0	0.5	0.00780 ± 0.00002
**4**	1400:1	14.0	0.5	0.00822 ± 0.00001
**5**	1800:1	18.0	0.5	0.01113 ± 0.00002

3.14.3 Fluorination of 5d

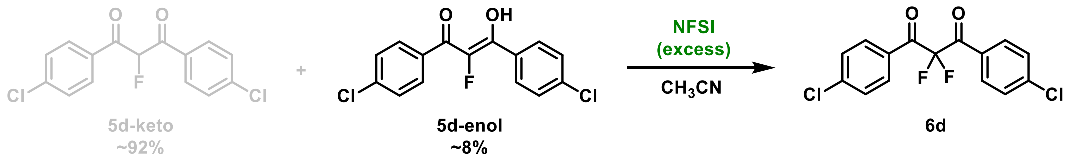

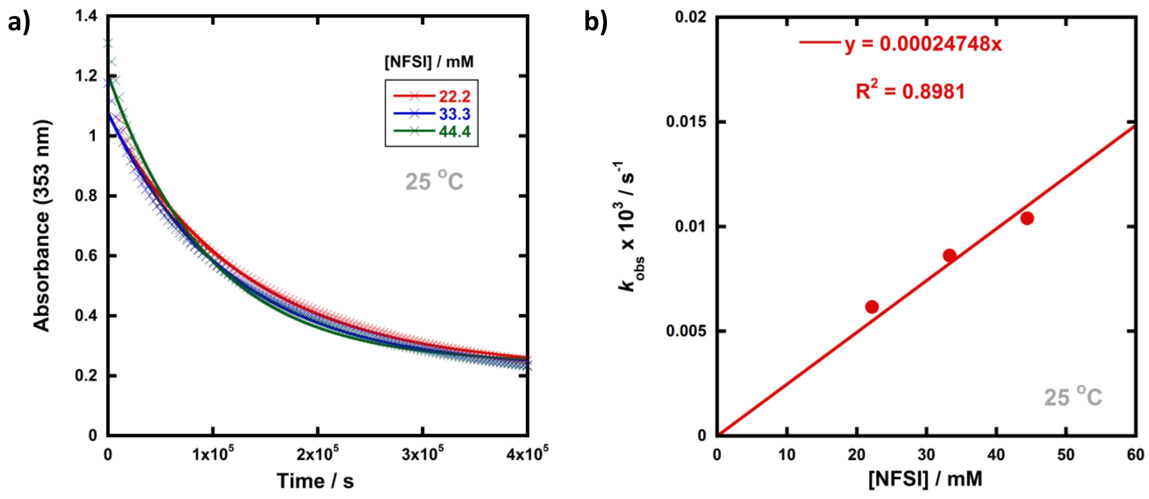

Figure 60: (a) Exponential decays of absorbance of **5d-enol** with different concentrations of NFSI, monitored at λ_{max} = 353 nm. (b) Correlation of k_{obs} with [NFSI].

Table 22: k_{obs} values at different concentrations of NFSI at 25 °C. Errors are standard error values. The concentration of **5d-enol** is 0.04 mM.

Experiment	Ratio of [NFSI] to [5d-enol]	[NFSI] / mM	[$5d_{total}$] / mM	$k_{obs} \times 10^3$ / s^{-1}
**1**	555:1	22.2	0.5	0.0062 ± 0.0001
**2**	833:1	33.3	0.5	0.0086 ± 0.0002
**3**	1110:1	44.4	0.5	0.0104 ± 0.0002

3.14.4 Hammett correlations

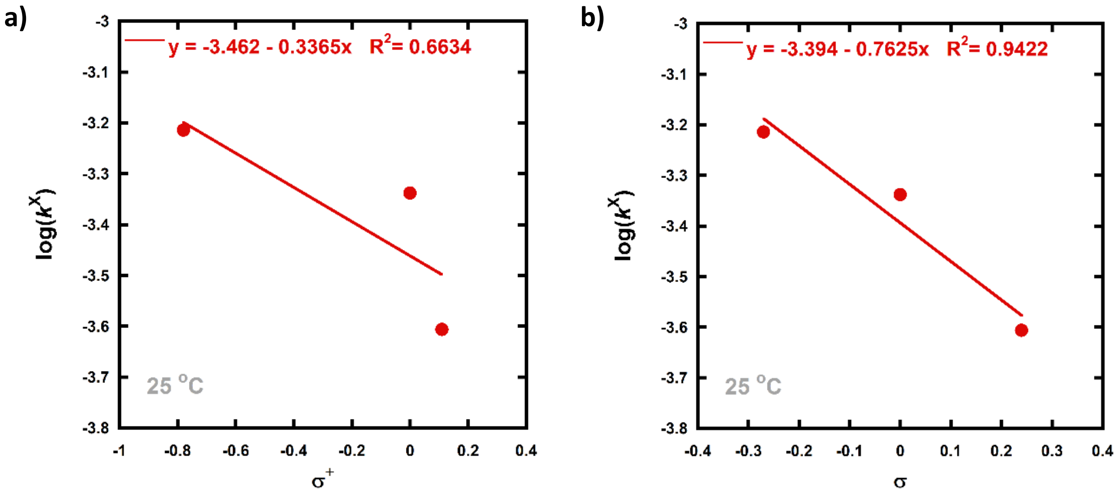

Figure 61: Hammett correlations corresponding to fluorination of **5a**, **5b** and **5d** by NFSI. All rate constants were obtained in MeCN at 25 °C, and are plotted against a) σ_p^+ values, and b) σ_p values.

The use of σ_p values in the construction of the Hammett plot (Figure 61b) gave better correlations than with σ_p^+ values (Figure 61a). However, more data points would be required in order to make valid conclusions from these correlations.

3.15 Kinetics of fluorination of 5a-enol by Selectfluor™ with water

3.15.1 With 20% water in MeCN

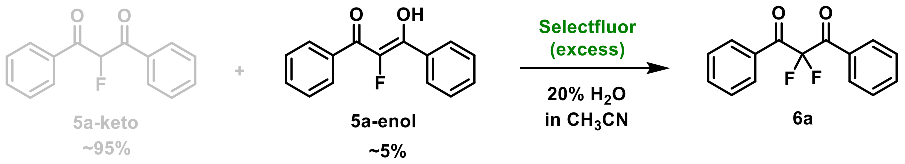

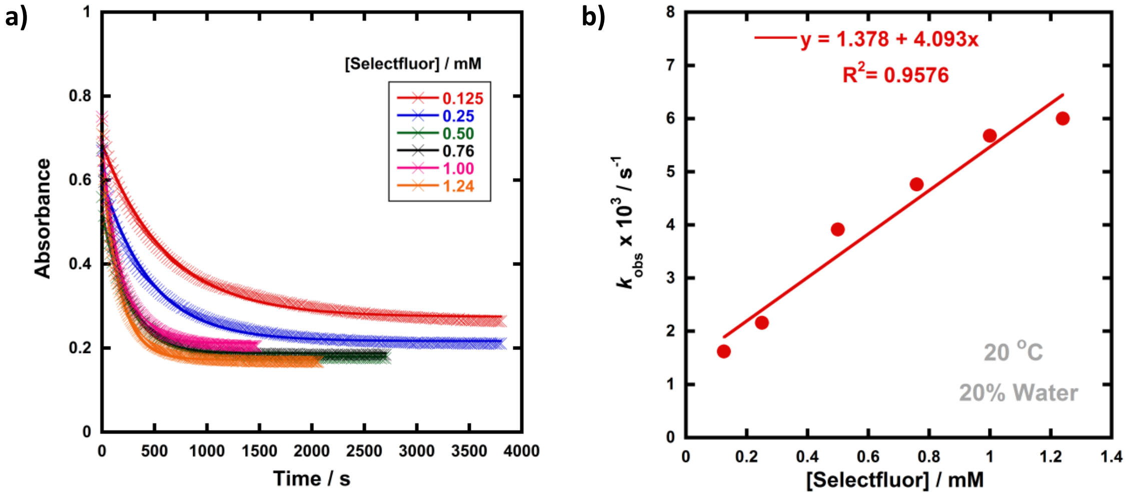

Figure 62: (a) Decays of absorbance of **5a-enol** with different concentrations of Selectfluor™, with 20% water in MeCN at 20 °C. (b) Correlation of k_{obs} with [Selectfluor™].

Table 23: k_{obs} values at different concentrations of Selectfluor™ at 20 °C. Errors are standard error values.

Experiment	Ratio of [Selectfluor] to [5a-enol]	[Selectfluor] / mM	[$5a_{total}$] / mM	$k_{obs} \times 10^3$ / s^{-1}
**1**	5:1	0.125	0.5	1.62 ± 0.02
**2**	10:1	0.25	0.5	2.16 ± 0.01
**3**	20:1	0.50	0.5	3.91 ± 0.01
**4**	30.4:1	0.76	0.5	4.76 ± 0.01
**5**	40:1	1.00	0.5	5.68 ± 0.05
**6**	49.6:1	1.24	0.5	6.00 ± 0.06

3.15.2 With 20% water in MeCN: linear analysis

Since the decays in absorbance of **5a-enol** (Figure 62a) were not first-order, and the plot of the fitted k_{obs} values vs. [Selectfluor™] (Figure 62b) did not intercept the origin, the experiments were repeated by monitoring only the first 10% of the fluorination reactions. Plots of ln(A-A_{inf}) against time were constructed, where A = absorbance of **5a-enol** and A_{inf} = absorbance of **5a-enol** at end of reaction. Gradients of the linear trends at each Selectfluor™ concentration gave the k_{obs} values (Table 24). The plot of k_{obs} values vs. [Selectfluor™] gave the second-order rate constant (Figure 63).

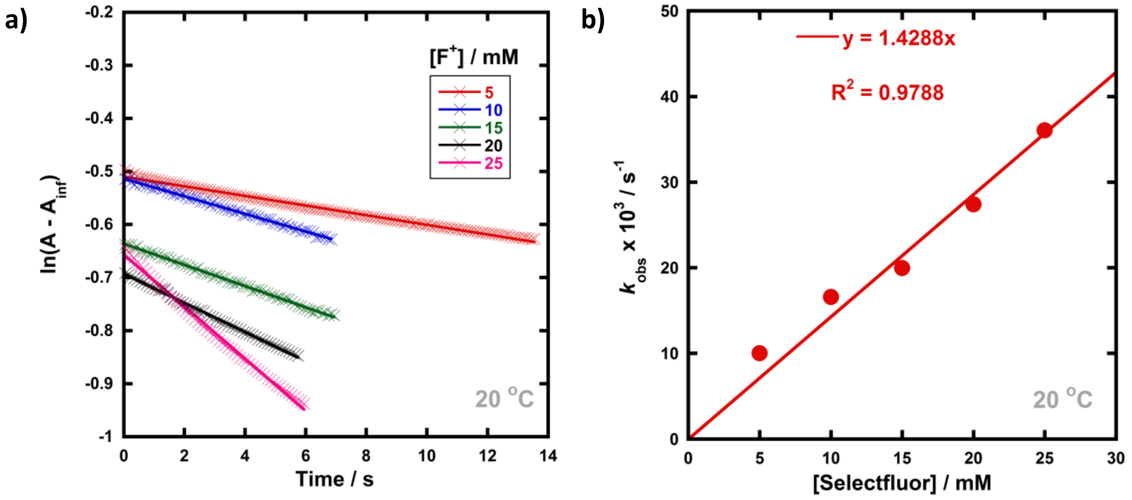

Figure 63: Correlation of k_{obs} from linear fittings with [Selectfluor™].

Table 24: k_{obs} values at different concentrations of Selectfluor™ at 20 °C from linear fittings.

Experiment	Ratio of [Selectfluor] to [5a-enol]	[Selectfluor] / mM	[$5a_{total}$] / mM	$k_{obs} \times 10^3$ / s^{-1}
**1**	200:1	5.0	0.5	10.01
**2**	400:1	10.0	0.5	16.59
**3**	600:1	15.0	0.5	19.96
**4**	800:1	20.0	0.5	27.40
**5**	1000:1	25.0	0.5	36.05

3.16 Kinetics of fluorination of 5a-enol by Selectfluor™ with formic acid

3.16.1 With 3% formic acid in MeCN

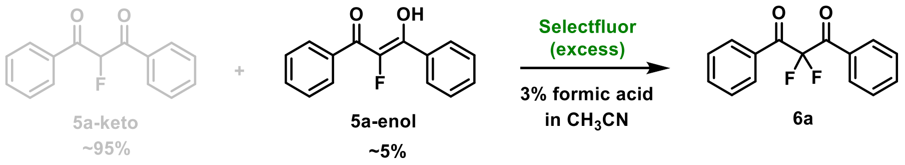

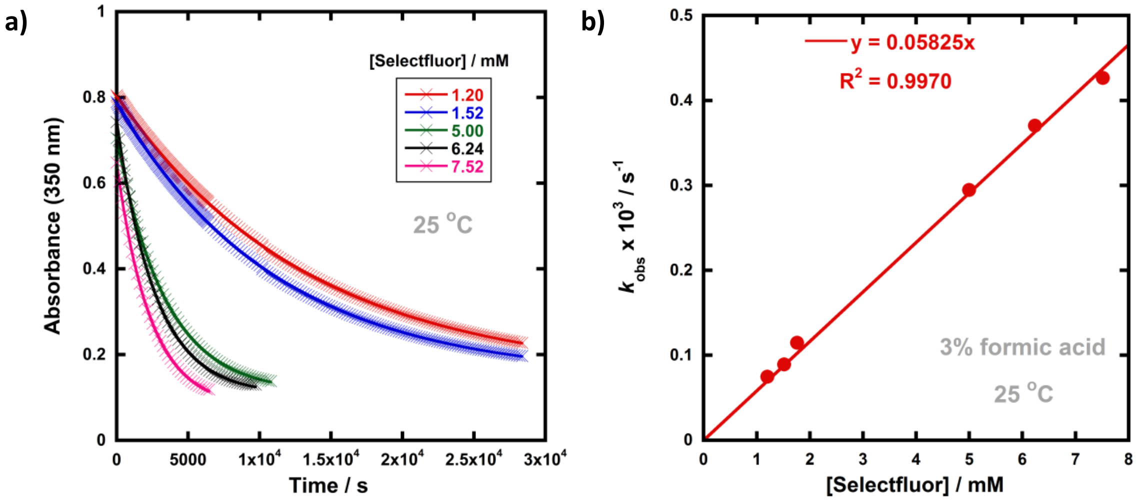

Figure 64: (a) Exponential decays of absorbance with different concentrations of Selectfluor™, with 3% formic acid in MeCN at 25 °C. (b) Correlation of k_{obs} with [Selectfluor™].

Table 25: k_{obs} values at different concentrations of Selectfluor™ at 25 °C. Errors are standard error values.

Experiment	Ratio of [Selectfluor] to [5a-enol]	[Selectfluor] / mM	[5a$_{total}$] / mM	$k_{obs} \times 10^3$ / s^{-1}
**1**	48:1	1.20	0.5	0.0752 ± 0.0001
**2**	61:1	1.52	0.5	0.0898 ± 0.0001
**3**	70:1	1.76	0.5	0.1146 ± 0.0001
**4**	200:1	5.00	0.5	0.2946 ± 0.0008
**5**	250:1	6.24	0.5	0.370 ± 0.001
**6**	300:1	7.52	0.5	0.426 ± 0.002

The second-order rate constant for fluorination of **5a-enol** with Selectfluor™ without additives at 25 °C was 4.37×10^{-2} M^{-1} s^{-1}. The value in the presence of 3% formic acid is 5.83×10^{-2} M^{-1} s^{-1}, which is 1.3-fold higher.

3.16.2 With 5% formic acid in MeCN

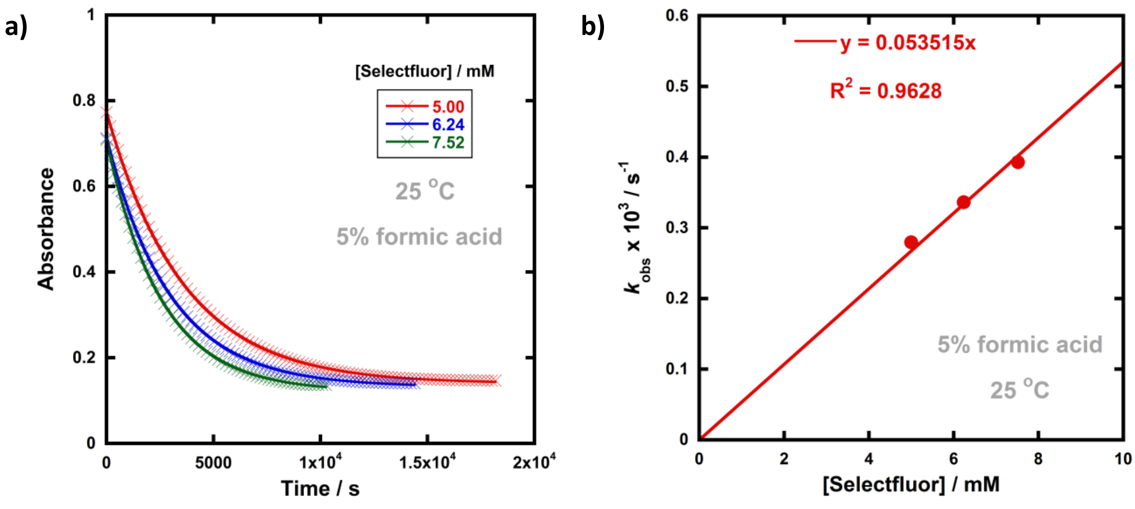

Figure 65: (a) Exponential decays of absorbance with different concentrations of Selectfluor™, with 5% formic acid in MeCN at 25 °C. (b) Correlation of k_{obs} with [Selectfluor™].

Table 26: k_{obs} values at different concentrations of Selectfluor™ at 25 °C. Errors are standard error values.

Experiment	Ratio of [Selectfluor] to [5a-enol]	[Selectfluor] / mM	[$5a_{total}$] / mM	$k_{obs} \times 10^3$ / s^{-1}
**1**	200:1	5.00	0.5	0.2794 ± 0.0006
**2**	250:1	6.24	0.5	0.3362 ± 0.0009
**3**	300:1	7.52	0.5	0.393 ± 0.001

Compared with the second-order rate constant for fluorination of **5a-enol** with Selectfluor™ without additives at 25 °C (4.37×10^{-2} M^{-1} s^{-1}), in the presence of 5% formic acid the rate is 1.2-fold higher (5.35×10^{-2} M^{-1} s^{-1}).

3.16.3 With 20% formic acid in MeCN

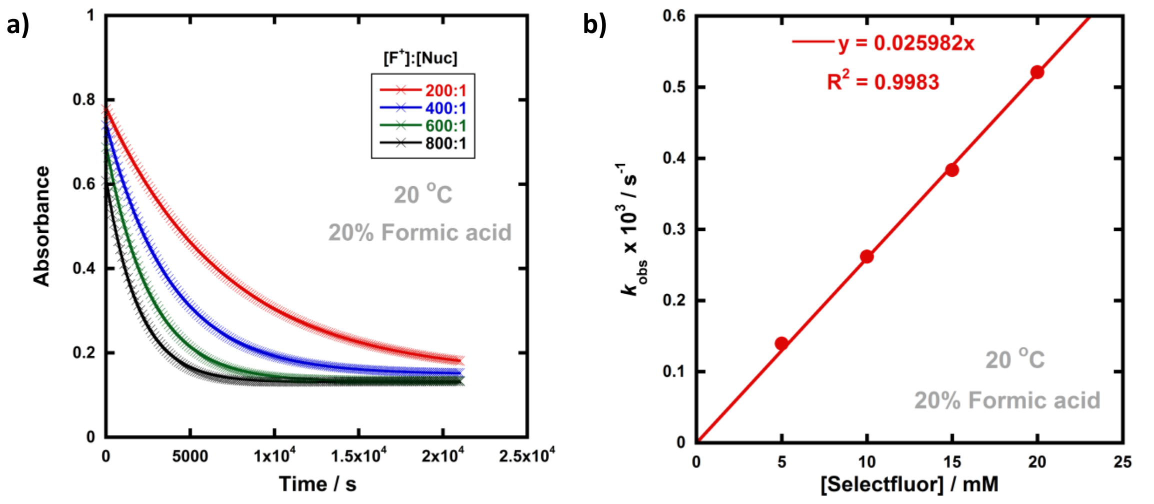

Figure 66: (a) Exponential decays of absorbance with different concentrations of Selectfluor™, with 20% formic acid in MeCN at 20 °C. (b) Correlation of k_{obs} with [Selectfluor™].

Table 27: k_{obs} values at different concentrations of Selectfluor™ at 20 °C. Errors are standard error values.

Experiment	Ratio of [Selectfluor] to [5a-enol]	[Selectfluor] / mM	[$5a_{total}$] / mM	$k_{obs} \times 10^3$ / s^{-1}
**1**	200:1	5.0	0.5	0.1395 ± 0.0002
**2**	400:1	10.0	0.5	0.2617 ± 0.0003
**3**	600:1	15.0	0.5	0.383 ± 0.001
**4**	800:1	20.0	0.5	0.521 ± 0.002

Without additives at 20 °C, the second-order rate constant for fluorination of **5a-enol** by Selectfluor™ (2.95×10^{-2} M^{-1} s^{-1}) is 1.1-fold higher than in the presence of 20% formic acid (2.60×10^{-2} M^{-1} s^{-1}). From these studies, it appears the rate of fluorination is slightly increased by the presence of small quantities of formic acid, although higher concentrations do not have a beneficial effect on the rate.

3.17 Kinetics of fluorination of 5a-enol by Selectfluor™ with $Bu_4N^+ BF_4^-$

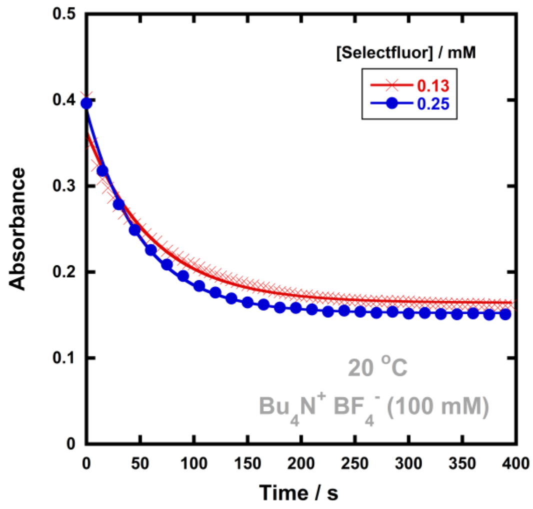

Figure 67: Exponential decays of absorbance of **5a-enol** with different concentrations of Selectfluor™, with $Bu_4N^+ BF_4^-$ (100 mM) in MeCN at 20 °C.

Table 28: k_{obs} values at different concentrations of Selectfluor™ at 20 °C. Errors are standard error values.

Experiment	Ratio of [Selectfluor] to [5a-enol]	[Selectfluor] / mM	$[5a_{total}]$ / mM	$k_{obs} \times 10^3$ / s^{-1}
**1**	5:1	0.13	0.5	16.1 ± 0.4
**2**	10:1	0.25	0.5	19.8 ± 0.4

The rate of fluorination of **5a-enol** by Selectfluor™ (0.25 mM) without additives at 20 °C was estimated to be 7.5×10^{-6} s^{-1} using the results in Section 3.13.1. Compared to the rate of fluorination in the presence of $Bu_4N^+ BF_4^-$, 19.8×10^{-3} s^{-1}, this is a 2640-fold difference in reactivities.

3.18 Kinetics of fluorination of 4a-enol by Selectfluor™ in H_2O/MeCN mixtures

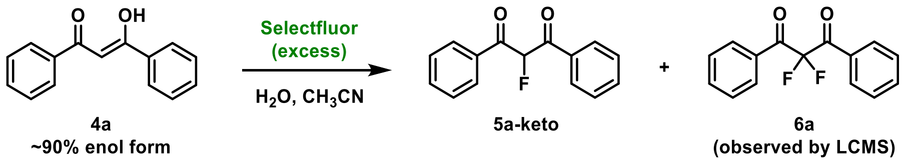

We monitored the kinetics of fluorination of **4a** by Selectfluor™ in water/MeCN mixtures at λ_{max} = 341 nm. However, non-first order kinetics were observed. LC-MS analysis of the reaction mixtures showed the presence of **6a**. Since Figure 68b did not intercept the origin, we conducted experiments using a different approach, described in Section 3.18.2.

3.18.1 With 20% water in MeCN

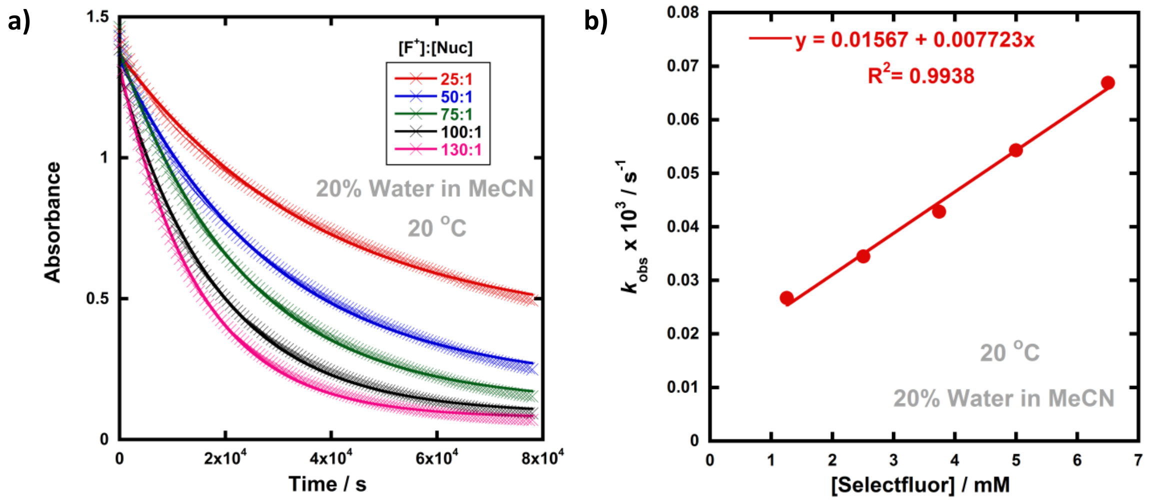

Figure 68: Non-first order kinetics of fluorination of **4a-enol** by Selectfluor™, with 20% water in MeCN at 20 °C.

Table 29: k_{obs} values at different concentrations of Selectfluor™ at 20 °C. Errors are standard error values.

Experiment	Ratio of [Selectfluor] to [4a-enol]	[Selectfluor] / mM	[$4a_{total}$] / mM	$k_{obs} \times 10^3$ / s^{-1}
**1**	25:1	1.25	0.05	0.0267 ± 0.0006
**2**	50:1	2.50	0.05	0.0344 ± 0.0005
**3**	75:1	3.75	0.05	0.0428 ± 0.0005
**4**	100:1	5.00	0.05	0.0543 ± 0.0006
**5**	130:1	6.50	0.05	0.0669 ± 0.0006

3.18.2 With 20% water in MeCN: linear analysis

The first 10% of the reactions were monitored by UV-vis spectrophotometry and plots of ln(A−A_{inf}) *vs*. time were linear. Gradients of the plots at each Selectfluor™ concentration gave the k_{obs} values (Table 30). The plot of k_{obs} values *vs*. [Selectfluor™] gave the second-order rate constant (Figure 69).

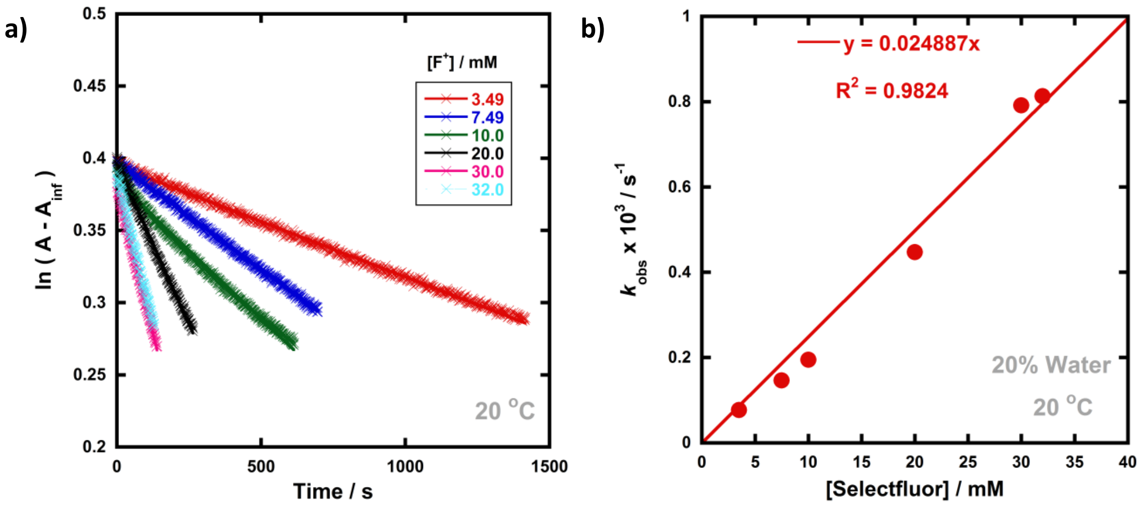

Figure 69: Correlation of k_{obs} from linear fittings with [Selectfluor™].

Table 30: k_{obs} values at different concentrations of Selectfluor™ at 20 °C from linear fittings.

Experiment	Ratio of [Selectfluor] to [4a-enol]	[Selectfluor] / mM	[$4a_{total}$] / mM	$k_{obs} \times 10^3$ / s^{-1}
**1**	70:1	3.49	0.05	0.0769
**2**	150:1	7.49	0.05	0.1467
**3**	200:1	10.0	0.05	0.1947
**4**	400:1	20.0	0.05	0.4474
**5**	600:1	30.0	0.05	0.7916
**6**	640:0	32.0	0.05	0.8130

4. Difluorination of 4a-enol *via* Selectfluor™, 20% water in MeCN-d_3

The graphs below correspond to the experiment discussed in Section 2.4 of the main text, which was monitored by ^{19}F NMR spectroscopy. Integral intensities were converted to concentrations for use in the model.

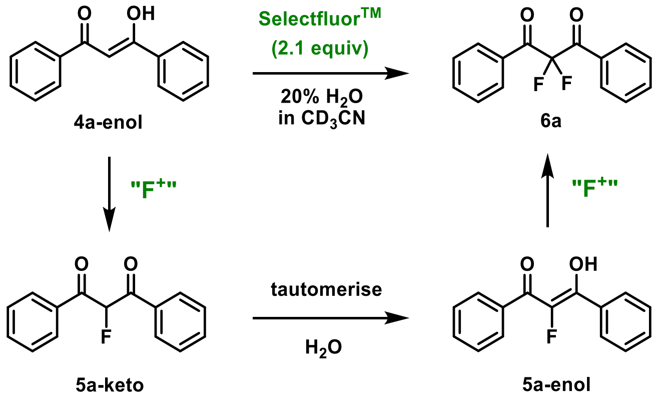

Scheme 1: Full reaction scheme for the conversion of **4a-enol** to **6a** using an excess of Selectfluor™.

Table 31: Quantities used for the reaction of **4a** with Selectfluor™ (2.1 equiv.) in 0.75 mL total volume of solvent at 20 °C.

Experiment	Amount of water / %	[Selectfluor™] / mM	[4a] / mM
**1**	0	62.5	29.7
**2**	20	125.0	59.5

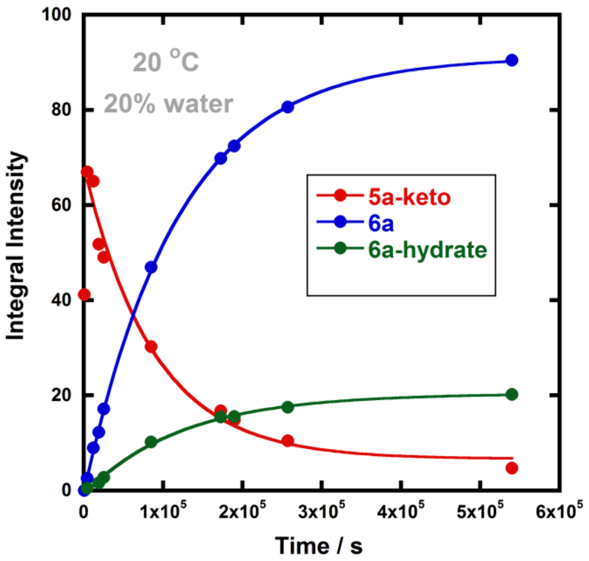

Figure 70: Reaction conducted with 20% water, showing the integrals of peaks corresponding to **5a-keto** (δ = −189.8 ppm) and **6a** (δ = −103.4 ppm) over time, as well as the peak at δ = −111.9 ppm which corresponds to the hydrate of **6a**.

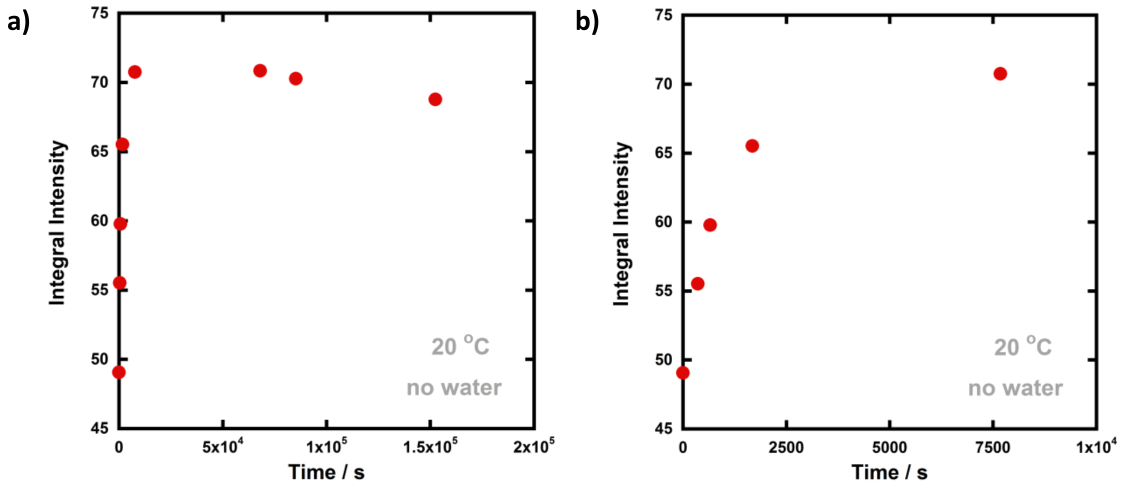

Figure 71: Reaction conducted with 100% MeCN-d_3. Integral of peak corresponding to **5a-keto** over time: (a) Showing full reaction profile; (b) Focussing on the first 2 hours of the reaction.

Due to the wide range of chemical shift of the species present in these NMR experiments (−100 ppm to −200 ppm), peaks towards the edge of the spectra are generally less quantitative with respect to peaks in the centre. This was minimised by increasing the range of the NMR experiments by 30 ppm at both high and low chemical shifts, i.e. acquiring spectra between −70 ppm and −230 ppm. We also increased the relaxation delays to 8 s. An error of ± 10% is associated with NMR integrals, which explains the slightly higher concentration of **5a-keto** produced (65 mM) in the reaction with 20% water than would be expected given the starting concentration of **4a** (59.5 mM).

5. References

1 K. Sato, G. Sandford, K. Shimizu, S. Akiyama, M. J. Lancashire, D. S. Yufit, A. Tarui, M. Omote, I. Kumadaki, S. Harusawa and A. Ando, *Tetrahedron*, 2016, **72**, 1690–1698.

2 N. Rozatian, I. W. Ashworth, G. Sandford and D. R. W. Hodgson, *Chem. Sci.*, 2018, **9**, 8692–8702.

3 K. K. Laali, A. Jamalian and C. Zhao, *Tetrahedron Lett.*, 2014, **55**, 6643–6646.

4 F. Buckingham, A. K. Kirjavainen, S. Forsback, A. Krzyczmonik, T. Keller, I. M. Newington, M. Glaser, S. K. Luthra, O. Solin and V. Gouverneur, *Angew. Chem. Int. Ed.*, 2015, **54**, 13366–13369.

5 G. Stavber and S. Stavber, *Adv. Synth. Catal.*, 2010, **352**, 2838–2846.
